# Mechanism and therapeutic significance of ARV-110 combined with a PDGFR inhibitor for the induction of apoptosis in castration-resistant prostate cancer cells through the ROS/JNK pathway

**DOI:** 10.1038/s41419-026-08718-w

**Published:** 2026-04-10

**Authors:** Yang Fu, Shanshan Sun, Guangxu Liu, Qingzhuo Dong, Yutao Wang, Haoyuan Zheng, Chiyuan Piao, Jianbin Bi

**Affiliations:** 1 Department of Urology, The First Hospital of China Medical University, Shenyang, China; 2 Laboratory of the First Clinical College of China Medical University, Shenyang, China; 3 Department of Pharmacy, The People’s Hospital of Liaoning Province, Shenyang, China; 4 Department of Urology, Chinese Academy of Medical Sciences and Peking Union Medical College, Beijing, China

**Keywords:** Prostate cancer, Prostate cancer

## Abstract

The clinical treatment of castration-resistant prostate cancer (CRPC) is currently a major challenge. This study explored a new combination strategy for CRPC that targeted androgen receptor (AR)-dependent and AR-independent mechanisms. First, the degradation efficiency of AR by ARV-110 was verified. CCK-8 and CellTiter-Glo assays were used to evaluate the viability of CRPC cells after treatment. The combination index of platelet-derived growth factor receptor (PDGFR) inhibitors combined with ARV-110 was calculated using CompuSyn software. Transcriptome sequencing was used to explore the in-depth mechanisms of the combination strategy. Chromatin immunoprecipitation and dual-luciferase reporter assays were used to clarify the transcriptional regulatory relationships. Coimmunoprecipitation was used to evaluate protein interactions. The results showed that ARV-110 significantly promoted AR degradation. The combination of ARV-110 and ponatinib exerted a significant inhibitory and synergistic effect on CRPC cells. The effective targets were AR and PDGFR. The combination of ARV-110 and the PDGFR-selective inhibitor JNJ10198409 effectively induced the apoptosis of CRPC cells. ARV-110 alone promoted the transcription of PDGFA. And the combination strategy further induced JNK signaling pathway activation and promoted cell apoptosis by inhibiting PDGFR activity. Additionally, the substantial accumulation of reactive oxygen species induced by the combination strategy was related to the joint downregulation of catalase by the two drugs through different mechanisms. In conclusion, this study described a new strategy for the treatment of CRPC and clarified the molecular mechanisms of the combination strategy, providing a new theoretical basis for the precision treatment of CRPC.

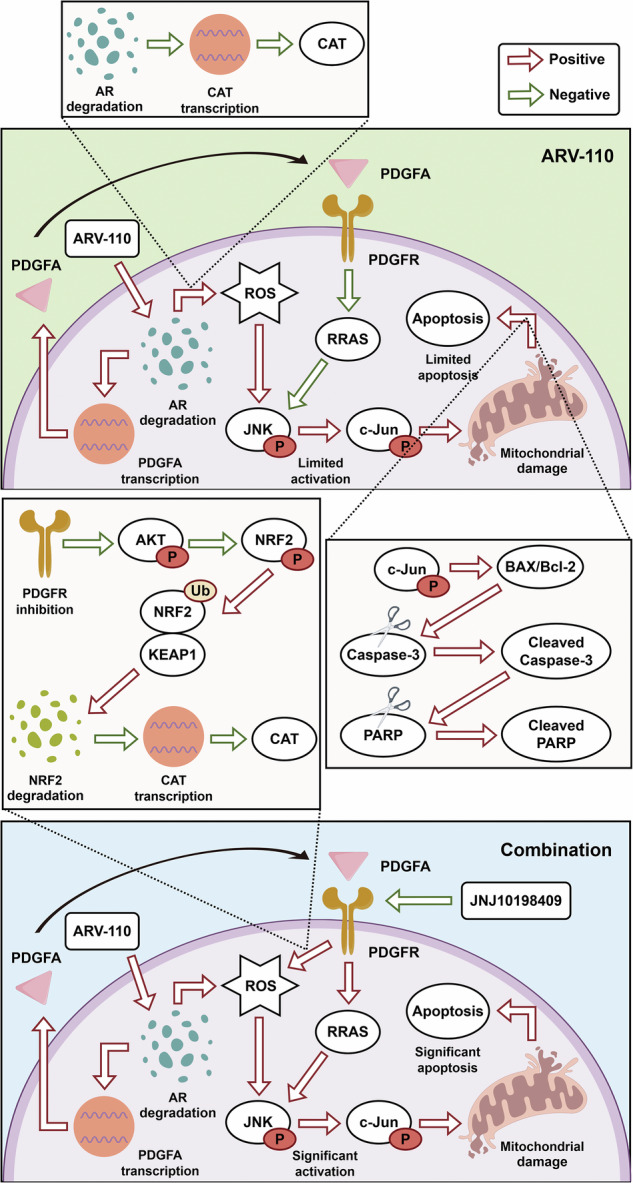

## Introduction

Prostate cancer (PCa) is an androgen-dependent malignant tumor and ranks second in incidence among malignant tumors in males worldwide [1]. The occurrence and progression of PCa depend on the androgen receptor (AR) signaling pathway. In recent years, although the use of androgen deprivation therapy (ADT), such as abiraterone and enzalutamide, has significantly improved the survival rate of PCa patients and has effectively delayed the recurrence of PCa [2], the disease eventually progresses to castration-resistant prostate cancer (CRPC) in most patients.

The mechanisms of CRPC progression can be divided into AR-dependent mechanisms and AR-independent mechanisms. AR-dependent mechanisms include AR mutations, AR gene amplification, AR mRNA alternative splicing, posttranslational modifications of the AR protein, and disorders of AR activators or inhibitors, which cause abnormal activation of the AR signaling pathway [3–5]. AR-independent mechanisms, including the neuroendocrine differentiation of PCa cells [6, 7], activation of PCa stem cells [8], tumor microenvironment remodeling [9], and abnormal activation of other tumor-related signaling pathways [10–12], also deserve attention. Therefore, CRPC is characterized by complex molecular features, high heterogeneity, and a poor prognosis, which pose major challenges in its clinical treatment.

Currently, inhibition of the AR signaling pathway is still considered one of the most effective methods for the treatment of CRPC. However, CRPC does not respond well to conventional ADT and is prone to drug resistance. Compared with the inhibition of target protein function, the direct degradation of tumor progression-related proteins may have more therapeutic advantages [13, 14]. Therefore, Arvinas developed ARV-110, which could target and degrade AR through proteolysis-targeting chimera (PROTAC) technology [15].

In preclinical study, ARV-110 bound the ligand binding domain (LBD) of AR to selectively degrade AR and has exhibited a greater ability to inhibit the growth of PCa cells than enzalutamide, indicating the advantage of eliminating AR rather than inhibiting AR [16]. Although ARV-110 has demonstrated significant therapeutic effects in certain CRPC patients in clinical trials, the overall clinical response rate was still limited (https://ir.arvinas.com). This might be due to the high heterogeneity of CRPC, which indicated that targeting AR alone could not benefit all patients.

Given that AR-independent mechanisms also play important roles in CRPC progression, we hypothesize that a combination of drugs targeting AR (ARV-110) and AR-independent mechanisms can achieve better CRPC treatment effects. Considering that non-AR-targeted PROTAC drugs that have undergone safety evaluation are extremely limited, these drugs cannot be used for large-scale screening. Therefore, we selected 3501 drugs from the Food and Drug Administration (FDA)-Approved & Passed Phase I Drug Library for possible combination with ARV-110. The efficacy and safety of these drugs were relatively reliable, and since these drugs involved different mechanisms, they provided a wide range of screening options. After screening the most suitable combination strategy, we further evaluated the specific mechanisms involved in the inhibition of CRPC cell viability.

## Materials and methods

### Cell culture

The cell lines (PC3, DU145, LNCaP, 22RV1, VCaP, 293T, and AC16) were purchased from the Cell Resource Bank of the Chinese Academy of Sciences (Beijing, China). The complete culture media used were RPMI-1640 medium supplemented with 10% fetal bovine serum (PC3, DU145, LNCaP, and 22RV1), high-glucose DMEM supplemented with 20% fetal bovine serum (VCaP and 293T), and F12 medium supplemented with 10% fetal bovine serum (AC16). All cells were cultured in T25 culture flasks and maintained in an incubator at a constant temperature (5% CO_2_, 37 °C). All the cell lines were authenticated by STR and were free of mycoplasma contamination.

### Western blotting

Total proteins were extracted from cells using RIPA lysis buffer (Epizyme Biotech, Shanghai, China), followed by protein concentration measurement. Then, the proteins were separated by 10% or 15% SDS/PAGE and transferred to 0.2 µm polyvinylidene fluoride (PVDF) membranes. The PVDF membranes were then blocked with 5% skim milk. Next, the membranes were incubated with primary antibodies overnight at 4 °C, after which they were incubated with secondary antibodies for 1 h at 37 °C. An enhanced chemiluminescence (ECL) system (Bio-Rad, California, USA) and ECL luminescent solution (Beyotime, Beijing, China) were used to visualize the protein bands. The intensity of each band was measured using ImageJ 1.53 software. All antibody information was summarized in Table S1.

### Cell apoptosis

Cells were inoculated into 24-well plates, the cell suspension concentration was 5 × 10⁴/ml, and 200 μl of cell suspension and 500 μl of cell culture medium were added to each well. After 24 h, the cell density was ~50–60%. Cells were treated with the corresponding drugs for 12 h (the drugs were prepared using serum-free culture medium at their IC50 concentrations). The cell culture medium was aspirated, 200 μl of Annexin V-mCherry detection solution (Beyotime, Beijing, China) was added, and the mixture was incubated at room temperature in the dark for 20 min. Then, 200 μl of Hoechst staining solution for live cells (Beyotime, Beijing, China) was added, and the mixture was incubated at room temperature in the dark for 10 minutes. The samples were observed and photographed under a fluorescence microscope. ImageJ 1.53 software was used to count cells.

### Transcriptome sequencing

CRPC cells were divided into five groups, a NC group, an enzalutamide group, two single-drug groups, and a combination strategy group, with three biological replicates in each group (three T25 cell culture flasks, cell density >50%). Cells were treated with the corresponding drugs for 48 h. The drugs were prepared using serum-free culture medium, with enzalutamide at a concentration of 10 μM, and other drugs were prepared according to their IC50 concentrations. The samples were collected with TRIzol (Invitrogen, California, USA), quenched with liquid nitrogen, and sent for testing. According to the results of the differential analyses, genes with FDR < 0.05 and |log2FC | > 1 were determined to be differentially expressed genes. Gene set enrichment analysis (GSEA) was used to identify the enriched functions and biological signaling pathways among the groups. Nominal *P* value < 0.05 and FDR < 0.25 were considered to indicate significant differences in the GSEA.

### In vivo experiments with a xenograft model

22RV1 cells (1 × 10^6^) were resuspended and mixed with 100 µl of serum-free medium and 100 µl of matrix gel (Invitrogen, California, USA) and were then subcutaneously injected into the armpits of 6-week-old male nude mice (Vital River Laboratory Animal Technology Co., Beijing, China). When the tumor size reached 0.3 cm after 14 days, the nude mice were randomly assigned to groups, with 5 nude mice per group. Then, oral administration of the drugs was performed. All drug concentrations were shown in Table S3. The concentrations of all drugs in the nude mice were based on published literatures [17–20]. The minimum dose of JNJ10198409 in a previous study was 25 mg/kg, but considering the potential toxicity of JNJ10198409, we adjusted the dose to 10 mg/kg. The drug was administered once daily, and the weight changes in the nude mice were recorded every 2 days. After 14 days, blood from the nude mice was collected for evaluation of biochemical indices. Finally, the nude mice were sacrificed, the tumor weights and body weights of the nude mice were measured. Heart, liver, spleen, lungs, and kidneys were collected from the nude mice, fixed in 10% neutral buffered formalin, dehydrated, and embedded in paraffin. Then, 5 µm tissue sections were cut and stained with hematoxylin and eosin (HE). Optical microscopy and microphotography were performed on tissue sections from each organ under investigation. No blinding was performed in the in vivo experiments.

### Statistical analyses

The data were presented as the mean ± SD from three different experiments. GraphPad Prism 10.1.2 software was used for the statistical analyses. Unpaired t tests (two-sided) were used to test the differences between two groups, and the F tests were performed to test for homogeneity of variance. One-way ANOVA with multiple comparisons was used to test the differences between multiple groups, and the Brown–Forsythe tests were performed to test for homogeneity of variance. Nonparametric tests were used for non-normal distribution data. Subsequently, the effect sizes, including Cohen’s d (unpaired t test), ω² (one-way ANOVA), and ε² (nonparametric test), were calculated (Table S4). *P* < 0.05 was considered statistically significant. All the primers used for PCR were provided by BGI Genomics (Beijing, China), and the sequences were provided in Table S5. Additional methods were presented in the SUPPLEMENTAL MATERIALS AND METHODS.

## Results

### AR degradation efficiency of ARV-110 and screening for combination strategies with significant inhibitory effects on CRPC cells

**Fig. 1:**
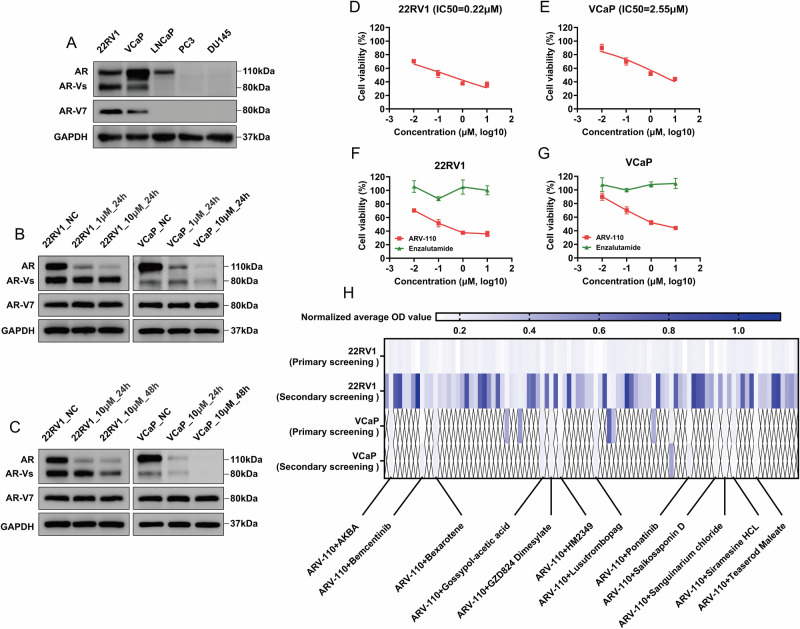
Screening of drug strategies combined with ARV-110 to inhibit CRPC cell viability based on the FDA-approved and Passed Phase I Drug Library. **A** AR, AR-Vs and AR-V7 were expressed in 22RV1 and VCaP cells. **B**, **C** ARV-110 significantly degraded AR. With increasing concentration and time, ARV-110 could degrade AR-Vs, but had no effect on AR-V7. **D**, **E** The IC50s of ARV-110 were calculated to be 0.22 μM (22RV1) and 2.55 μM (VCaP). Data were presented as the mean ± SD from three biological replicates. **F**, **G** Compared with enzalutamide, the cell viability of 22RV1 and VCaP cells decreased significantly with increasing ARV-110 concentration. Data were presented as the mean ± SD from three biological replicates. **H** After four rounds of drug screening, a total of 12 combination strategies could significantly inhibit CRPC cell viability. Each combination strategy was configured with three replicate wells on a 96-well plate, with three negative control replicate wells containing only serum-free medium on each 96-well plate for data normalization. CRPC castration-resistant prostate cancer, AR androgen receptor, IC50 half-maximal inhibitory concentration, AR-Vs AR-variants.

CRPC cells (22RV1 and VCaP) with positive AR expression and numerous androgen receptor variants (AR-Vs) were included (Fig. 1A, Table S6). ARV-110 significantly degraded AR, and with increasing concentration and time, ARV-110 could degrade AR-Vs with complete or partial LBD, but had no effect on AR-V7, which lacked LBD (Figs. 1B, C, S1A–L). The half-maximal inhibitory concentration (IC50) of ARV-110 was then calculated (Fig. 1D, E). Compared with enzalutamide, ARV-110 resulted in a significant decrease in the viability of 22RV1 and VCaP cells (Fig. 1F, G). Next, combination strategies were screened, and finally, 12 combination strategies were found to significantly inhibit CRPC cell viability (Fig. 1H).

### Evaluation of the combined effect of ARV-110 and the 12 candidate drugs

**Fig. 2:**
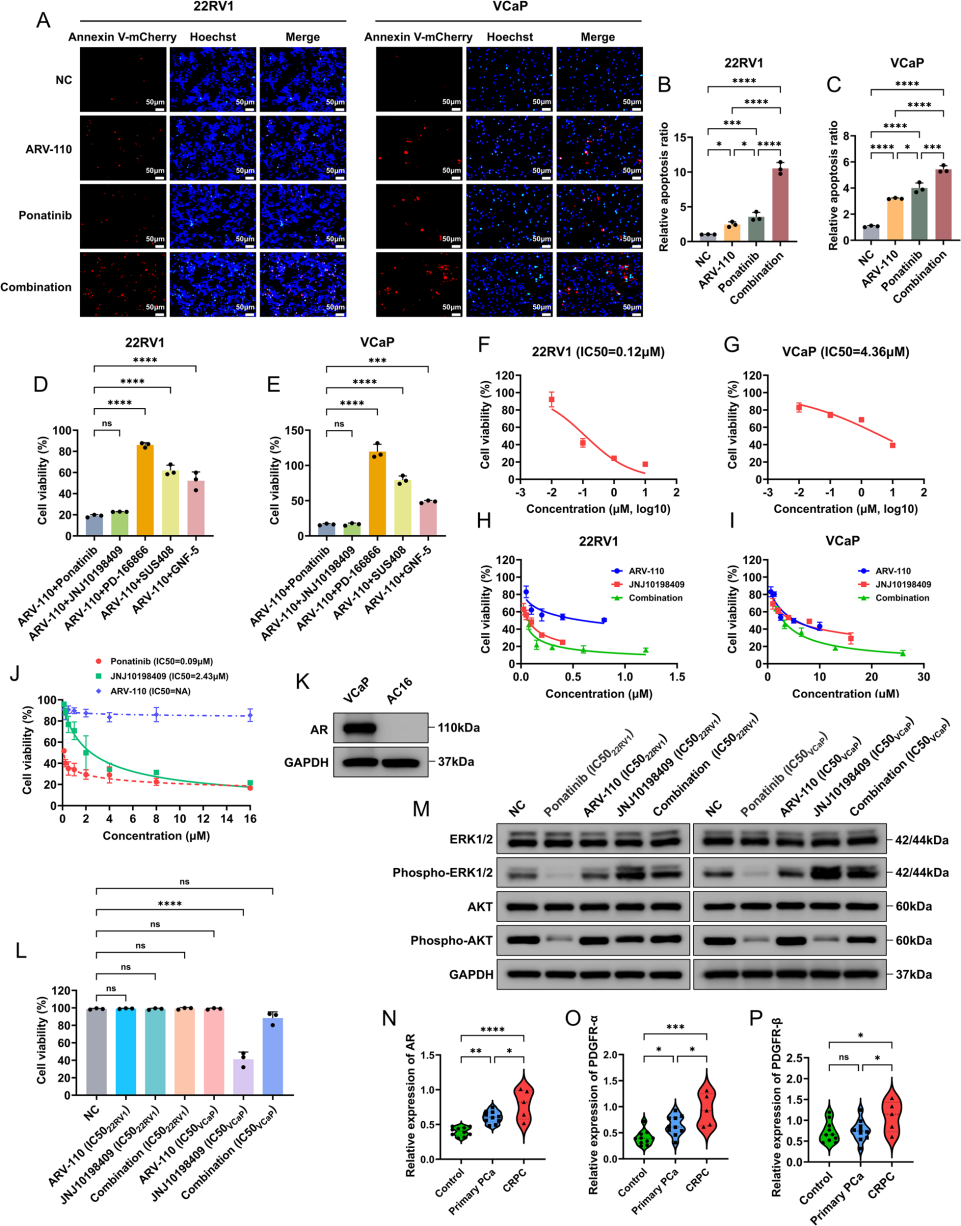
Confirmation of effective targets for ponatinib and preliminary evaluation of the cardiotoxicity of the combination strategy. **A**–**C** The combination of ARV-110 and ponatinib significantly increased the apoptosis of CRPC cells compared with either drug alone. Data were presented as the mean ± SD from three biological replicates. The Brown–Forsythe test *P* values were all > 0.05, satisfying the homogeneity of variance assumption. One-way ANOVA with Turkey multiple comparison corrections was applied. **D**, **E** Compared with other selective inhibitors, only ARV-110 and JNJ10198409 could significantly reduce the viability of CRPC cells. Data were presented as the mean ± SD from three biological replicates. The Brown–Forsythe test *P* values were all > 0.05, satisfying the homogeneity of variance assumption. One-way ANOVA with Turkey multiple comparison corrections was applied. **F**–**I** The IC50s of JNJ10198409 in 22RV1 and VCaP cells were obtained, and then, the dose–effect curves of the ARV-110 and JNJ10198409 combination were completed to calculate the CI values. Data were presented as the mean ± SD from three biological replicates. **J** ARV-110 had no effect on the cell viability of AC16 cells, and the IC50 of JNJ10198409 was significantly increased compared with that of ponatinib. Data were presented as the mean ± SD from three biological replicates. **K** No AR expression was observed in AC16 cells. **L** When the IC50s of the combination strategy of CRPC cell lines were applied to AC16 cells, the cell viabilities after 48 h were 99.53% [Combination (IC50_22RV1_)] and 88.37% [Combination (IC50_VCaP_)]. Data were presented as the mean ± SD from three biological replicates. The Brown–Forsythe test *P* values were all > 0.05, satisfying the homogeneity of variance assumption. One-way ANOVA with Turkey multiple comparison corrections was applied. **M** Ponatinib could significantly reduce the levels of phospho-ERK1/2 and phospho-AKT, while ARV-110 had no significant effect on these levels. JNJ10198409 and the combination strategy could significantly increase the level of phospho-ERK1/2 and slightly reduce the level of phospho-AKT. **N**-**P** The expression of both AR and PDGFR (including PDGFR-α and PDGFR-β) in CRPC tissues (*n* = 5) was significantly higher than that in primary PCa tissues (*n* = 10) and control tissues (*n* = 10). The Kruskal-Wallis tests with Dunn multiple comparison corrections were applied. CRPC castration-resistant prostate cancer, IC50 half-maximal inhibitory concentration, AR androgen receptor, PDGFR platelet-derived growth factor receptor, ERK extracellular regulated protein kinase, CI combination index, PCa prostate cancer. *, *P* < 0.05; **, *P* < 0.01; ***, *P* < 0.001; ****, *P* < 0.0001; ns, not significant.

The synergistic effects of ARV-110 and the 12 candidate drugs were evaluated. The IC50s of the 12 candidate drugs in 22RV1 cells were calculated (Fig. S2A–L). Then, CompuSyn software was used to obtain the combination index (CI) values. Compared with other combination strategies, the synergistic effect of ARV-110 and ponatinib was most significant for both 22RV1 and VCaP cells (Fig. S3A–N, Table S7). Compared with single-drug application, the combination of ARV-110 and ponatinib significantly increased the degree of apoptosis (Fig. 2A–C).

### Confirmation of effective targets for the combination of ARV-110 and ponatinib

Ponatinib can cause serious adverse cardiovascular reactions in the clinical setting due to its pan-inhibitory activity. In particular, ponatinib acts on kinases related to cardiomyocyte survival, such as extracellular regulated protein kinase (ERK) 1/2 and AKT [21, 22]. To minimize cardiotoxicity, the number of targets for ponatinib should be reduced. The common targets of ponatinib are PDGFR, VEGFR2, FGFR1, and BCR-ABL. The corresponding selective inhibitors included JNJ10198409 (PDGFR), PD-166866 (FGFR1), SU5408 (VEGFR2), and GNF-5 (BCR-ABL). After 48 h of combined use of ARV-110 and the above selective inhibitors, only ARV-110 and JNJ10198409 significantly inhibited the viability of CRPC cells with synergistic effects (Fig. 2D-I, Table S8). The above results demonstrated that the mechanism of the synergistic effect of ARV-110 and ponatinib was the simultaneous targeting of AR and PDGFR. In subsequent analyses, the concentrations of ARV-110 and JNJ10198409, whether used alone or in combination, were the IC50 values for both (Table S9).

To estimate the cardiac safety of the combined targeting of AR and PDGFR, we applied ponatinib, JNJ10198409 and ARV-110 to AC16 cells. The results revealed that the IC50 of JNJ10198409 was significantly greater than that of ponatinib. ARV-110 had no effect on cell viability (Fig. 2J) because AC16 cells lacked AR (Fig. 2K). When the IC50s of the combination strategy of CRPC cell lines were applied to AC16 cells, the cell viabilities after 48 h were 99.53% [Combination (IC50_22RV1_)] and 88.37% [Combination (IC50_VCaP_)], respectively, indicating that the combination strategy could effectively inhibit CRPC cells while significantly improving cardiac safety (Fig. 2L). Then, we observed that ponatinib could significantly reduce phospho-ERK1/2 and phospho-AKT. JNJ10198409 and the combination strategy significantly increased phospho-ERK1/2 and slightly decreased phospho-AKT (Figs. 2M, S4A-D).

Next, we detected AR and PDGFR (containing two subtypes, PDGFR-α and PDGFR-β) in tissues. The expression of both AR and PDGFR in CRPC tissues was significantly greater than that in both primary PCa and control tissues (Fig. 2N-P).

**Fig. 3:**
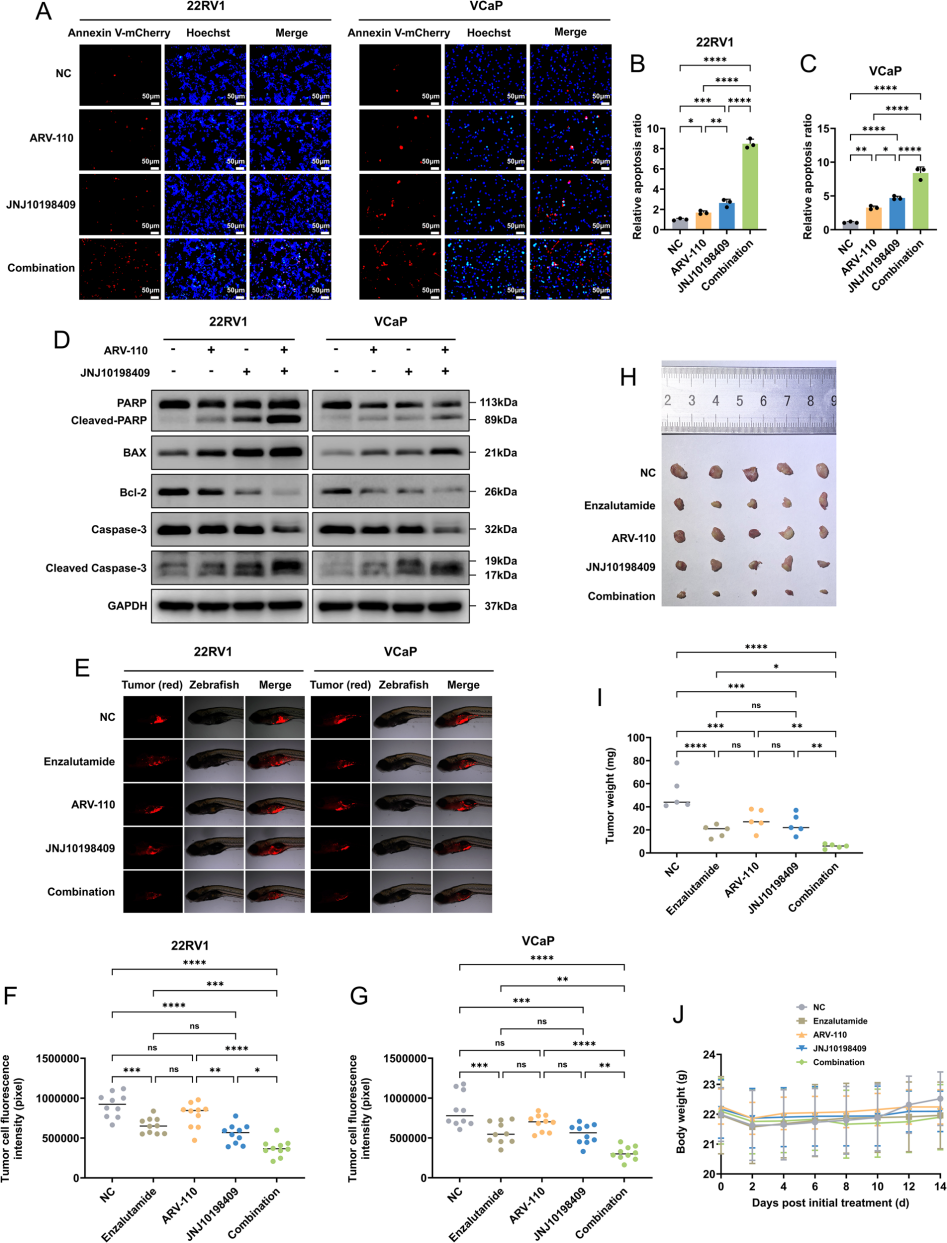
The combination strategy could further induce CRPC cell apoptosis and exert anti-CRPC activity in vitro. **A**–**C** The combined use of ARV-110 and JNJ10198409 significantly increased the apoptosis of CRPC cells compared with single drug use. Data were presented as the mean ± SD from three biological replicates. The Brown–Forsythe test *P* values were all > 0.05, satisfying the homogeneity of variance assumption. One-way ANOVA with Turkey multiple comparison corrections was applied. **D** The combined use of ARV-110 and JNJ10198409 further increased the levels of apoptosis-related proteins. **E**–**G** After 48 h of drug action, the fluorescence intensity results of CRPC cells in each group (*n* = 10/group) of zebrafish showed that the combined use of ARV-110 and JNJ10198409 had the strongest inhibitory effect, significantly better than those of single drug use and enzalutamide. The Kruskal–Wallis tests with Dunn multiple comparison corrections were applied. **H**, **I** The results of the nude mice tumor formation experiments showed that the combined use of ARV-110 and JNJ10198409 had the strongest inhibitory effect on 22RV1 cells and was better than those of single drug use and enzalutamide (*n* = 5/group). The Kruskal–Wallis tests with Dunn multiple comparison corrections were applied. **J** No significant change in the body weights of the nude mice was observed. CRPC, castration-resistant prostate cancer; PARP, poly ADP ribose polymerase; BAX, Bcl-2 associated X protein; Bcl-2, B cell lymphoma 2; caspase-3, cysteinyl aspartate specific protease-3. *, *P* < 0.05; **, P < 0.01; ***, *P* < 0.001*;* ****, *P* < 0.0001; ns not significant.

Compared with each drug alone, the combination strategy significantly increased the percentage of apoptotic CRPC cells and the expression of apoptosis-related proteins (Figs. 3A-D, S5A-F). The further upregulation of cleaved Caspase-3 in the combination strategy indicated that more significant cell apoptosis occurred. Based on the results of BAX/Bcl-2 dysregulation, we hypothesized that apoptosis was caused by increased mitochondrial membrane permeability due to the accumulation of reactive oxygen species (ROS).

We used zebrafish for the initial in vivo experiments. Appropriate drug concentrations were obtained according to the maximum tolerance concentrations (MTCs) (Tables S10-S13). After 48 h, the combination strategy exerted the strongest inhibitory effect (Fig. 3E–G). The xenograft model achieved the same results as the zebrafish model (Fig. 3H, I), and no significant changes were observed in terms of the body weights of the nude mice (Fig. 3J). No significant differences were observed in the biochemical indices of liver function (Fig. S6A, B), cardiac function (Fig. S6C, D), and renal function (Fig. S6E, F). Additionally, no obvious histological abnormalities were observed in the organ sections of the nude mice (Fig. S7).

### AR degradation promoted the transcription of PDGFA and activated the PDGFR signaling pathway

GSEA revealed that ARV-110 could upregulate the PDGFR signaling pathway (Fig. S8A, B). The hub genes of the PDGFR signaling pathway are PDGFR ligands, including PDGFA, PDGFB, PDGFC, and PDGFD. The comprehensive analyses confirmed that only PDGFA was increased in CRPC cells treated with ARV-110 (Fig. S8C–G). Chromatin immunoprecipitation (ChIP‒qPCR) verified that AR could bind to the PDGFA promoter region (Fig. S8H). Based on the binding sites predicted by the JASPAR database, dual-luciferase wild-type and mutant plasmids were constructed (Table S14). The results of the dual-luciferase assay indicated that the dual-luciferase activity was significantly increased when co-transfected with an AR overexpression plasmid and mutant plasmid, suggesting that AR negatively regulated PDGFA transcription (Fig. S8I).

### The combination strategy significantly induced CRPC cell apoptosis through the ROS/JNK pathway

**Fig. 4:**
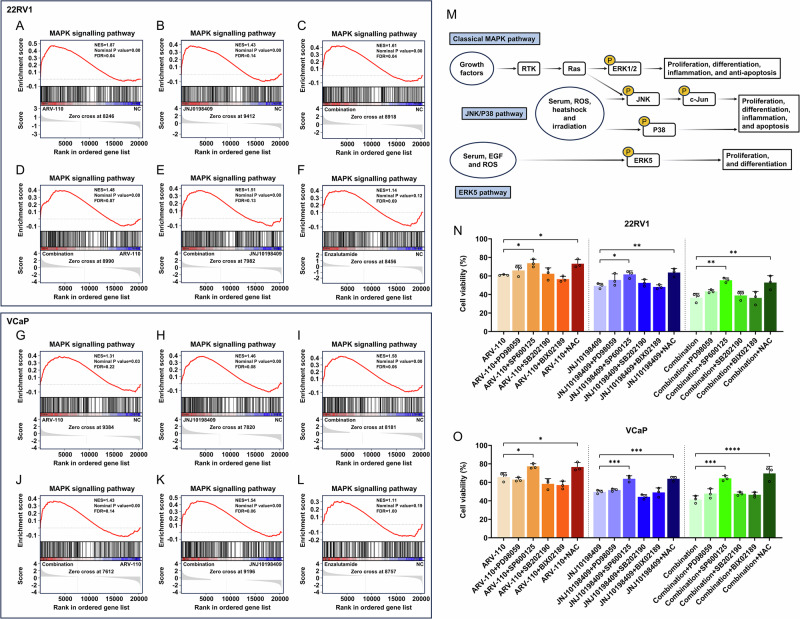
Screening of signaling pathways for the inhibitory effect of combination strategies on CRPC cells. **A**–**L** The GSEA results between the groups based on the transcriptome sequencing data showed that only the MAPK signaling pathway had statistical differences in the inter-group GSEA, except for the enzalutamide group. Transcriptome sequencing involves three biological replicates during the sample preparation stage. **M** The MAPK signaling pathway includes four branch signaling pathways, namely the classical MAPK signaling pathway (the ERK1/2 signaling pathway), the JNK signaling pathway, the P38 signaling pathway, and the ERK5 signaling pathway. **N**, **O** The cell viability was significantly reversed after the addition of SP600125 and NAC, indicating that the combination strategy inhibited CRPC cell viability through the ROS and JNK signaling pathways. Data were presented as the mean ± SD from three biological replicates. The Brown–Forsythe test *P* values were all > 0.05, satisfying the homogeneity of variance assumption. One-way ANOVA with Turkey multiple comparison corrections were applied. CRPC castration-resistant prostate cancer, GSEA gene set enrichment analysis, MAPK mitogen-activated protein kinase, ERK extracellular regulated protein kinase, JNK c-Jun N-terminal kinase, NAC N-acetylcysteine, ROS reactive oxygen species. *, *P* < 0.05; **, P < 0.01; ***, *P* < 0.001*;* ****, *P* < 0.0001.

The intergroup GSEA revealed that only the mitogen-activated protein kinase (MAPK) signaling pathway was significantly different among the groups, except the enzalutamide group (Fig. 4A–L). The MAPK signaling pathway includes 4 branch signaling pathways: the classical MAPK signaling pathway (the ERK1/2 signaling pathway), the c-Jun N-terminal kinase (JNK) signaling pathway, the P38 signaling pathway, and the ERK5 signaling pathway (Fig. 4M). To clarify the specific MAPK branch signaling pathway that inhibits CRPC cells when the combination strategy was used, cells were treated with inhibitors of four branch signaling pathways, including PD98059 (ERK1/2), SP600125 (JNK), SB202190 (P38), and BIX02189 (ERK5) alone or combined with ARV-110 and JNJ10198409. To verify the hypothesis of ROS accumulation, the ROS scavenger N-acetylcysteine (NAC) was added. Cell viability was significantly reversed after the addition of NAC and SP600125 (Fig. 4N, O), which preliminarily indicated that the inhibition of CRPC cell viability was mediated mainly by ROS and the JNK signaling pathway.

**Fig. 5:**
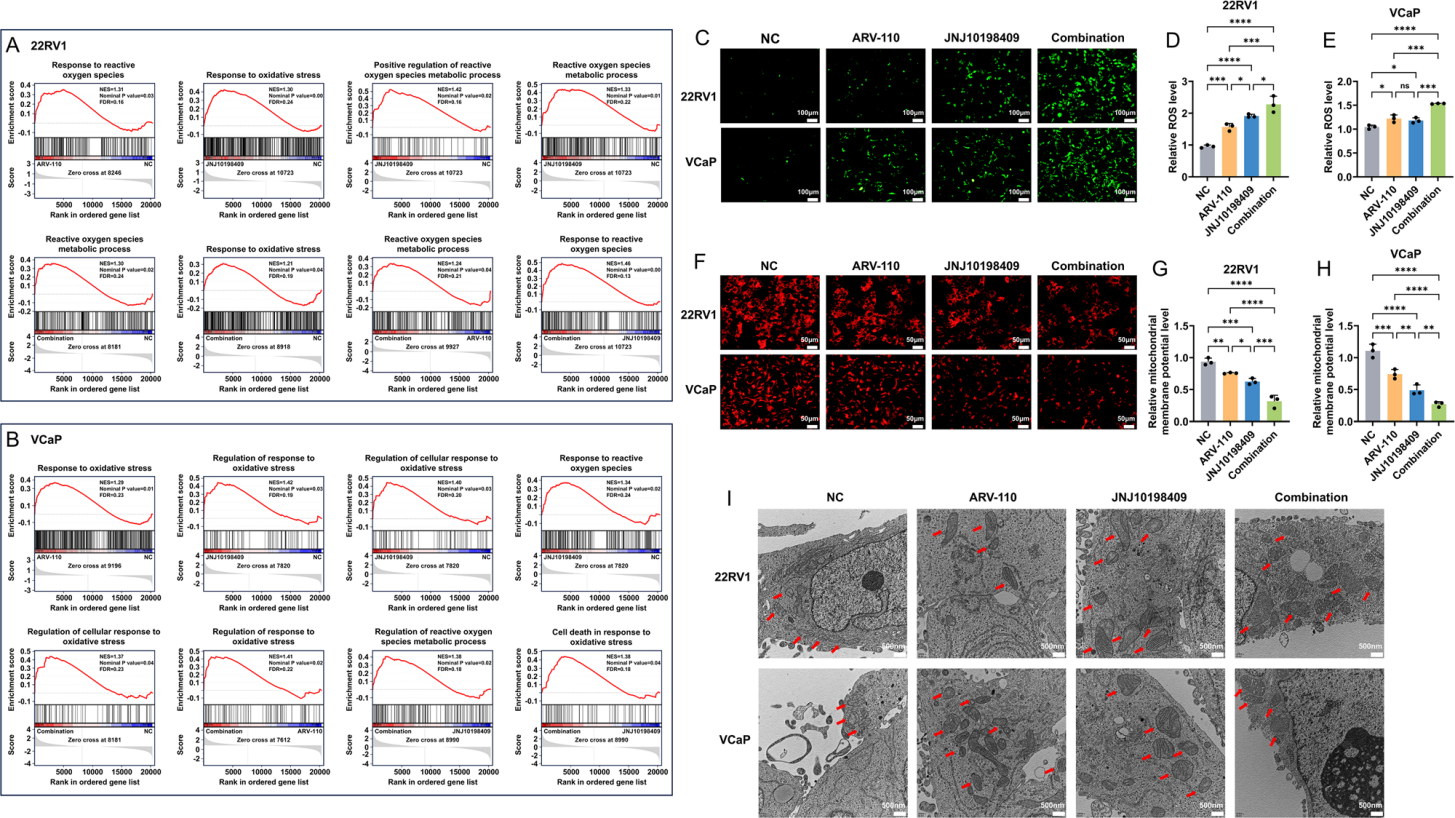
The combination strategy could promote CRPC cell apoptosis by inducing ROS accumulation. **A**, **B** The GSEA results between the groups showed that the functions related to oxidative stress were activated when the drugs were used alone and in combination, and the combined use resulted in higher oxidative stress levels. Transcriptome sequencing used three biological replicates during the sample preparation stage. **C**–**E** The results of CM-H2DCFDA showed that the combination strategy could significantly increase the accumulation level of ROS. Data were presented as the mean ± SD from three biological replicates. The Brown–Forsythe test *P* values were all > 0.05, satisfying the homogeneity of variance assumption. One-way ANOVA with Turkey multiple comparison corrections was applied. **F**–**H** The results of TMRE staining showed that the combination strategy could reduce the mitochondrial membrane potential. Data were presented as the mean ± SD from three biological replicates. The Brown–Forsythe test *P* values were all > 0.05, satisfying the homogeneity of variance assumption. One-way ANOVA with Turkey multiple comparison corrections was applied. **I** TEM revealed that the combination strategy caused severe damage to mitochondria in CRPC cells, with significant mitochondrial swelling and almost complete disappearance of cristae. The red arrows indicated typical mitochondrial morphology. TEM included three biological replicates during the sample preparation phase. CRPC, castration-resistant prostate cancer; GSEA, gene set enrichment analysis; ROS, reactive oxygen species; TEM, transmission electron microscopy. *, *P* < 0.05; **, *P* < 0.01; ***, *P* < 0.001; ****, *P* < 0.0001; ns not significant.

The GSEA between the groups revealed that the combination strategy resulted in the greatest number of functions related to ROS (Fig. 5A, B). In addition, the combination strategy could significantly increase ROS accumulation and mitochondrial membrane potential (MMP) levels (Fig. 5C–H). Transmission electron microscopy (TEM) revealed that the combination strategy caused severe damage to mitochondria in CRPC cells, with significant mitochondrial swelling and almost complete disappearance of cristae (Figs. 5I, S9A–H).

Next, the activity of the JNK signaling pathway was evaluated. According to the transcriptome sequencing data, most of the comparisons between the groups revealed changes in the Ras family among JNK signaling pathway-related genes (Fig. S10A, B). The Ras family includes MRAS, RRAS, KRAS, HRAS, NRAS, and RRAS2, and of these, only RRAS exhibited significant differences in expression among the groups. RRAS expression decreased when ARV-110 was used alone, but increased when JNJ10198409 was used alone or in the combination strategy (Fig. S10C–H).

We found that ARV-110, which upregulated PDGFA to activate PDGFR, could reduce RRAS expression and inhibit the JNK signaling pathway, ultimately limiting ROS-induced JNK signaling pathway activity. And the combination strategy significantly further promoted RRAS and JNK signaling pathway activation (Figs. S10I, S11A-F). Stable CRPC cell lines with RRAS knockdown and overexpression were constructed (Figs. S10J-K, S12A-D). After RRAS knockdown, the JNK signaling pathway was suppressed and was not restored after the addition of JNJ10198409 (Figs. S10L, S12E-J). However, after RRAS overexpression, the JNK signaling pathway was significantly activated (Figs. S10M, S12K-P).

### The combination strategy led to significant accumulation of ROS via CAT reduction

**Fig. 6:**
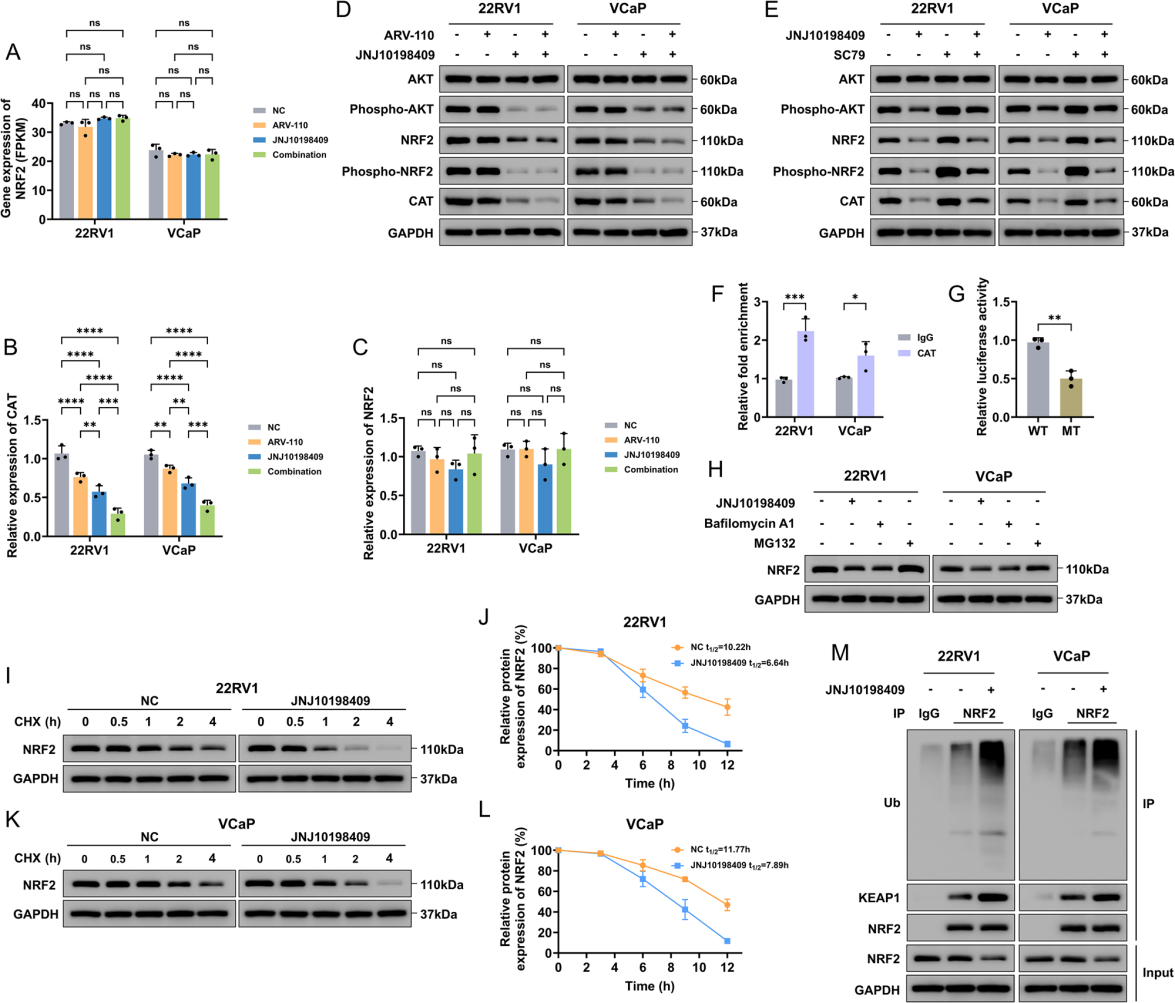
ARV-110 and JNJ10198409 induced ROS accumulation by reducing CAT expression through different mechanisms. **A** No significant changes in the NRF2 mRNA level were observed based on the transcriptome sequencing data. Data were presented as the mean ± SD from three biological replicates. The Brown–Forsythe test *P* values were all > 0.05, satisfying the homogeneity of variance assumption. One-way ANOVA with Turkey multiple comparison corrections were applied. **B**, **C** The qRT–PCR assays were used to verify the analysis results of CAT and NRF2 transcriptome sequencing. Data were presented as the mean ± SD from three biological replicates. The Brown–Forsythe test *P* values were all > 0.05, satisfying the homogeneity of variance assumption. One-way ANOVA with Turkey multiple comparison corrections were applied. **D** In the JNJ10198409 group and the combination strategy group, phospho-AKT, NRF2 and phospho-NRF2 were decreased significantly, but ARV-110 alone did not affect phospho-AKT, NRF2 and phospho-NRF2. **E** When the AKT agonist SC9 was used, the levels of phospho-AKT, NRF2, and phospho-NRF2 were significantly upregulated. When JNJ10198409 was used in combination with SC9, the decrease in the levels of phospho-AKT, NRF2, and phospho-NRF2 caused by the application of JNJ10198409 alone was reversed. **F**, **G** ChIP‒qPCR and dual luciferase reporter assays in CRPC cells verified that AR could bind to the CAT promoter region and play a positive transcriptional regulatory function. Data were presented as the mean ± SD from three biological replicates. The F test *P* values were all > 0.05, satisfying the homogeneity of variance assumption. The unpaired T-tests were applied. **H**–**L** MG132 could reverse the decreased expression of NRF2 caused by JNJ10198409, and JNJ10198409 could significantly reduce the protein stability of NRF2. Data were presented as the mean ± SD from three biological replicates. **M** Co-IP results confirmed that JNJ10198409 promoted the binding of KEAP1 to NRF2, increased the ubiquitination level of NRF2, and then led to the degradation of NRF2. ROS reactive oxygen species, CAT catalase, NRF2 nuclear factor-erythroid 2-related factor 2, CRPC castration-resistant prostate cancer, ChIP chromatin immunoprecipitation, Co-IP coimmunoprecipitation, KEAP1 Kelch-like ECH-associated protein 1. Data are presented as the mean ± SD. *, *P* < 0.05; **, *P* < 0.01; ***, *P* < 0.001; ****, *P* < 0.0001; ns not significant.

An analysis of the expression of ROS-related genes (including NRF2, GPXs, PEXs, NOXs, PPARs, SODs, and CAT) in the transcriptome sequencing data between the groups revealed that only CAT was decreased in the single-drug group and was further decreased when the combination strategy was administered (Figs. S13-S15). Moreover, no significant changes were observed in the NRF2 mRNA levels (Fig. 6A). The literature indicated that increased PDGFR activity could regulate phospho-AKT to activate NRF2, and NRF2 participated in the transcription of antioxidant genes, including CAT [23–25]. The phosphorylation of NRF2 could lead to its dissociation from KEAP1, thereby reducing the ubiquitination and inhibiting the degradation of NRF2 [26, 27]. Further qRT‒PCR assays verified the results of the transcriptome sequencing data (Fig. 6B‒C). In the JNJ10198409 group and the combination strategy group, phospho-AKT, NRF2, and phospho-NRF2 decreased significantly (Figs. 6D, S16A-H). When JNJ10198409 was used in combination with the AKT agonist SC79, the decreases in phospho-AKT, NRF2, and phospho-NRF2 caused by the application of JNJ10198409 alone were reversed (Figs. 6E, S17A-H). According to the above results, we hypothesized that ARV-110 directly downregulates CAT transcription because ARV-110 did not cause changes in phospho-AKT or NRF2. JNJ10198409 could downregulate phospho-AKT, thus affecting the posttranslational modification of NRF2, which results in decreased CAT transcription levels. The combination strategy ultimately led to an intense decrease in CAT through different mechanisms.

ChIP‒qPCR verified that AR could bind to the CAT promoter region (Fig. 6F). Based on the binding sites predicted by the JASPAR database, dual-luciferase wild-type and mutant plasmids were constructed (Table S15). The results of the dual-luciferase assay indicated that the dual-luciferase activity was significantly decreased when co-transfected with an AR overexpression plasmid and mutant plasmid, suggesting that AR positively regulated CAT transcription (Fig. 6G). These findings suggested that when ARV-110 was added, AR degradation directly, rather than through NRF2, regulated the decrease in CAT transcription.

Additionally, our results suggested that MG132 could reverse the decrease in NRF2 expression caused by JNJ10198409 (Fig. 6H) and that JNJ10198409 could significantly reduce the stability of the NRF2 protein (Fig. 6I-L). Coimmunoprecipitation (co-IP) confirmed that JNJ10198409 promoted the binding of KEAP1 to NRF2 and increased the level of NRF2 ubiquitination (Fig. 6M). The degradation of NRF2 also downregulated the transcription level of CAT.

### The addition of NAC and SP600125 reversed CRPC cell apoptosis and JNK signaling pathway activation

**Fig. 7:**
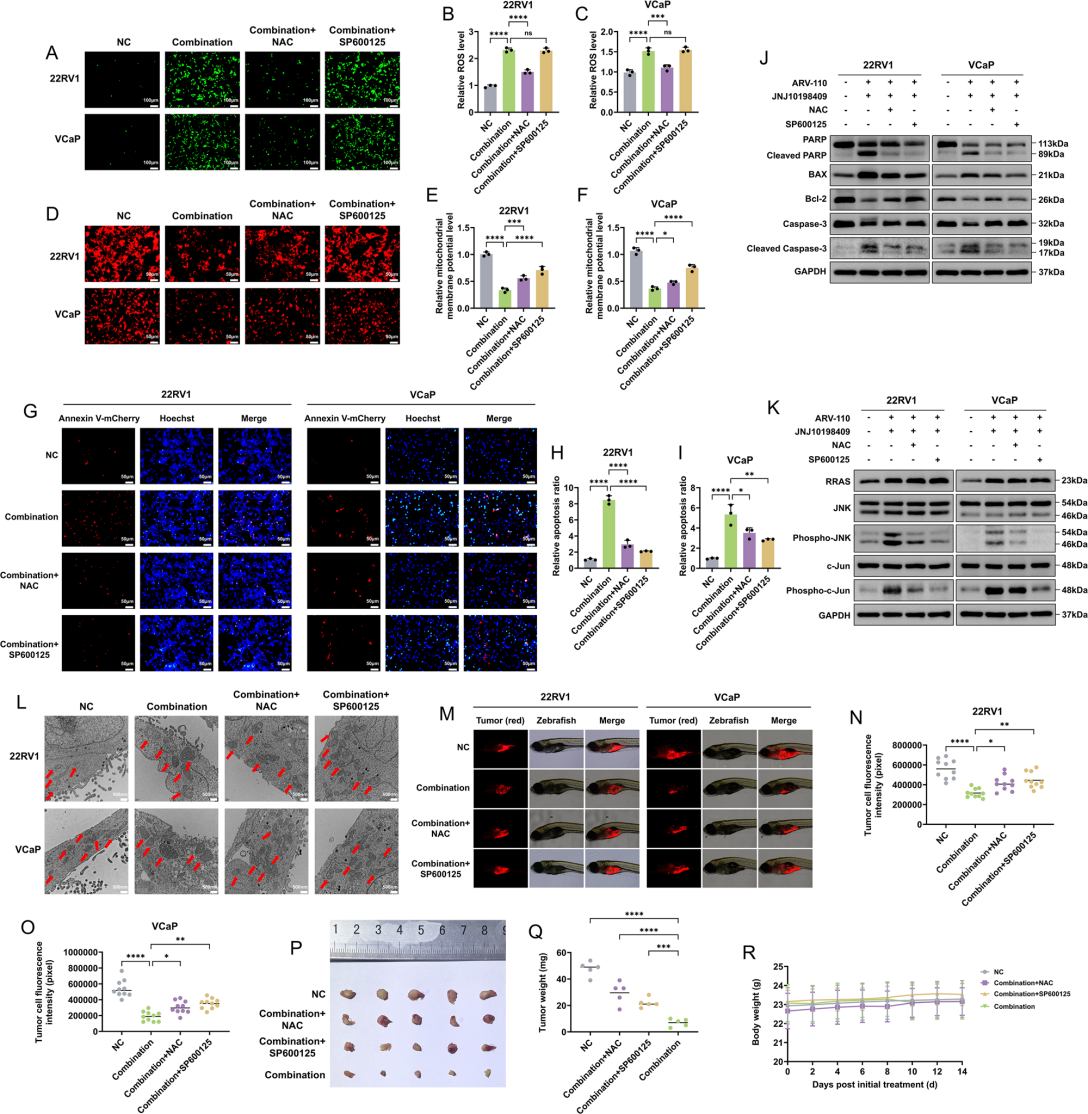
Combination of ARV-110 and JNJ10198409 with the addition of NAC and SP600125 could reverse CRPC cell apoptosis and JNK signaling pathway activation. **A**–**C** After adding NAC and SP600125 to the combination strategy, NAC could significantly clear the ROS accumulated, while SP600125 did not affect the ROS level. Data were presented as the mean ± SD from three biological replicates. The Brown–Forsythe test *P* values were all > 0.05, satisfying the homogeneity of variance assumption. One-way ANOVA with Turkey multiple comparison corrections was applied. **D**–**F** The addition of NAC and SP600125 to the combination strategy could reverse the mitochondrial membrane potential level. Data were presented as the mean ± SD from three biological replicates. The Brown–Forsythe test *P* values were all > 0.05, satisfying the homogeneity of variance assumption. One-way ANOVA with Turkey multiple comparison corrections were applied. **G**–**I** The addition of NAC and SP600125 to the combination strategy could reverse the degree of apoptosis of CRPC cells. Data were presented as the mean ± SD from three biological replicates. The Brown–Forsythe test *P* values were all > 0.05, satisfying the homogeneity of variance assumption. One-way ANOVA with Turkey multiple comparison corrections were applied. **J** The addition of NAC and SP600125 to the combination strategy could reverse the activation of apoptosis-related proteins. **K** The addition of SP600125 and NAC significantly inhibited the phospho-JNK and the phosphor-c-Jun but could not reverse the increase in RRAS expression. **L** TEM showed that NAC and SP600125 also reversed the changes in mitochondrial morphology, with reduced mitochondrial swelling and partial restoration of cristae density. The red arrows indicated typical mitochondrial morphology. TEM included three biological replicates during the sample preparation phase. **M**–**O** In the in vivo experiment, zebrafish were first used (*n* = 10/group). The combination strategy with the addition of NAC and SP600125 could weaken the inhibitory effect on CRPC cells. The Kruskal–Wallis tests with Dunn multiple comparison corrections were applied. **P**–**R** The results of the nude mice tumor formation experiment (*n* = 5/group) showed that the combination strategy with the addition of NAC and SP600125 could weaken the inhibitory effect on 22RV1 cells, and no significant change in the body weights of the nude mice was observed. The Kruskal–Wallis tests with Dunn multiple comparison correction were applied. NAC N-acetylcysteine, CRPC castration-resistant prostate cancer, ROS reactive oxygen species, JNK c-Jun N-terminal kinase, TEM transmission electron microscopy. *, *P* < 0.05; **, *P* < 0.01; ***, *P* < 0.001; ****, *P* < 0.0001; ns not significant.

After the addition of NAC and SP600125 to the combination strategy, NAC significantly decreased the accumulation of ROS, whereas SP600125 did not affect the ROS level (Fig. 7A-C). Additionally, changes in the MMP, the degree of apoptosis, the apoptosis-related proteins and the JNK signaling pathway caused by the combination strategy were reversed, but no decrease in upregulated RRAS was observed (Fig. 7D-K, Figure S18A-L), which suggested that both RRAS and ROS were upstream of the JNK signaling pathway and were independent of each other. TEM showed that NAC and SP600125 also reversed the changes in mitochondrial morphology, with reduced mitochondrial swelling and partial restoration of cristae density (Figs. 7L, S19).

In the initial in vivo experiments, zebrafish were used. Appropriate drug concentrations were also obtained on the basis of the MTC results (Tables S16–S19). After 48 h, the combination strategy with the addition of NAC and SP600125 weakened the inhibitory effect on CRPC cells (Fig. 7M-O). The xenograft model achieved the same results as the zebrafish model, and no significant changes occurred in the body weights of the nude mice (Fig. 7P-R).

## Discussion

Despite continuous progress in the treatment of PCa, the disease is still the fifth leading cause of cancer-related death worldwide, which is mainly due to the progression of the disease to CRPC [28–30]. The treatment and management of CRPC remain extremely challenging, and consequently, most patients have poor prognoses [31]. Since the AR signaling pathway is abnormally activated in CRPC, AR degradation can be used as a potential treatment strategy [32–35].

During progression to CRPC, AR mRNA undergoes alternative splicing, generating AR-Vs. Among all AR variants, AR-V7 has been the most extensively studied. AR-V7 lacks the LBD, enabling it to activate the AR signaling pathway in the absence of androgen [36]. Therefore, AR-V7 is considered a hub driver of enzalutamide resistance, which targets the LBD [37, 38]. The degradation of ARV-110 depended on the LBD of AR, and was therefore ineffective against AR-V7, which was one of the reasons for its limited efficacy in subsequent clinical trials. Additionally, only focusing on AR may lead to compensation by the activation of other signaling pathways, which may result in enhanced tumor cell adaptability and drug resistance [39]. We hope to explore new combination strategies that simultaneously target AR-dependent and AR-independent mechanisms to improve the efficacy of CRPC treatment.

After 4 rounds of screening for combination strategies and evaluation of the combined effects, it was found that the combination of ARV-110 and ponatinib exerted the most significant synergistic effect and more effectively induced CRPC cell apoptosis. Ponatinib is a nonselective multitarget tyrosine kinase inhibitor (TKI). The FDA has approved ponatinib for the treatment of chronic myeloid leukemia and Philadelphia chromosome-positive acute lymphoblastic leukemia [40]. In solid tumors, such as neuroblastoma and non-small cell lung cancer, ponatinib can also significantly inhibit tumor growth and progression [41, 42]. However, to avoid cardiotoxicity, the specific targets of ARV-110 and ponatinib should be identified.

After a series of comprehensive analyses, we found that the effective targets were AR and PDGFR. The combination strategy led to increased apoptosis of CRPC cells. Studies have shown that excessive ROS could induce oxidative stress, leading to mitochondrial damage and cell apoptosis [43–45]. These findings suggested a potential association between CRPC cell apoptosis and ROS accumulation.

GSEA revealed that the PDGFR signaling pathway was significantly activated in the ARV-110 group. PDGFR is an important member of the receptor tyrosine kinases (RTK) family. Previous research results have indicated that after ADT, CRPC cells bypass AR through other mechanisms, such as via dysregulation of the RTK family, and then lead to the abnormal activation of other signaling pathways that promote CRPC cell survival [46–49]. Further analyses confirmed that AR exerted negative transcriptional regulation on PDGFA, the ligand of PDGFR.

Next, our results confirmed the hub role of JNK signaling pathway activation and ROS accumulation in the inhibitory effect on CRPC cells. Studies have shown that the JNK signaling pathway is an important downstream pathway of ROS, which mainly induces cell apoptosis by affecting the mitochondrial function of cells, and that targeting AR or PDGFR alone has been confirmed to cause ROS accumulation [24, 50–52]. The combination strategy led to higher ROS levels, a lower MMP, and higher levels of JNK signaling pathway-related proteins, and thus resulted in a more effective induction of CRPC cell apoptosis.

The inhibition of PDGFR activity upregulated the JNK signaling pathway, which was also mentioned in a recent study [53]. We found that PDGFR inhibited the JNK signaling pathway by affecting RRAS. RRAS was confirmed to have low affinity for Raf due to its unique C-terminal targeting domain, making it difficult to activate the ERK1/2 signaling pathway, but could upregulate the JNK signaling pathway [54–56]. Further results also revealed the potential mediating role of RRAS in significantly inducing apoptosis in the combination strategy. Notably, the activation of the JNK signaling pathway and accumulation of ROS already occurred when ARV-110 and JNJ10198409 were used alone. Therefore, the combination strategy enhanced the existing effects rather than induced new signaling pathways.

Additionally, the combination strategy induced substantial accumulation of ROS by downregulating CAT through two drugs using different mechanisms. Numerous studies have demonstrated a complex signal crosstalk relationship between AR and NRF2 [57–59]. Under ADT stress, oxidative stress was induced, further activating the AR signaling pathway and thereby promoting progression to CRPC. Conversely, oxidative stress promoted the activity of NRF2, which had antioxidant functions. NRF2 could directly act on AR, reducing AR transcription to inhibit CRPC progression. In this study, potent degradation of AR by ARV-110 led to stronger oxidative stress than that induced by ADT, thus exceeding the threshold to induce apoptosis. However, no alterations in NRF2 expression, activity, and upstream AKT activity were detected. This may be because CRPC cell lines undergo prolonged ADT stress, with AKT and NRF2 activities remaining at high levels to clear ROS and gradually becoming independent of AR. We found that the activity of CAT decreased after AR degradation, thereby inducing severe oxidative stress. Other antioxidant-related genes might depend on different coactivators; thus, their transcription remained unaffected. The inhibition of PDGFR activity directly decreased AKT activity, leading to decreased NRF2 expression and activity. These descriptions needed to be confirmed by further investigations.

Currently, no studies on the combination of PROTAC drugs targeting AR with other drugs for the treatment of CRPC existed, but strategies for combining RTK drugs with other drugs to treat CRPC did exist. All relevant combination therapy strategies contained two traditional inhibitors (including preclinical studies and clinical trials). The therapeutic effects of partial strategies were not satisfactory [60, 61]; one strategy had poor safety [62], and the specific mechanisms of other strategies were not yet clear and safety assessments were not conducted [63–67]. However, the combination strategy we proposed combined a novel PROTAC-AR with a PDGFR inhibitor. On the one hand, this strategy targeted AR in CRPC and induced its degradation, exhibiting a stronger anti-CRPC effect than traditional ADT. On the other hand, inhibiting PDGFR also blocked the bypass activation caused by AR degradation. Therefore, our proposed combination strategy had more clear molecular mechanisms and verifiable anticancer efficacy, and preliminary assessments showed a good safety profile. This strategy possessed certain innovativeness and translational potential, but further evaluation through more rigorous clinical trials was still needed.

Finally, this study had some limitations. Due to the lack of reported human pharmacokinetic data for the drugs involved in the study, whether the drug concentrations used in the in vivo experiments were safe for human use remained unknown. Nevertheless, the concentrations used in our in vivo experiments provided an important reference point and comparison for dose design in future Phase I clinical trials in humans. Furthermore, the specific concentration ratio of the two drugs in the combination strategy still needed to be optimized. Finally, the exploration of the PDGFR–RRAS–JNK axis was relatively preliminary, and in-depth research was needed in the future.

In conclusion, we developed a new strategy for the treatment of CRPC, the combined targeting of AR and PDGFR, and clarified the molecular mechanisms by which AR and PDGFR mediate CRPC cell apoptosis, providing a new theoretical basis for the precise treatment of CRPC.

## Supplementary information

Supplementary Materials

Table S2

Table S4

Original Western figures

## Data Availability

The datasets used and/or analyzed during the current study are available from the corresponding author on reasonable request.
