# Supplementary material for: Mechanism and therapeutic significance of ARV-110 combined with a PDGFR inhibitor for the induction of apoptosis in castration-resistant prostate cancer cells through the ROS/JNK pathway
Source: Cell Death Dis. 2026 Apr 10;17(1):463. doi: 10.1038/s41419-026-08718-w (PMC13181112; doi:10.1038/s41419-026-08718-w)
Supplement: Supplementary file 1 — Supplementary Materials [file 41419_2026_8718_MOESM1_ESM.docx]

**SUPPLEMENTAL MATERIALS AND METHODS**

**Patient tissue samples**

From January to December 2024, 5 castration-resistant prostate cancer (CRPC) tissues, 10 pairs of primary PCa tissues and control tissues were collected in the Department of Urology, the First Hospital of China Medical University. After collection, the tissues were stored at -80°C. All tissue samples were histologically evaluated.

**Assessment of cell viability**

CellTiter-Glo reagent (Promega, Wisconsin, USA) or CCK8 reagent (Selleck, Texas, USA) was used to assess cell viability. Cells were inoculated into white 96-well plates (CellTiter-Glo) or 96-well plates (CCK8), the cell suspension concentration was 5×10⁴/ml, and 100 μl of cell suspension was added to each well. After 24 h, the cell density was approximately 50%–60%. After cells were treated with the corresponding drugs, the culture medium was aspirated, and CellTiter-Glo reagent was added to lyse cells. After 10 min, the fluorescence intensity was measured by a fluorescence microplate reader. If CCK8 reagent was added after the culture medium was aspirated, the 96-well plate was placed in the cell culture incubator and incubated for 4 h, after which the absorbance at 450 nm was measured using a microplate spectrophotometer.

**Screening of combination strategies**

On the basis of the FDA-approved & Passed Phase I Drug Library (Selleck, Texas, USA), strategies combining ARV-110 with candidate drugs to inhibit CRPC cell viability were screened. In the process of screening combination strategies, all drugs were prepared using serum-free culture medium, and ARV-110 and 3501 candidate drugs were all used at a concentration of 10 μM in the combination strategies. The list of all candidate drugs was shown in Table S2. The screening process involved 96-well plates, with three replicate wells for each drug and three wells in each 96-well plate without drug as negative control. CCK8 reagent was used to estimate cell viability as described above. In each 96-well plate, 31 drugs were screened, and the OD values of candidate drugs were normalized using the OD values of the negative control wells. To ensure stability, 4 rounds of screening were performed, including 22RV1 primary screening, 22RV1 secondary screening, VCaP primary screening and VCaP secondary screening. Combination strategies with poor stability after each round of screening were excluded. After 4 h of incubation, an average OD value less than 0.2 indicated significant inhibition. The selective inhibitors, including JNJ10198409, PD-166866, SU5408, GNF-5, N-acetylcysteine (NAC), PD98059, SP600125, SB202190 and BIX02189, were obtained from MedChemExpress (New Jersey, USA) and used at a concentration of 10 μM. The AKT agonist SC79 was also purchased from MedChemExpress, and the application concentration was also 10 μM.

**Calculation of** **the half-maximal inhibitory concentration (IC50) and combination index (CI)**

22RV1 cells were inoculated into white 96-well plates, the cell suspension concentration was 5×10⁴/ml, and 100 μl of cell suspension was added to each well. After 24 h, the cell density was in the range of 50%–60%. Then, 22RV1 cells were treated with different concentrations of drugs for 48 h (all drugs were prepared in serum-free culture medium), after which the cell viability was evaluated with CellTiter-Glo reagent, and the half-maximal inhibitory concentration (IC50) values were calculated. On the basis of the calculated IC50 values of the candidate drugs, the drug concentration gradients were established according to the instructions of the CompuSyn software as follows:

Single-drug group 1:

ARV-110: 0, (IC50_1_)/4, (IC50_1_)/2, IC50_1_, 2 (IC50_1_), 4 (IC50_1_)

Single-drug group 2:

Candidate drug: 0, (IC50_2_)/4, (IC50_2_)/2, IC50_2_, 2 (IC50_2_), 4 (IC50_2_)

Combination strategy group:

ARV-110 + Candidate drug: 0, (IC50_1_+IC50_2_)/4, (IC50_1_+IC50_2_)/2, IC50_1_+IC50_2_, 2 (IC50_1_+IC50_2_), 4 (IC50_1_+IC50_2_)

After 48 h, cell viability was assessed using CellTiter-Glo, and the CI values of the combination strategies were calculated using CompuSyn software. CI values were defined as follows: <0.10 for very strong synergy, 0.10–0.30 for strong synergy, 0.30–0.70 for synergy, 0.70–0.85 for moderate synergy, 0.85–0.90 for slight synergy, 0.90–1.10 for an additive effect, 1.10–1.20 for slight antagonism, 1.20–1.45 for moderate antagonism, 1.45–3.30 for antagonism, 3.3–10 for strong antagonism, and >10 for very strong antagonism. After synergistic strategies in 22RV1 cells were identified using CI values, the CI values for these strategies were recalculated in VCaP cells using the same methods.

**Q****uantitative real‐time PCR (qRT–PCR) assay**

RNA was extracted from tissues and cells using TRIzol reagent (Invitrogen, California, USA) according to the manufacturer's instructions. Reverse transcription was performed using Prime Script RT Master Mix (Takara, Osaka, Japan), and qRT–PCR was performed using a SYBR premix ExTaq™ kit (Takara, Osaka, Japan). GAPDH was used as an internal control. Relative gene expression was calculated using the 2^−ΔΔCT^ method.

**Reactive oxygen species (ROS) detection**

Cells were seeded in white 96-well plates (Beyotime, China) and 96-well plates. The cell suspension concentration was 5×10⁴/ml, and 100 μl of cell suspension was added to each well. After 24 h, the cell density was approximately 50%–60%. The corresponding drugs were added for 4 h. The culture medium was aspirated, and 50 μl of CM-H2DCFDA probe (Beyotime, China) was added. Cells were incubated in a cell culture incubator at 37°C for 20 min. The ROS levels of cells plated in white 96-well plates were measured using a fluorescence microplate reader with a 495 nm excitation wavelength and 530 nm emission wavelength. Cells plated in 96-well plates were observed and photographed using a fluorescence microscope.

**Mitochondrial membrane potential (MMP) detection**

Cells were inoculated in white 96-well plates and 96-well plates. The cell suspension concentration was 5×10⁴/ml, and 100 μl of cell suspension was added to each well. After 24 h, the cell density was approximately 50%–60%. The corresponding drugs were added for 4 h. The culture medium was aspirated, and 50 μl of TMRE staining working solution (Beyotime, China) was added. Cells were incubated in a 37°C cell culture incubator for 20 min. The MMP of cells plated in white 96-well plates was measured using a fluorescence microplate reader with a 550 nm excitation wavelength and a 575 nm emission wavelength. Cells plated in 96-well plates were observed and photographed using a fluorescence microscope.

**Chromatin immunoprecipitation (ChIP)‒qPCR**

The FASTA-formatted promoter sequences 2000 bp upstream of the transcription start sites of PDGFA and CAT were obtained from the NCBI website (https://www.ncbi.nlm.nih.gov/). The potential binding sites of AR on the promoters of PDGFA and CAT were predicted using the obtained promoter sequences in the JASPAR database (https://jaspar.elixir.no/). The primers were designed on the basis of the binding site with the highest score.

A SimpleChIP kit (Cell Signaling Technology, USA) was used to clarify the transcriptional regulatory relationships between the transcription factor AR and two genes (PDGFA and CAT). After cross-linking with 1% formaldehyde, the CRPC cell lysates were sonicated to obtain chromatin fragments. AR antibody or IgG antibody was used to immunoprecipitate DNA‒protein complexes at 4°C overnight. The immune complexes were eluted to obtain DNA fragments, which were then treated with proteinase K. PCR was used to detect promoter enrichment.

**Dual‐luciferase reporter assay**

The promoter wild-type and promoter mutant dual-luciferase plasmids were constructed according to the binding sites predicted by the JAPSAR database (https://jaspar.elixir.no/). 293T cells were seeded in 24-well plates at a cell density of approximately 50%. The dual luciferase plasmid (Genechem, China) and AR overexpression (Genechem, China) plasmid were cotransfected according to the Lipofectamine 3000 instructions (Invitrogen, USA). The activities of dual luciferase and Renilla luciferase were calculated using a dual luciferase assay kit. Renilla luciferase activity was used to normalize the results.

**Coimmunoprecipitation (****Co-IP) assay**

A Co-IP kit (Invitrogen, USA) was used to detect protein‒protein interactions. Cell lysates were prepared after cells were treated with JNJ10198409 for 12 h. A fixed proportion of the lysate was used as input, and the rest was incubated with an equal amount of specific antibody. The immunoprecipitated products were collected with A/G magnetic beads, and the immunoprecipitated and input proteins were analyzed by western blotting.

**Transmission electron microscopy (TEM)**

CRPC cells were divided into four groups, a NC group, two single-drug groups, and a combination strategy group, with three biological replicates in each group (three 10 cm cell culture dish, cell density >50%). Cells were treated with the corresponding drugs for 48 h (the drugs were prepared using serum-free culture medium at their IC50 concentrations). Then, cells were collected after glutaraldehyde fixation and centrifuged at 1000 rpm for 5 min. Then, cells were fixed with 2% osmium tetroxide, dehydrated with gradient alcohol, and embedded in resin to prepare ultrathin sections. Finally, uranyl acetate and lead citrate staining were performed, and mitochondrial morphological changes were observed by TEM (JEM-1400, Japan).

Quantitative analysis of mitochondrial morphology was performed using ImageJ 1.53 software. Mitochondrial size, circularity [4π*size / (perimeter)²], cristae density (crista number/size), and aspect ratio (major axis/minor axis) were measured [1-3]. Increased mitochondrial size, decreased cristae density, circularity and aspect ratio results close to 1 indicated mitochondrial swelling accompanied by decreased activity. In each biological replicate, the morphology of at least 50 mitochondria should be measured in different random fields of view.

**Construction of stably transfected cell lines**

RRAS knockdown lentivirus and overexpression lentivirus were purchased from Genechem (Shanghai, China). The required virus solution volume was calculated on the basis of the corresponding virus titer and MOI value of cells to be transfected, as described in the manufacturer's instructions. Transfection was performed when cells reached 40%–50% confluence in the 6-well plates. Cells were selected 72 h later using puromycin (2 μg/ml) until no significant cell death was observed. The transfection efficiency was assessed by western blotting.

**In vivo experiments with a** **zebrafish model**

Zebrafish (Hunter, Biotech., Beijing, China) of the wild-type AB strain at 3 dpf were randomly selected in 6-well plates. The concentrations of the corresponding drugs were 50 μM, 25 μM, 12.5 μM, 6.25 μM, 3.12 μM, 1.56 μM, 0.78 μM and 0.39 μM. After treatment at 35°C for 48 h, the maximum tolerance concentrations (MTCs) were determined.

Afterward, 200 CM-DiI-labeled CRPC cells were microinjected into the yolk sac of 2 dpf wild-type AB strain zebrafish. The zebrafish were cultured at 35°C until 3 dpf and randomly assigned to groups, with 10 in each group. The drug concentrations were set according to the MTCs. After being treated at 35°C for 48 h, the zebrafish were placed under a fluorescence microscope and photographed. The fluorescence intensity was analyzed using NIS-Elements D 3.20 software. No blinding was performed in the in vivo experiments.

References

1. Picard M, White K, Turnbull DM. Mitochondrial morphology, topology, and membrane interactions in skeletal muscle: a quantitative three-dimensional electron microscopy study. J Appl Physiol (1985). 2013;114(2):161-71.

2. Fry MY, Navarro PP, Hakim P, Ananda VY, Qin X, Landoni JC, et al. In situ architecture of Opa1-dependent mitochondrial cristae remodeling. Embo j. 2024;43(3):391-413.

3. Wang W, Shi Y, Qiu S, Song Y, Chen X, Zhang X, et al. TFAM signaling molecule alleviates mitochondrial damage of cerebral ischemia-reperfusion. Cell Death Discov. 2026;12(1):83.


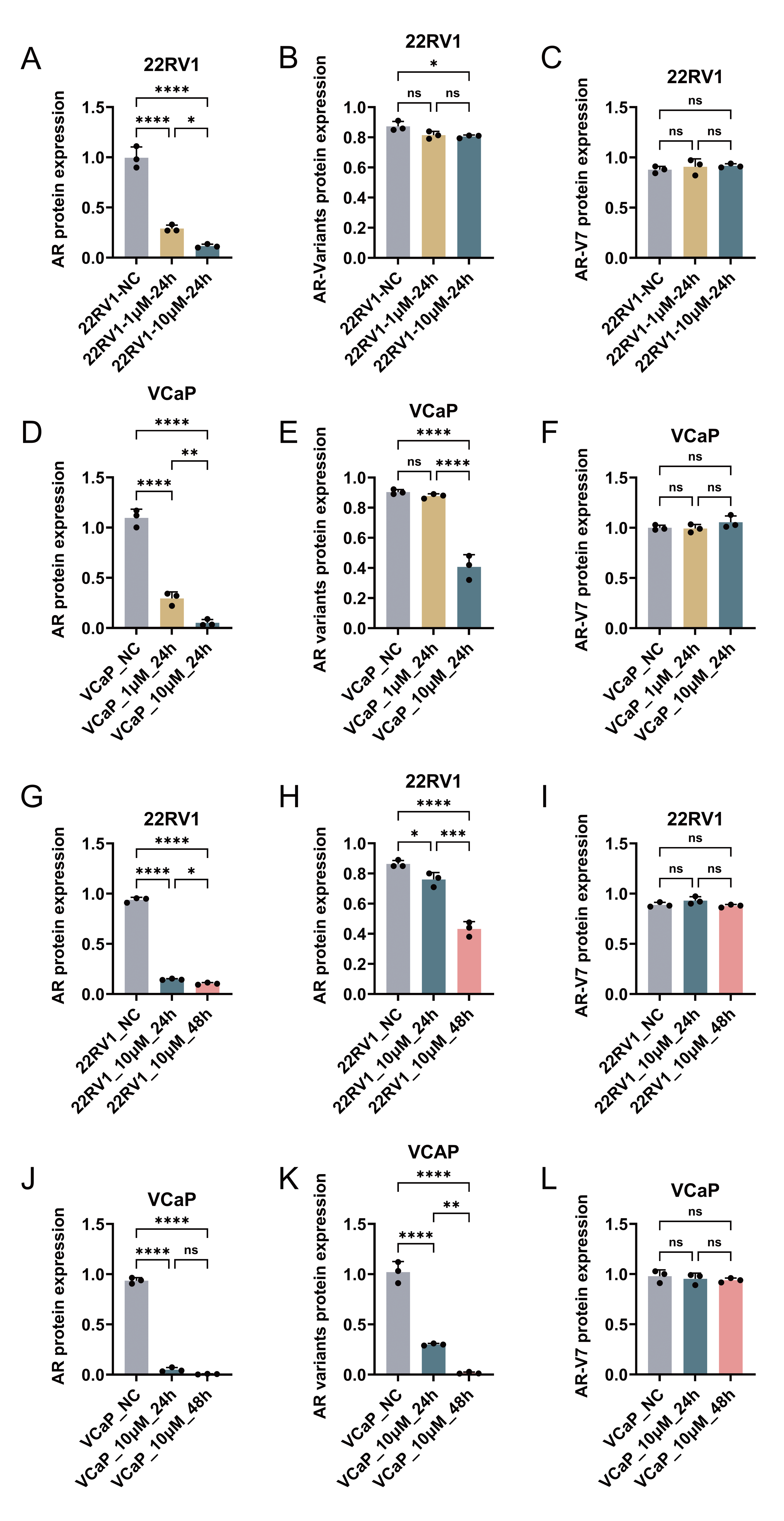


**Figure S1. Statistical analyses of the dose-time effect of ARV-110 in CRPC cells.** (A-F) The degradation efficiency of AR, AR-variants and AR-V7 in CRPC cells by ARV-110 at different concentrations and fixed time. Data were presented as the mean ± SD from three biological replicates. The Brown–Forsythe test *P* values were all > 0.05, satisfying the homogeneity of variance assumption. One-way ANOVA with Turkey multiple comparison corrections were applied. (G-L) The degradation efficiency of AR, AR-variants and AR-V7 in CRPC cells by ARV-110 at fixed concentrations and different times. Data were presented as the mean ± SD from three biological replicates. The Brown–Forsythe test *P* values were all > 0.05, satisfying the homogeneity of variance assumption. One-way ANOVA with Turkey multiple comparison corrections were applied. CRPC, castration-resistant prostate cancer; AR, androgen receptor. *, *P* < 0.05; **, *P* < 0.01; ***, *P* < 0.001; ****, *P* < 0.0001; ns, not significant.


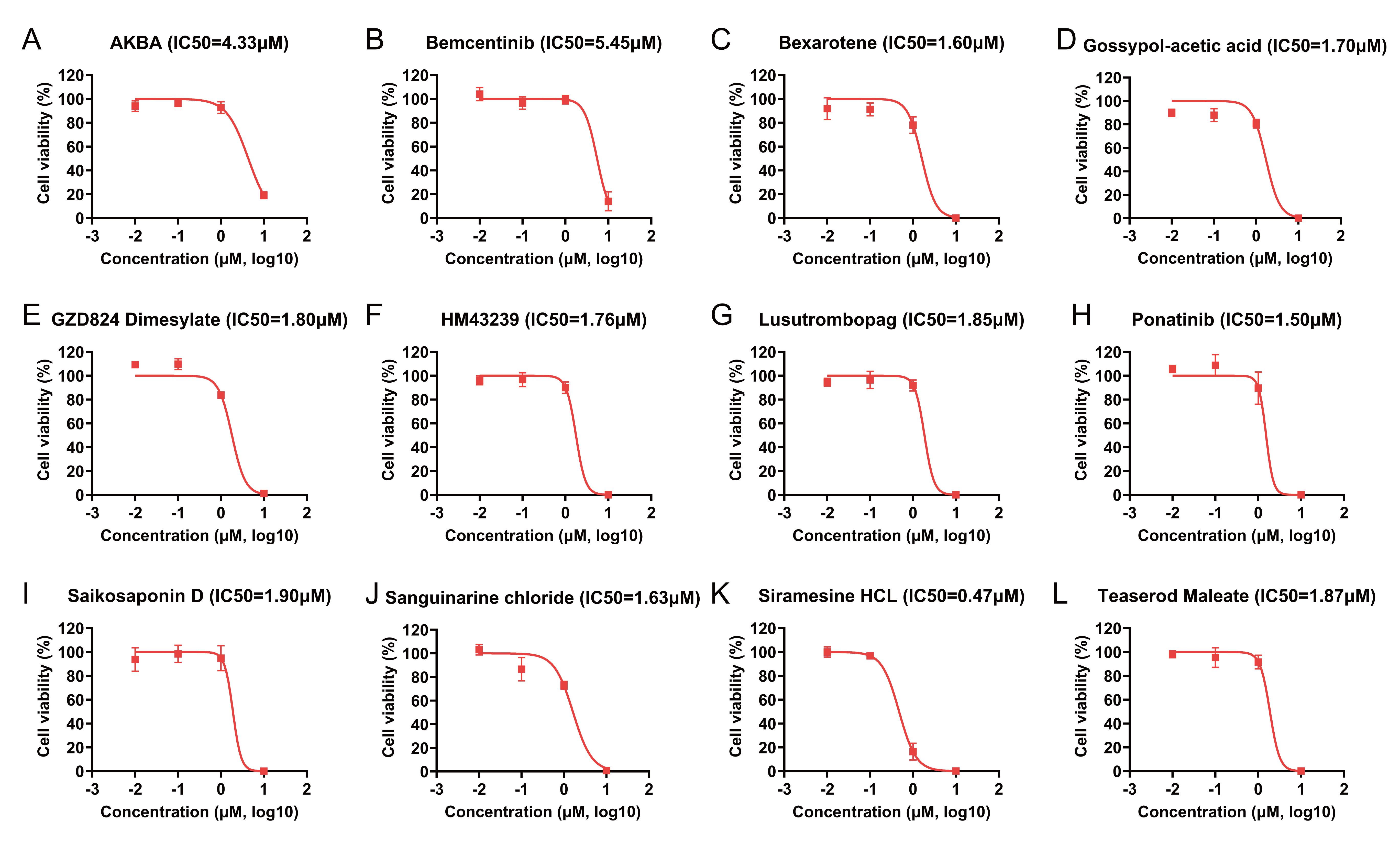
**Figure S2.** **IC50s of 12 candidate drugs in 22RV1 cell.** IC50s of 12 candidate drugs were calculated using dose-effect curves in 22RV1 cell, including (A) AKBA, (B) Bemcentinib, (C) Bexarotene, (D) Gossypol-acetic acid, (E) GZD824 Dimesylate, (F) HM43239, (G) Lusutrombopag, (H) Ponatinib, (I) Saikosaponin D, (J) Sanguinarine chloride, (K) Siramesine HCL, and (L) Teaserod Maleate. Data were presented as the mean ± SD from three biological replicates. IC50, half-maximal inhibitory concentration.


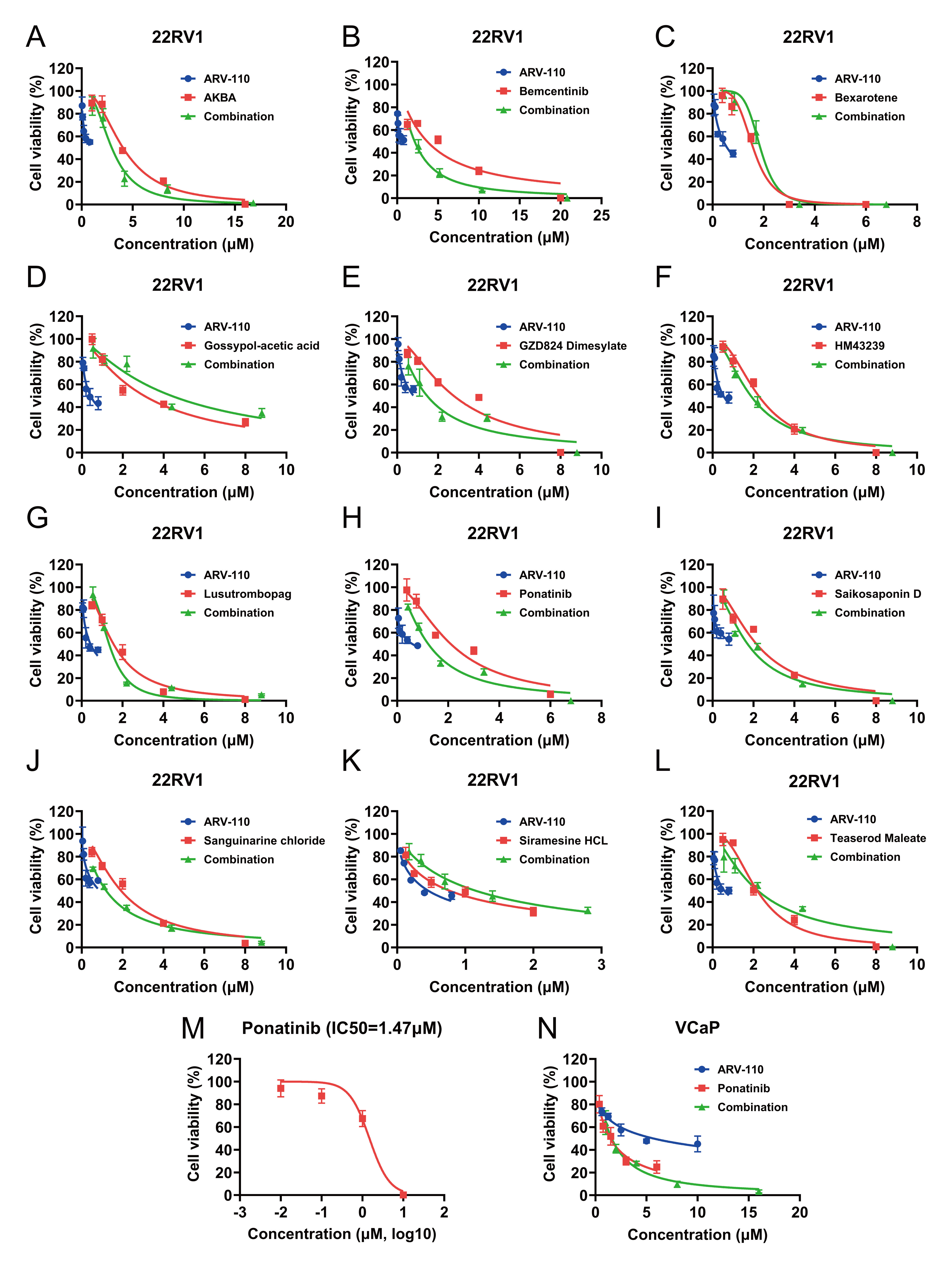
**Figure S3. Dose-effect curves were completed for calculation of CI values.** Dose-effect curves were completed for calculation of CI values in 22RV1 cell, including (A) AKBA, (B) Bemcentinib, (C) Bexarotene, (D) Gossypol-acetic acid, (E) GZD824 Dimesylate, (F) HM43239, (G) Lusutrombopag, (H) Ponatinib, (I) Saikosaponin D, (J) Sanguinarine chloride, (K) Siramesine HCL, and (L) Teaserod Maleate. (M-N) The IC50 of Ponatinib in VCaP cell was obtained and the dose-effect curve for calculating the CI value was also completed. Data were presented as the mean ± SD from three biological replicates. CI, combination index; IC50, half-maximal inhibitory concentration.


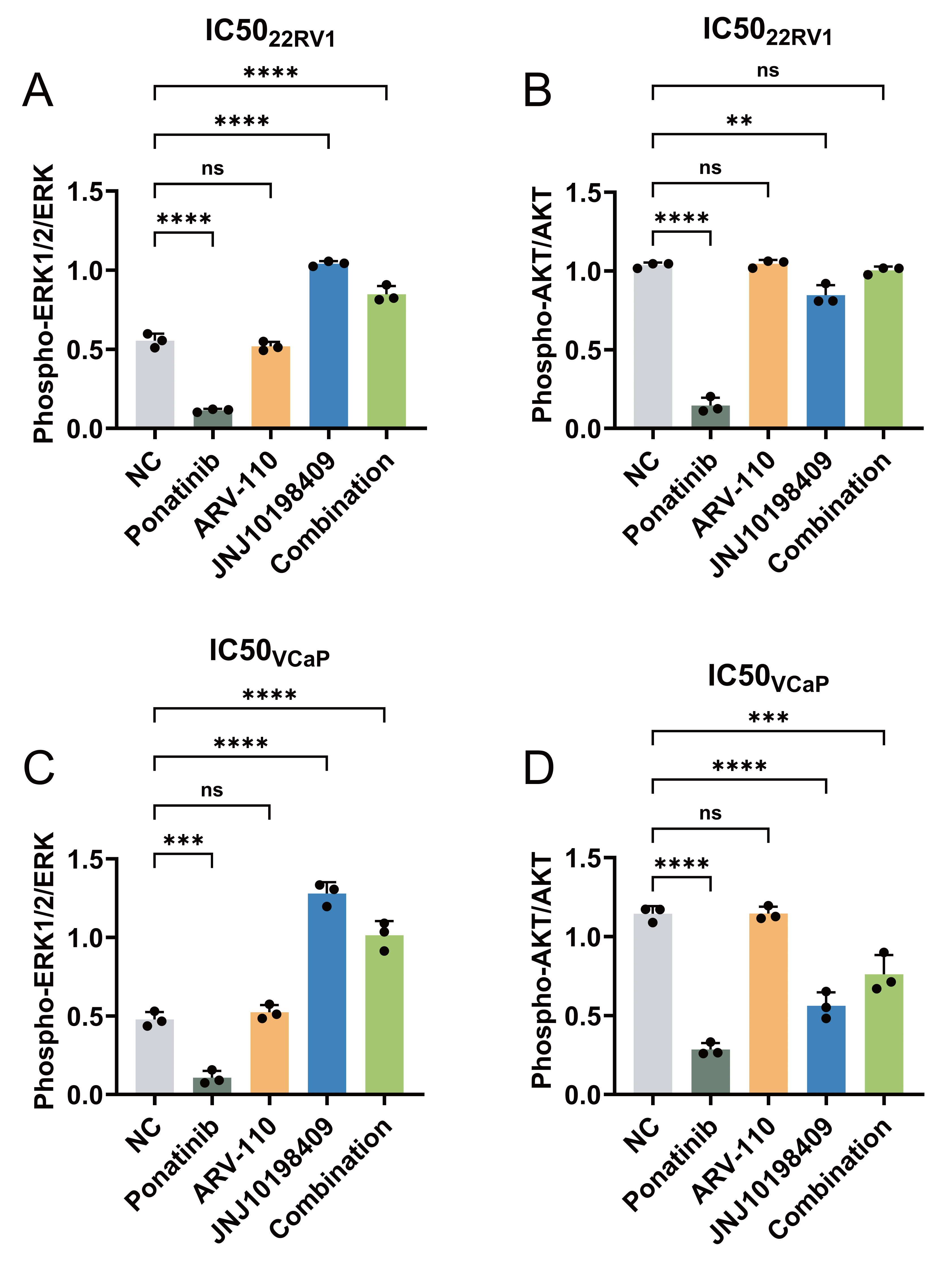
**Figure S4. Statistical analyses of** **the effect of IC50 of each drug in CRPC cells on the activity of ERK1/2 and AKT in AC16 cells.** (A-B) The effect of IC50 of each drug in 22RV1 cells on the activity of ERK1/2 and AKT in AC16 cell. Data were presented as the mean ± SD from three biological replicates. The Brown–Forsythe test *P* values were all > 0.05, satisfying the homogeneity of variance assumption. One-way ANOVA with Turkey multiple comparison corrections were applied. (C-D) The effect of IC50 of each drug in VCaP cells on the activity of ERK1/2 and AKT in AC16 cell. Data were presented as the mean ± SD from three biological replicates. The Brown–Forsythe test *P* values were all > 0.05, satisfying the homogeneity of variance assumption. One-way ANOVA with Turkey multiple comparison corrections were applied. CRPC, castration-resistant prostate cancer; ERK, extracellular regulated protein kinase; IC50, half-maximal inhibitory concentration. **, *P* < 0.01; ***, *P* < 0.001; ****, *P* < 0.0001; ns, not significant.


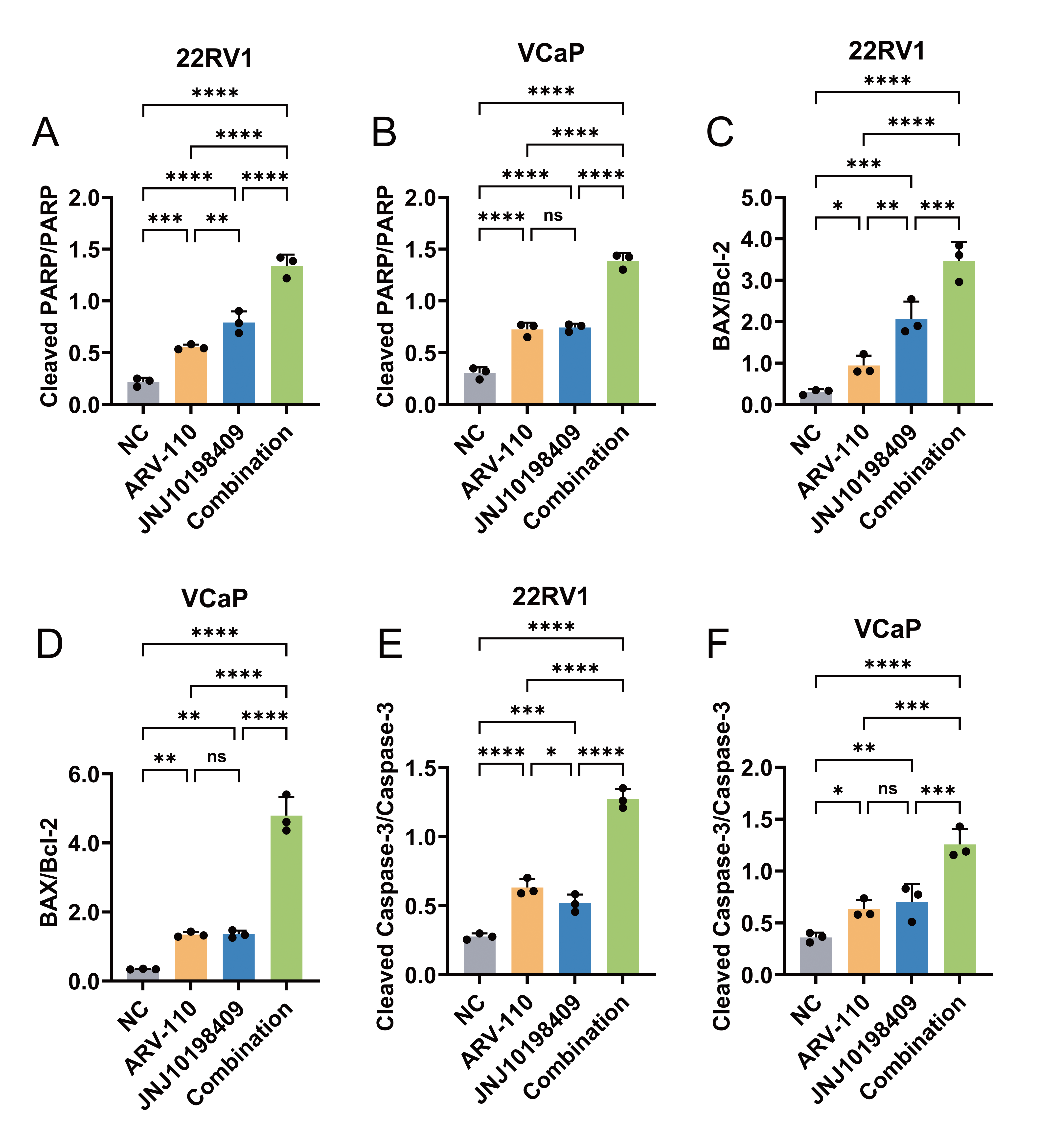
**Figure S5. Statistical analyses of the effects of ARV-110 and JNJ10198409 alone and in combination on** **apoptosis-related proteins.** Both ARV-110 and JNJ10198409 could increase the levels of apoptosis-related proteins. The combination strategy further increased the levels of apoptosis-related proteins, including (A-B) cleaved PARP/PARP, (C-D) BAX/Bcl-2 and (E-F) cleaved Caspase-3/Caspase-3. Data were presented as the mean ± SD from three biological replicates. The Brown–Forsythe test *P* values were all > 0.05, satisfying the homogeneity of variance assumption. One-way ANOVA with Turkey multiple comparison corrections were applied. PARP, poly ADP ribose polymerase; BAX, Bcl-2 associated X protein; Bcl-2, B cell lymphoma 2; Caspase-3, cysteinyl aspartate specific protease-3. *, *P* < 0.05; **, *P* < 0.01; ***, *P* < 0.001; ****, *P* < 0.0001; ns, not significant.


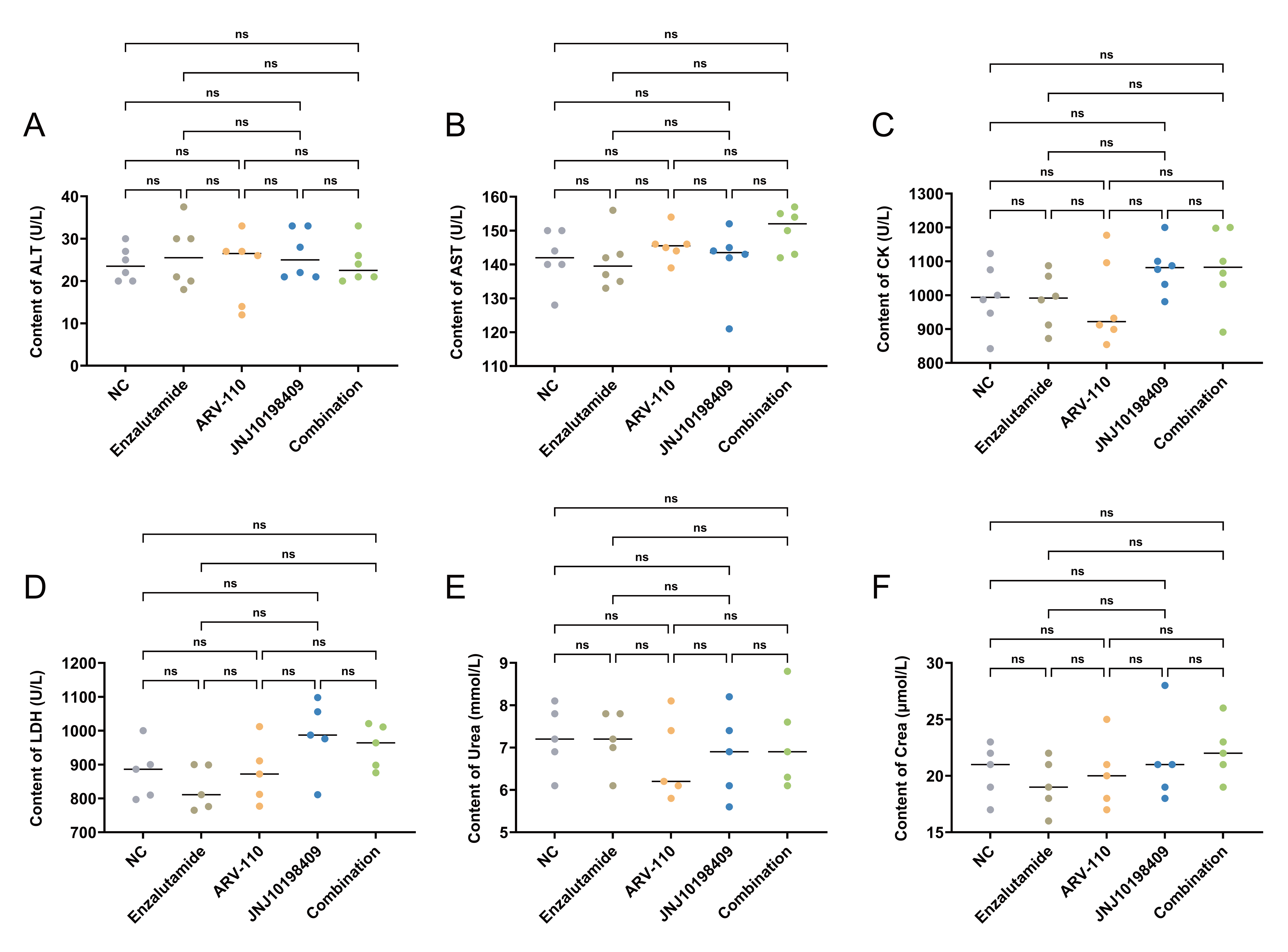
**Figure S6. The effect of each drug on the biochemical indices of the nude mice in the tumor formation experiment.** No statistical differences were observed in the biochemical indices of (A-B, ALT and AST) liver function, (C-D, CK and LDH) cardiac function, and (E-F, Urea and Crea) renal function of the nude mice (n=5/group). Kruskal Wallis tests with Dunn multiple comparison corrections were applied. ALT, alanine aminotransferase; AST, aspartate aminotransferase; CK, creatine kinase; LDH, lactate dehydrogenase; Crea, creatinine; ns, not significant.


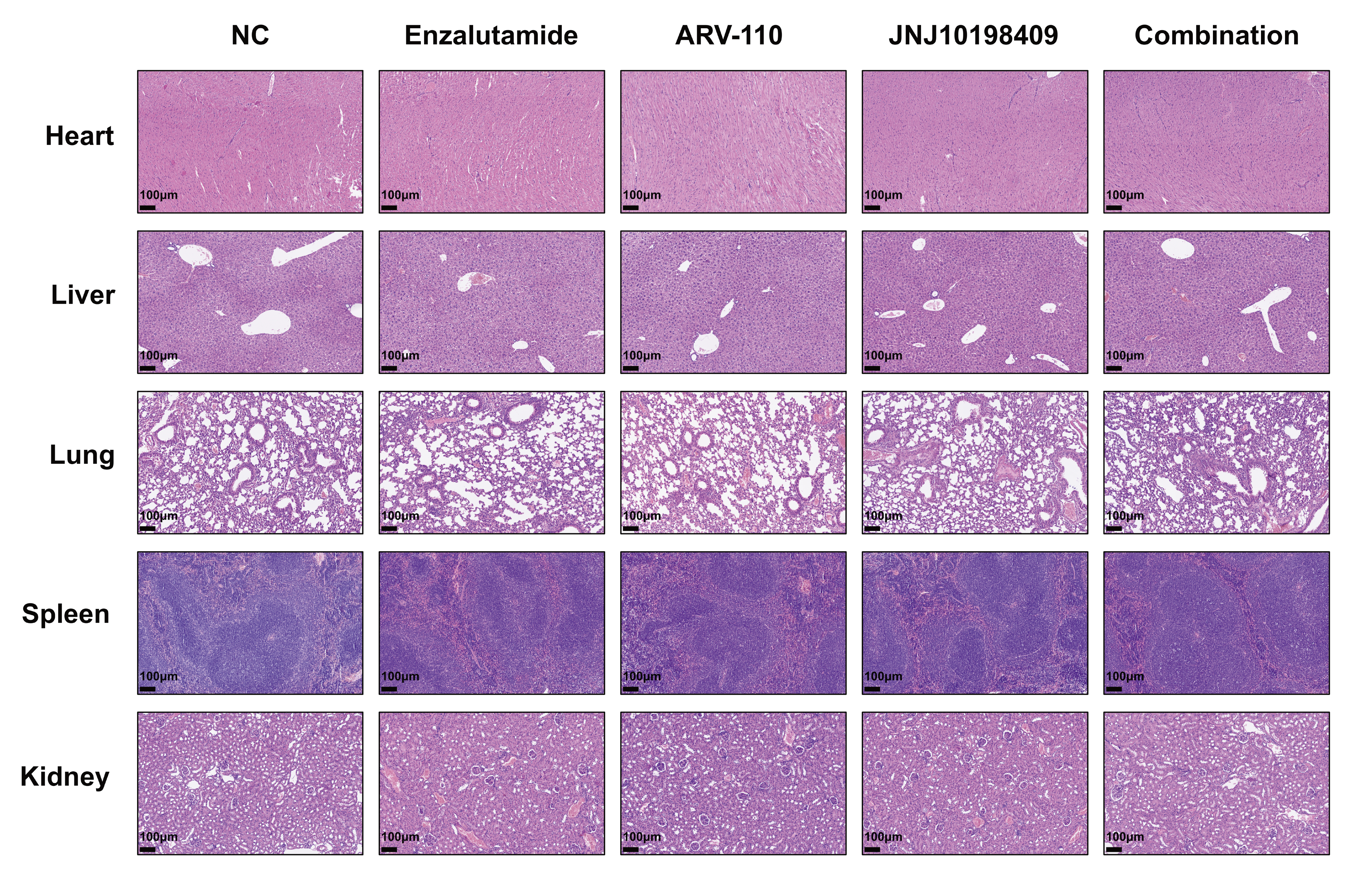
**Figure S7. Histopathological changes in various drug regimens in nude mice.** No obvious histological abnormalities were observed in the organ sections of the nude mice, including heart, liver, lungs, spleen and kidneys (n=5/group).


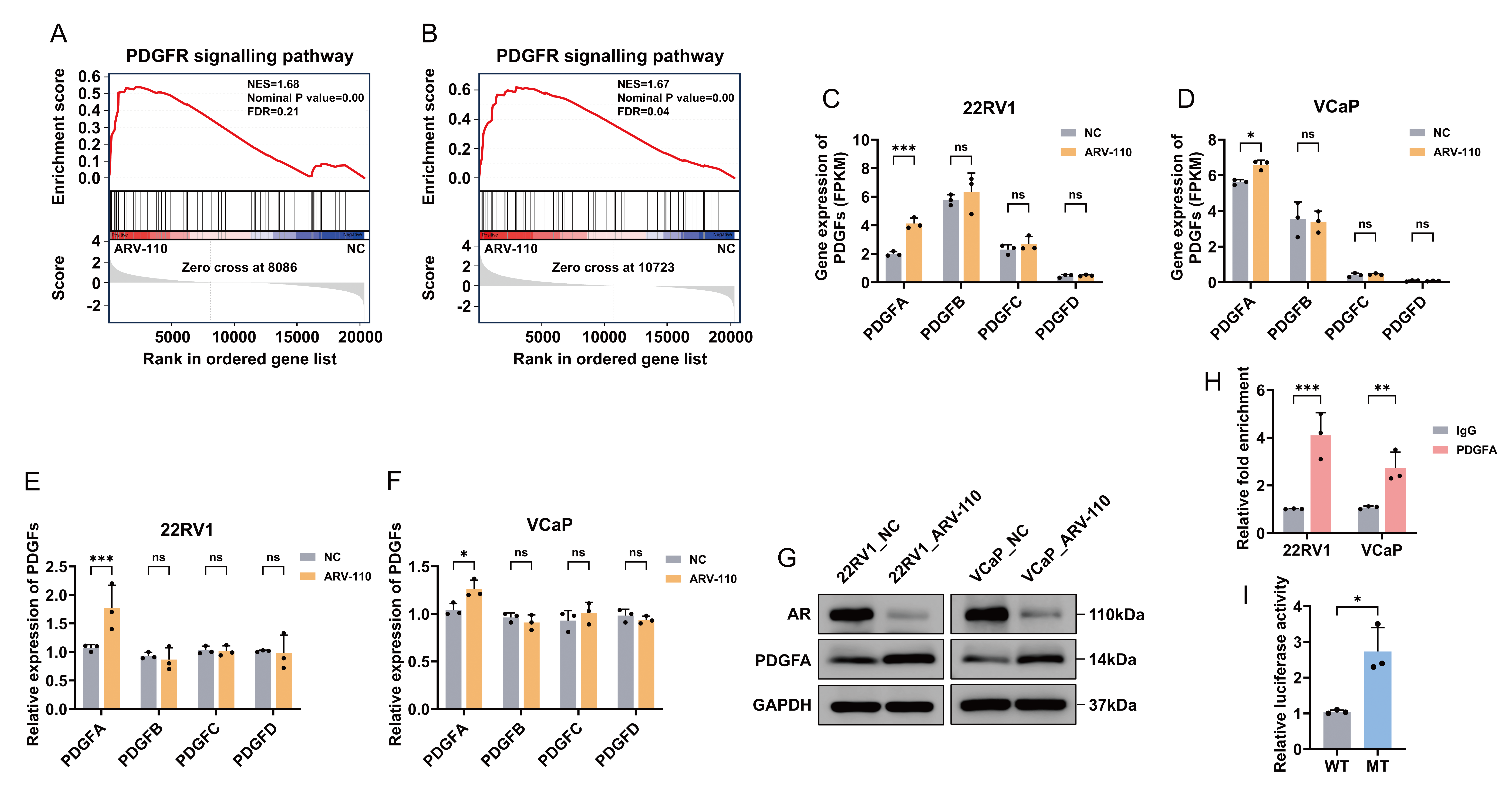
**Figure S8. AR could negatively regulate the transcription of PDGFA.** (A-B) Based on the intergroup GSEA results of transcriptome sequencing data of the NC group and the ARV-110 group, it was shown that compared with the NC group, the ARV-110 group that produced AR degradation effect had activation of the PDGFR signaling pathway. The results of (C-D) transcriptome sequencing data, (E-F) qRT-PCR and (G) western blotting all confirmed that only PDGFA in CRPC cells treated with ARV-110 increased significantly. Data were presented as the mean ± SD from three biological replicates. The Brown–Forsythe test *P* values were all > 0.05, satisfying the homogeneity of variance assumption. One-way ANOVA with Turkey multiple comparison corrections were applied. (H-I) ChIP-qPCR and dual luciferase reporter assays were used to verify that AR could bind to the PDGFA promoter region and play a negative transcriptional regulatory function. Data were presented as the mean ± SD from three biological replicates. The F test *P* values were all > 0.05, satisfying the homogeneity of variance assumption. The unpaired T-tests were applied. AR, androgen receptor; PDGFA, platelet-derived growth factor A; PDGFR, platelet-derived growth factor receptor; CRPC, castration-resistant prostate cancer. *, *P* < 0.05; **, *P* < 0.01; ***, *P* < 0.001; ns, not significant.


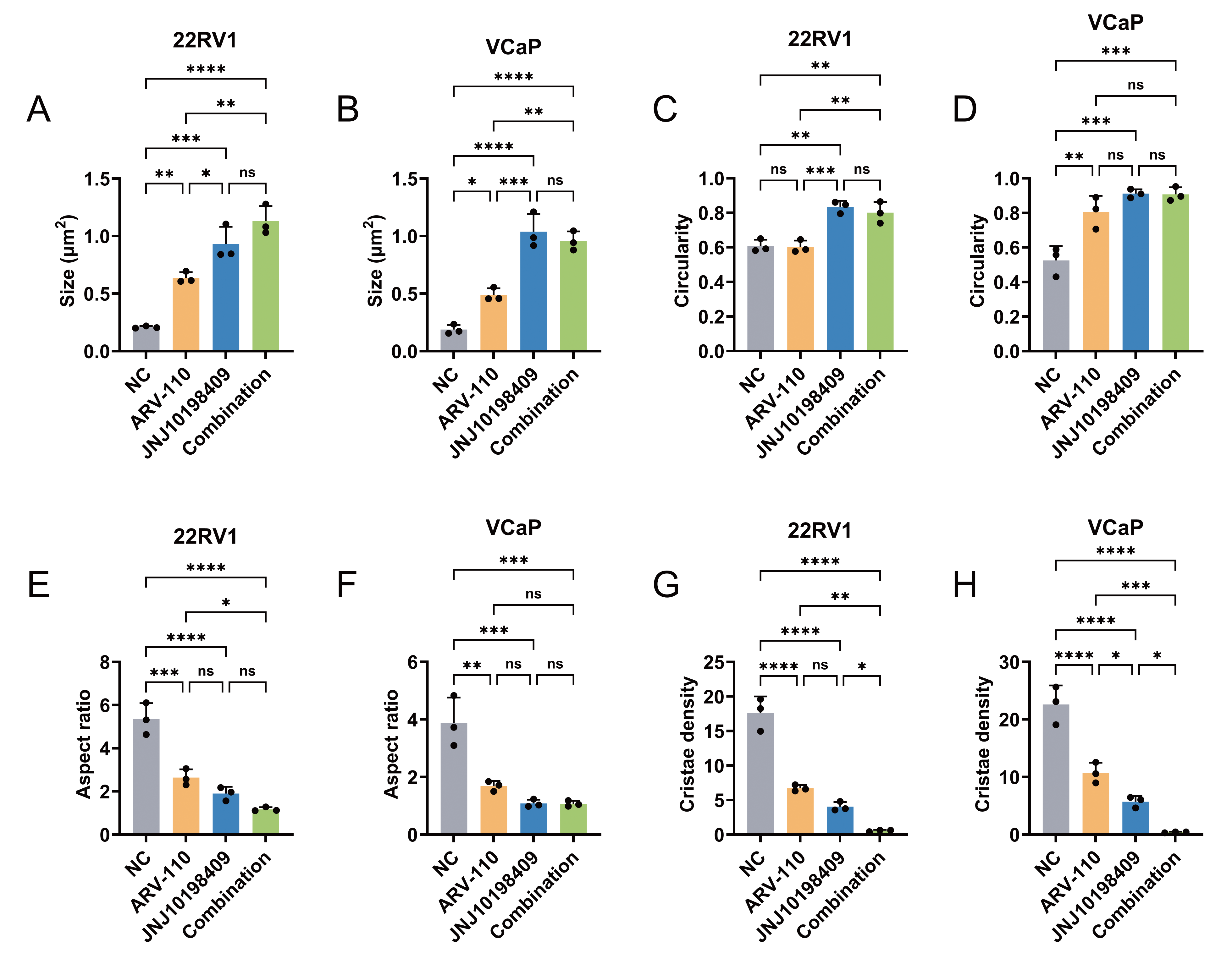


**Figure S9. Statistical analyses of mitochondrial morphological changes induced by combination strategy.** Quantitative statistical analyses of mitochondrial morphology were performed, including (A-B) size, (C-D) circularity, (E-F) aspect ratio, and (G-H) cristae density. The results revealed that the combined strategy caused severe damage to mitochondria in CRPC cells, with significant mitochondrial swelling and almost complete disappearance of cristae. Data were presented as the mean ± SD from three biological replicates. The Brown–Forsythe test *P* values were all > 0.05, satisfying the homogeneity of variance assumption. One-way ANOVA with Turkey multiple comparison corrections were applied. CRPC, castration-resistant prostate cancer. *, *P* < 0.05; **, *P* < 0.01; ***, *P* < 0.001; ****, *P* < 0.0001; ns, not significant.


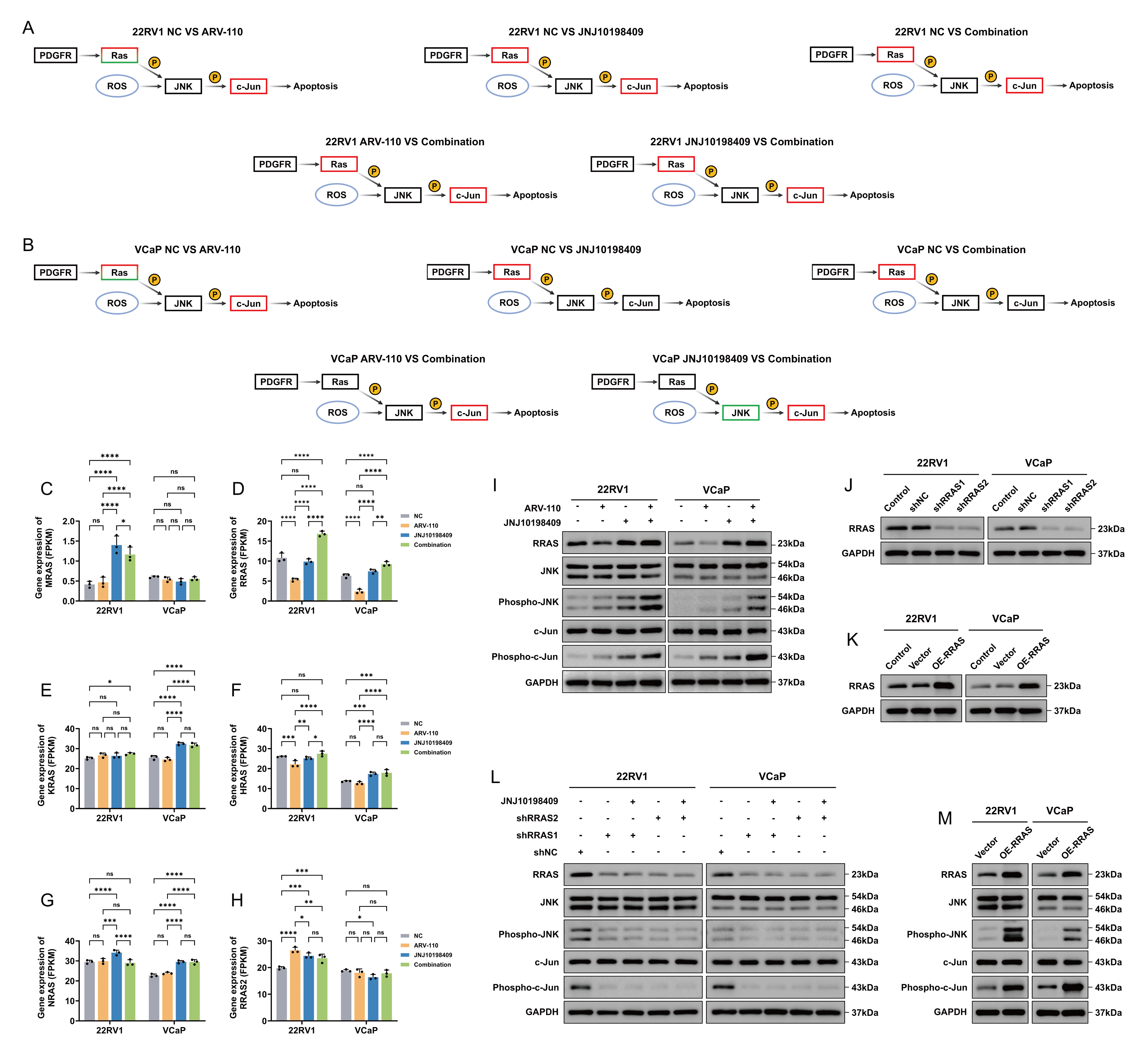
**Figure S10. The combined strategy induced CRPC cell apoptosis by increasing RRAS expression and activating the JNK signaling pathway.** (A-B) The results of the differential expression analyses of related genes in the JNK signaling pathway based on transcriptome sequencing data showed that most of the comparisons between the ARV-110 and JNJ10198409 alone and combined use groups showed changes in the transcriptional level of the Ras family. Transcriptome sequencing used three biological replicates during the sample preparation stage. Red rectangles represented increased gene expression; red-green rectangles represented increased expression of certain genes and decreased expression of others; black rectangles represented no difference in gene expression; blue circles represented hub upstream factors. (C-H) Compared with other members of the Ras family, the gene expression level of RRAS showed significant changes when ARV-110 and JNJ10198409 were used alone or in combination. Data were presented as the mean ± SD from three biological replicates. The Brown–Forsythe test *P* values were all > 0.05, satisfying the homogeneity of variance assumption. One-way ANOVA with Turkey multiple comparison corrections were applied. (I) ARV-110 alone could reduce RRAS, while slightly increasing phospho-JNK and phospho-c-Jun. JNJ10198409 alone could inhibit PDGFR activity and upregulate RRAS expression as well as increase phospho-JNK and phospho-c-Jun. Combination strategy significantly increased RRAS expression and JNK signaling pathway activation levels. (J-K) Stable CRPC cell lines with RRAS knockdown and overexpression were constructed. (L) After RRAS knockdown, the JNK signaling pathway was suppressed and was not restored after the addition of JNJ10198409. (M) However, after RRAS overexpression, the JNK signaling pathway was significantly activated. CRPC, castration-resistant prostate cancer; JNK, c-Jun N-terminal kinase; PDGFR, platelet-derived growth factor receptor. *, *P* < 0.05; **, *P* < 0.01; ***, *P* < 0.001; ****, *P* < 0.0001; ns, not significant.


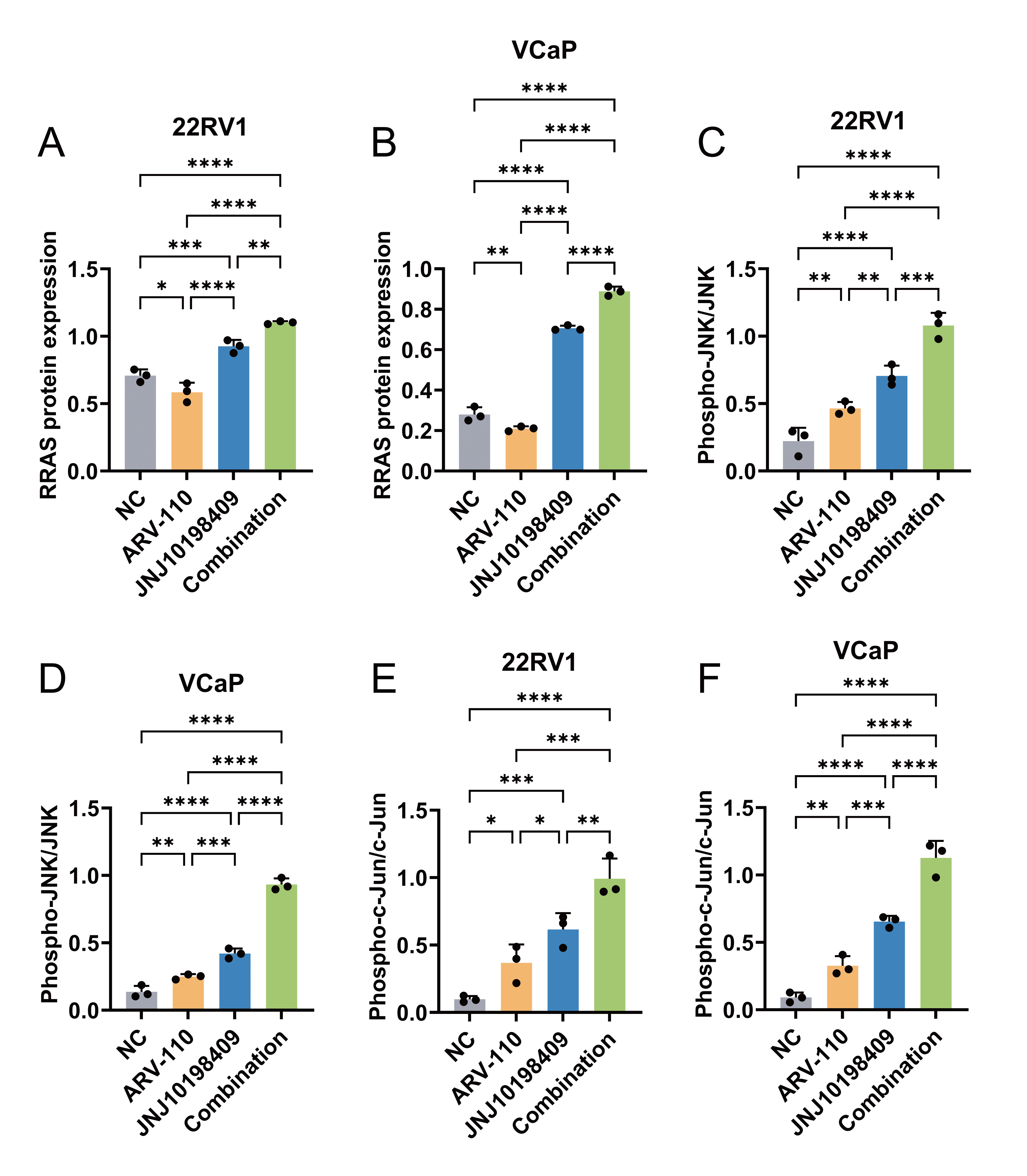
**Figure S11. Statistical analyses of the effects of ARV-110 and JNJ10198409 alone and in combination on** **JNK signaling pathway related proteins.** The expressions of JNK signaling pathway related proteins were significantly changed, including (A-B) RRAS, (C-D) phospho-JNK and (E-F) phospho-c-Jun. Data were presented as the mean ± SD from three biological replicates. The Brown–Forsythe test *P* values were all > 0.05, satisfying the homogeneity of variance assumption. One-way ANOVA with Turkey multiple comparison corrections were applied. JNK, c-Jun N-terminal kinase. *, *P* < 0.05; **, *P* < 0.01; ***, *P* < 0.001; ****, *P* < 0.0001.


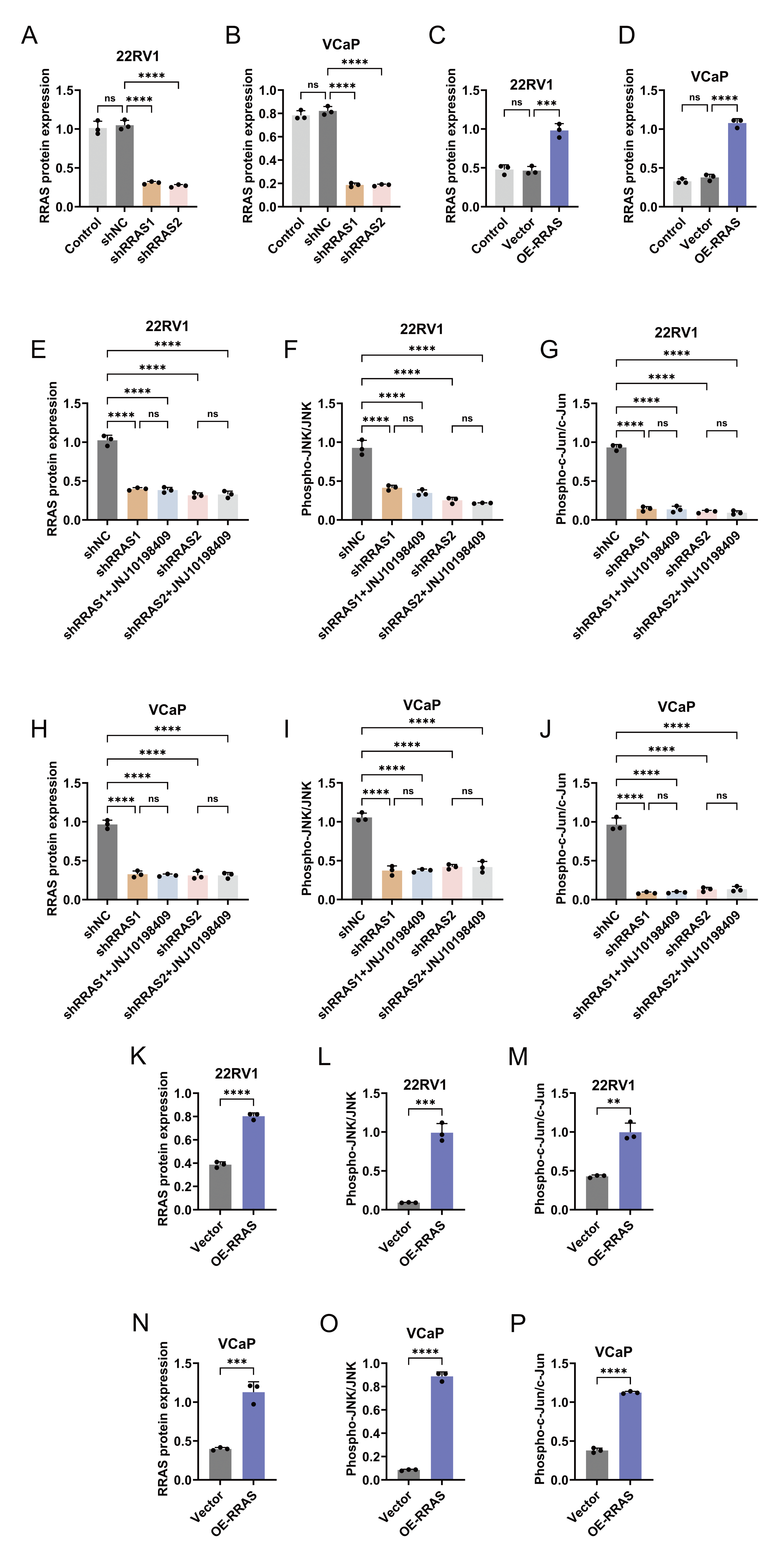


**Figure S12 Statistical analyses of the effects of RRAS knockdown or overexpression on the JNK signaling pathway.** (A-B) RRAS expression was significantly decreased in CRPC cells stably transfected with knockdown lentivirus. Data were presented as the mean ± SD from three biological replicates. The Brown–Forsythe test *P* values were all > 0.05, satisfying the homogeneity of variance assumption. One-way ANOVA with Turkey multiple comparison corrections were applied. (C-D) RRAS expression was significantly increased in CRPC cells transfected with RRAS overexpressing lentivirus. Data were presented as the mean ± SD from three biological replicates. The Brown–Forsythe test *P* values were all > 0.05, satisfying the homogeneity of variance assumption. One-way ANOVA with Turkey multiple comparison corrections were applied. (E-J) After RRAS knockdown, the JNK signaling pathway was suppressed and was not restored after the addition of JNJ10198409. Data were presented as the mean ± SD from three biological replicates. The Brown–Forsythe test *P* values were all > 0.05, satisfying the homogeneity of variance assumption. One-way ANOVA with Turkey multiple comparison corrections were applied. (K-P) After RRAS overexpression, the JNK signaling pathway was significantly activated. Data were presented as the mean ± SD from three biological replicates. The F test *P* values were all > 0.05, satisfying the homogeneity of variance assumption. The unpaired T-tests were applied. CRPC, castration-resistant prostate cancer. **, *P* < 0.01; ***, *P* < 0.001; ****, *P* < 0.0001; ns, not significant.


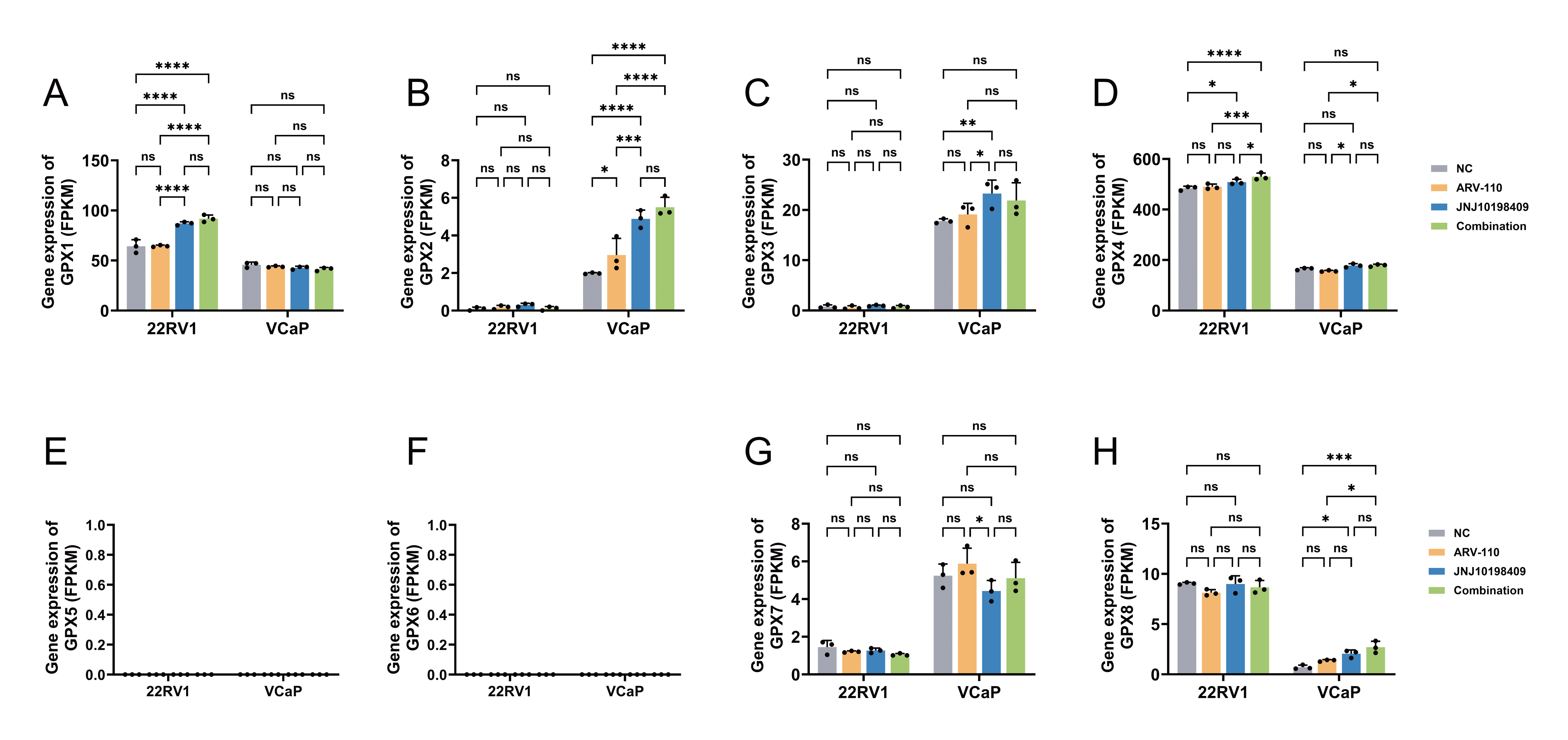
**Figure S13. Effects of ARV-110 and JNJ10198409 alone and in combination on the expression of GPX family.** Based on transcriptome sequencing data, the expressions of GPX family were evaluated, including (A) GPX1, (B) GPX2, (C) GPX3, (D) GPX4, (E) GPX5, (F) GPX6, (G) GPX7 and (H) GPX8. Data were presented as the mean ± SD from three biological replicates. The Brown–Forsythe test *P* values were all > 0.05, satisfying the homogeneity of variance assumption. One-way ANOVA with Turkey multiple comparison corrections were applied. GPX, glutathione peroxidase. *, *P* < 0.05; **, *P* < 0.01; ***, *P* < 0.001; ****, *P* < 0.0001; ns, not significant.


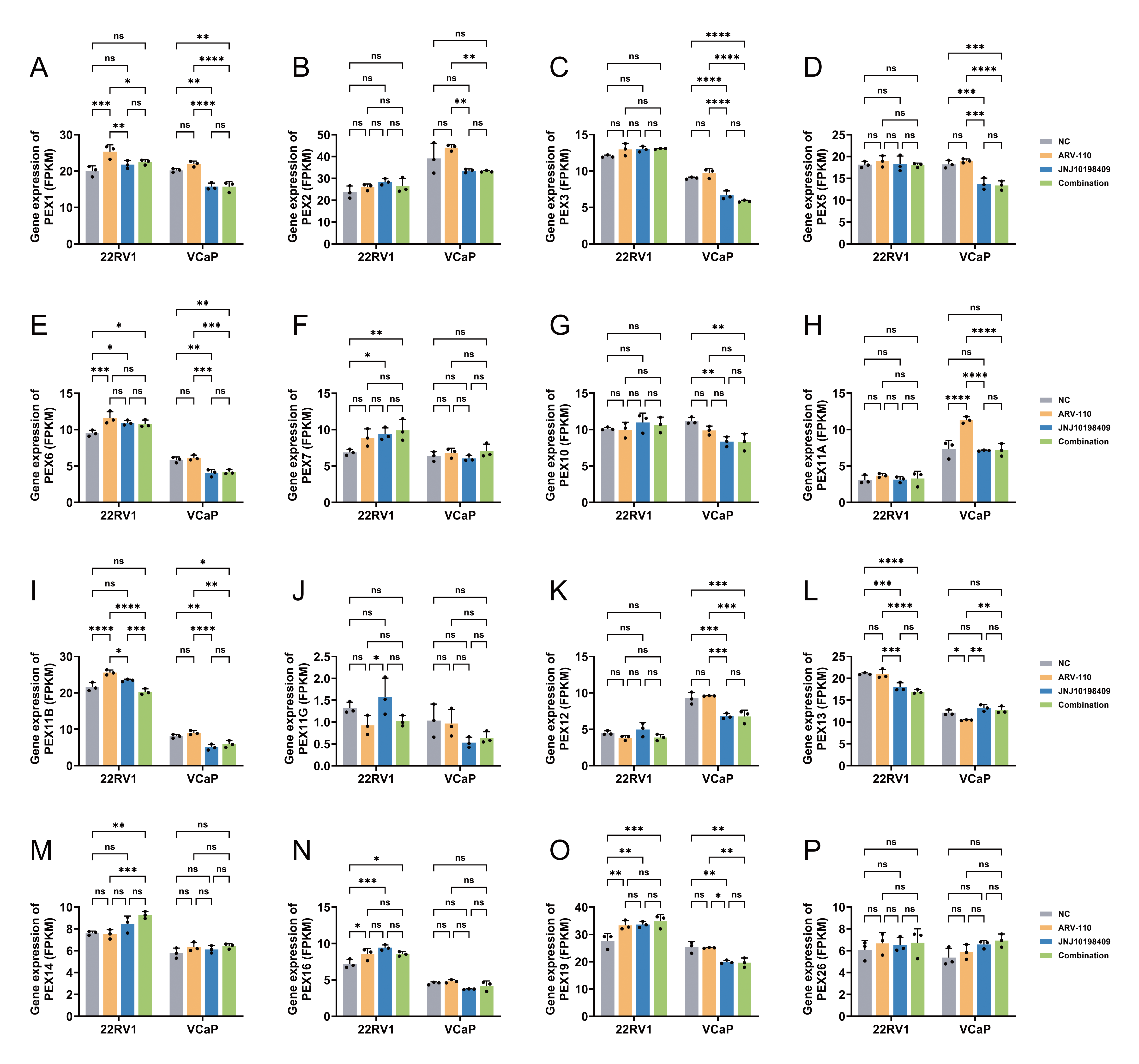
**Figure S14. Effects of ARV-110 and JNJ10198409 alone and in combination on the expression of PEX family.** Based on transcriptome sequencing data, the expressions of PEX family were evaluated, including (A) PEX1, (B) PEX2, (C) PEX3, (D) PEX5, (E) PEX6, (F) PEX7, (G) PEX10, (H) PEX11A, (I) PEX11B, (J) PEX11G, (K) PEX12, (L) PEX13, (M) PEX14, (N) PEX16, (O) PEX19 and (P) PEX26. Data were presented as the mean ± SD from three biological replicates. The Brown–Forsythe test *P* values were all > 0.05, satisfying the homogeneity of variance assumption. One-way ANOVA with Turkey multiple comparison corrections were applied. PEX, peroxisomal biogenesis factor. *, *P* < 0.05; **, *P* < 0.01; ***, *P* < 0.001; ****, *P* < 0.0001; ns, not significant.


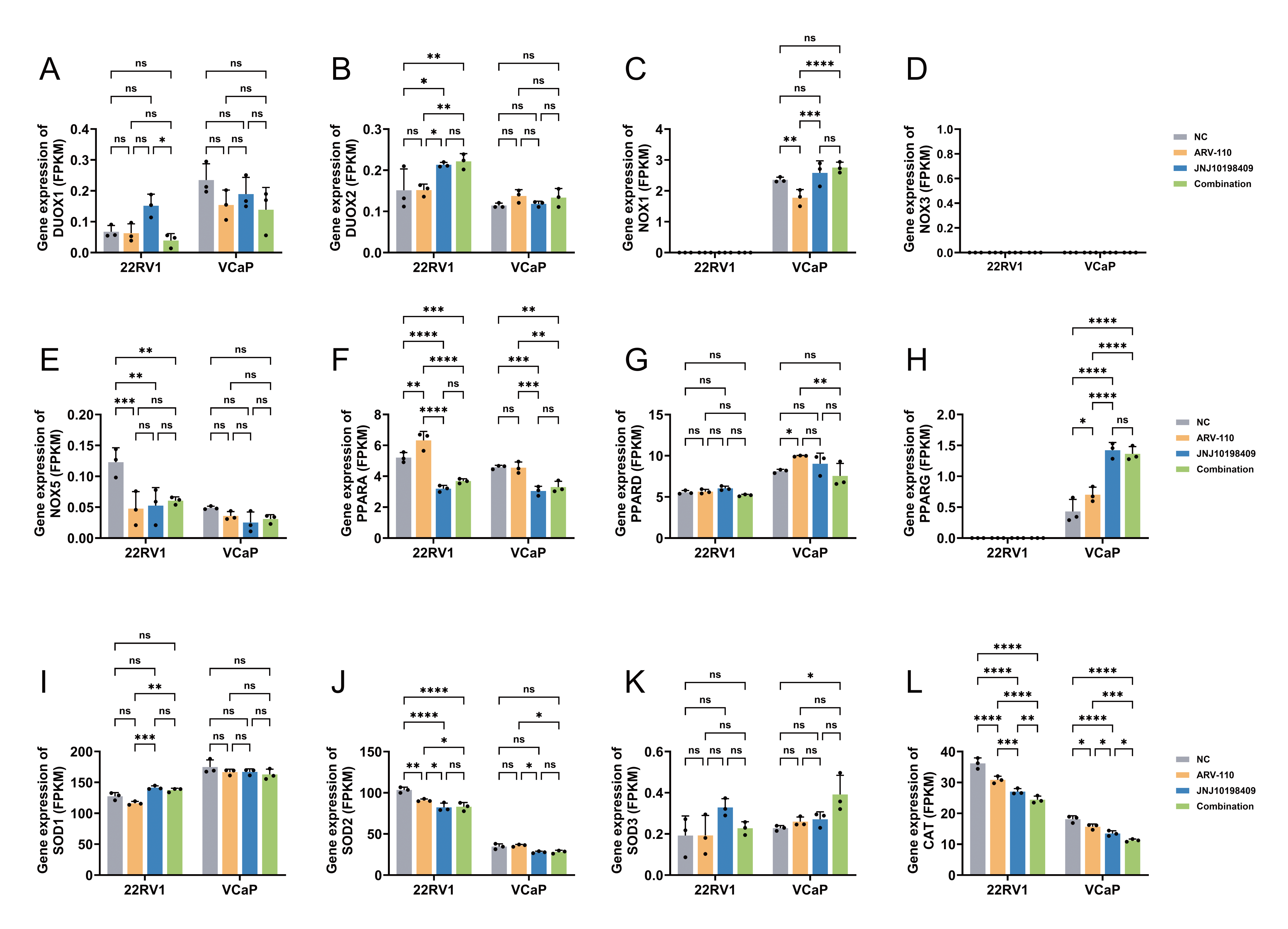
**Figure S15. Effects of ARV-110 and JNJ10198409 alone and in combination on the expression of** **NOX family, PPAR family, SOD family and CAT.** Based on transcriptome sequencing data, the expressions of NOX family, PPAR family, SOD family and CAT were evaluated, including (A) DUOX1, (B) DUOX2, (C) NOX1, (D) NOX3, (E) NOX5, (F) PPARA, (G) PPARD, (H) PPARG, (I) SOD1, (J) SOD2, (K) SOD3 and (L) CAT. Data were presented as the mean ± SD from three biological replicates. The Brown–Forsythe test *P* values were all > 0.05, satisfying the homogeneity of variance assumption. One-way ANOVA with Turkey multiple comparison corrections were applied. NOX, NADPH oxidase; PPAR, peroxisome proliferator-activated receptor; SOD, superoxide dismutase; CAT, catalase. *, *P* < 0.05; **, *P* < 0.01; ***, *P* < 0.001; ****, *P* < 0.0001; ns, not significant.


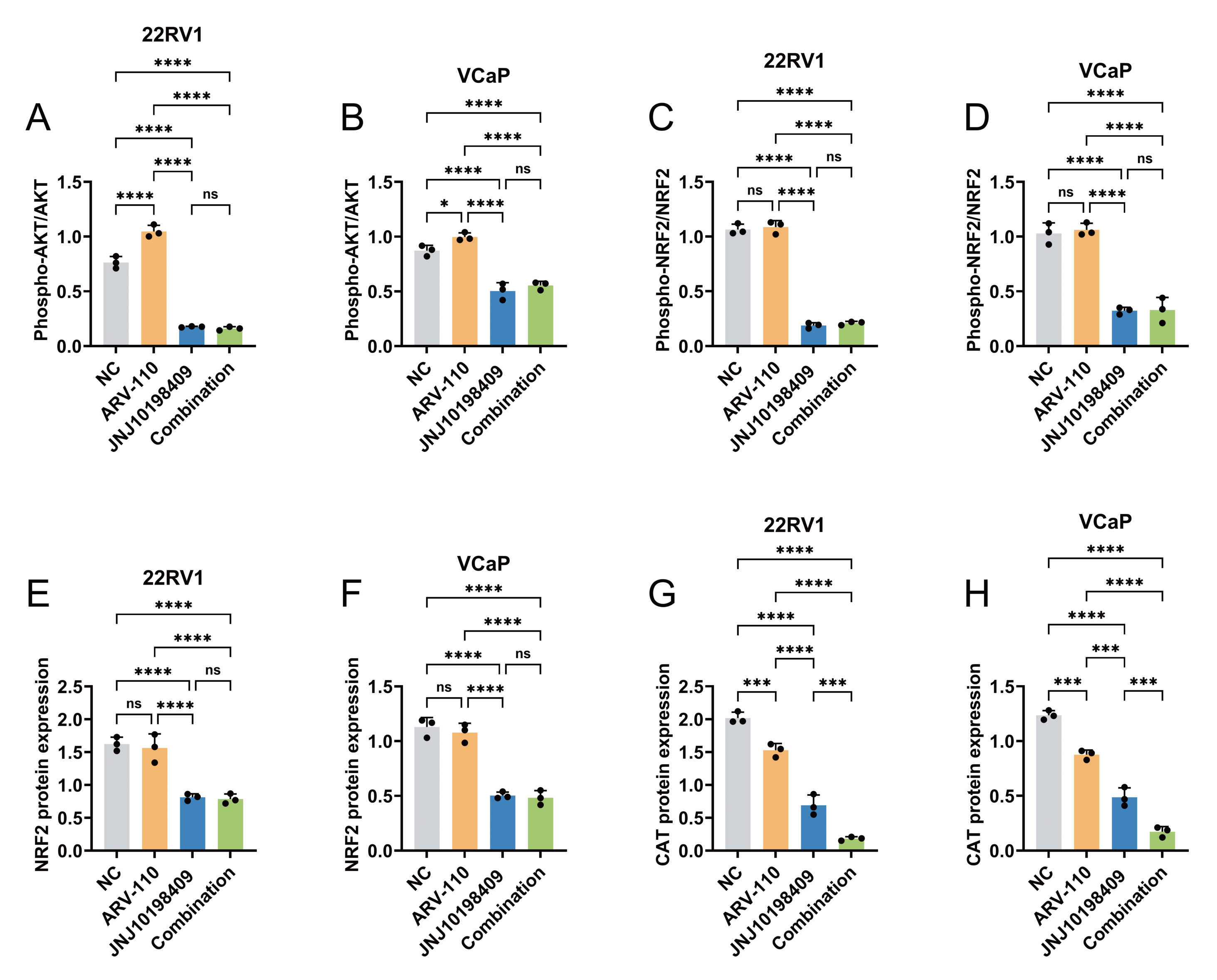
**Figure S16. Statistical analyses of the effects of ARV-110 and JNJ10198409 alone and in combination on NRF2 related proteins.** The expressions of NRF2 related proteins were significantly changed, including (A-B) phospho-AKT, (C-D) phospho-NRF2, (E-F) NRF2 and (G-H) CAT. Data were presented as the mean ± SD from three biological replicates. The Brown–Forsythe test *P* values were all > 0.05, satisfying the homogeneity of variance assumption. One-way ANOVA with Turkey multiple comparison corrections were applied. NRF2, nuclear factor-erythroid 2-related factor 2; CAT, catalase. *, *P* < 0.05; ***, *P* < 0.001; ****, *P* < 0.0001; ns, not significant.


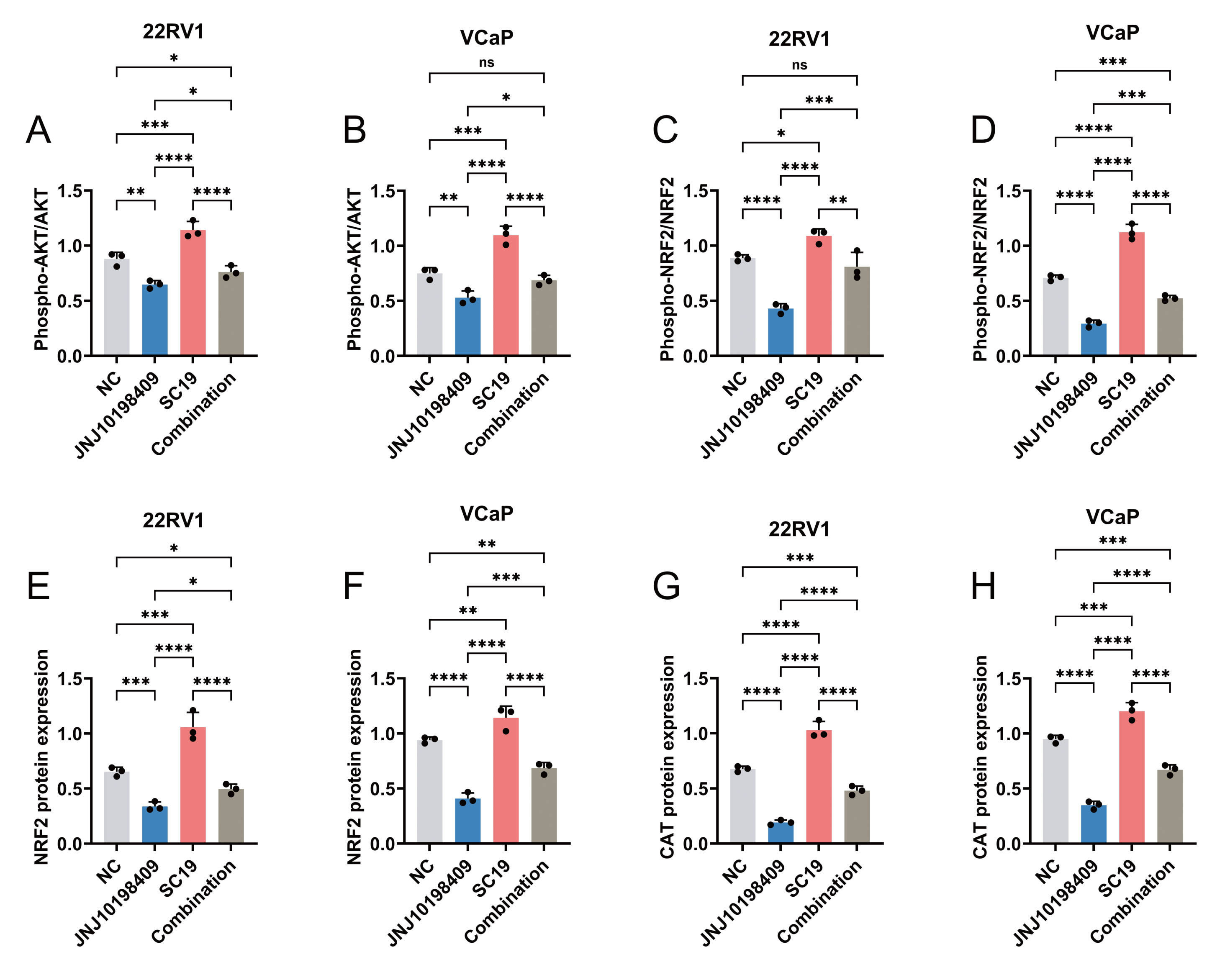
**Figure S17. Statistical analyses of the effects of JNJ10198409 and SC19 alone and in combination on NRF2 related proteins.** The expressions of NRF2 related proteins were significantly changed, including (A-B) phospho-AKT, (C-D) phospho-NRF2, (E-F) NRF2 and (G-H) CAT. Data were presented as the mean ± SD from three biological replicates. The Brown–Forsythe test *P* values were all > 0.05, satisfying the homogeneity of variance assumption. One-way ANOVA with Turkey multiple comparison corrections were applied. NRF2, nuclear factor-erythroid 2-related factor 2; CAT, catalase. *, *P* < 0.05; **, *P* < 0.01; ***, *P* < 0.001; ****, *P* < 0.0001; ns, not significant.


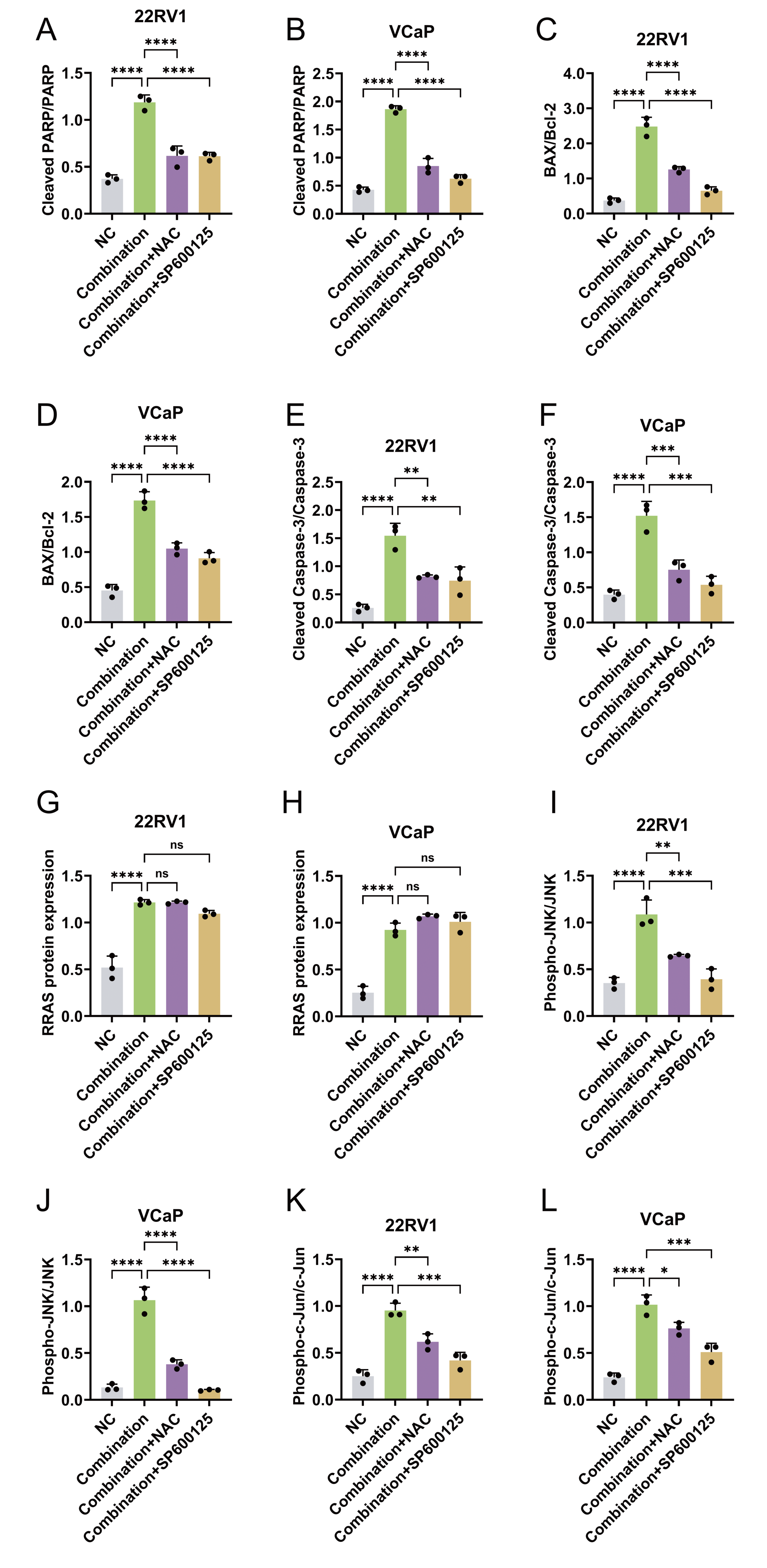


**Figure S18. Statistical analyses of the effects of combination of ARV-110 and JNJ10198409 with the addition of NAC and SP600125 on apoptosis-related proteins and JNK signaling pathway related proteins.** (A-F) Combination of ARV-110 and JNJ10198409 with the addition of NAC and SP600125 could reverse the activation of apoptosis-related proteins, including cleaved PARP/PARP, BAX/Bcl-2 and cleaved Caspase-3/Caspase-3. Data were presented as the mean ± SD from three biological replicates. The Brown–Forsythe test *P* values were all > 0.05, satisfying the homogeneity of variance assumption. One-way ANOVA with Turkey multiple comparison corrections were applied. (G-H) As for JNK signaling pathway-related proteins, RRAS expression remained unchanged. Data were presented as the mean ± SD from three biological replicates. The Brown–Forsythe test *P* values were all > 0.05, satisfying the homogeneity of variance assumption. One-way ANOVA with Turkey multiple comparison corrections were applied. (I-L) While the expressions of other proteins were reversed, including phospho-JNK and phospho-c-Jun. Data were presented as the mean ± SD from three biological replicates. The Brown–Forsythe test *P* values were all > 0.05, satisfying the homogeneity of variance assumption. One-way ANOVA with Turkey multiple comparison corrections were applied. NAC, N-acetylcysteine; JNK, c-Jun N-terminal kinase; PARP, poly ADP ribose polymerase; BAX, Bcl-2 associated X protein; Bcl-2, B cell lymphoma 2; Caspase-3, cysteinyl aspartate specific protease-3. *, *P* < 0.05; **, *P* < 0.01; ***, *P* < 0.001; ****, *P* < 0.0001; ns, not significant.


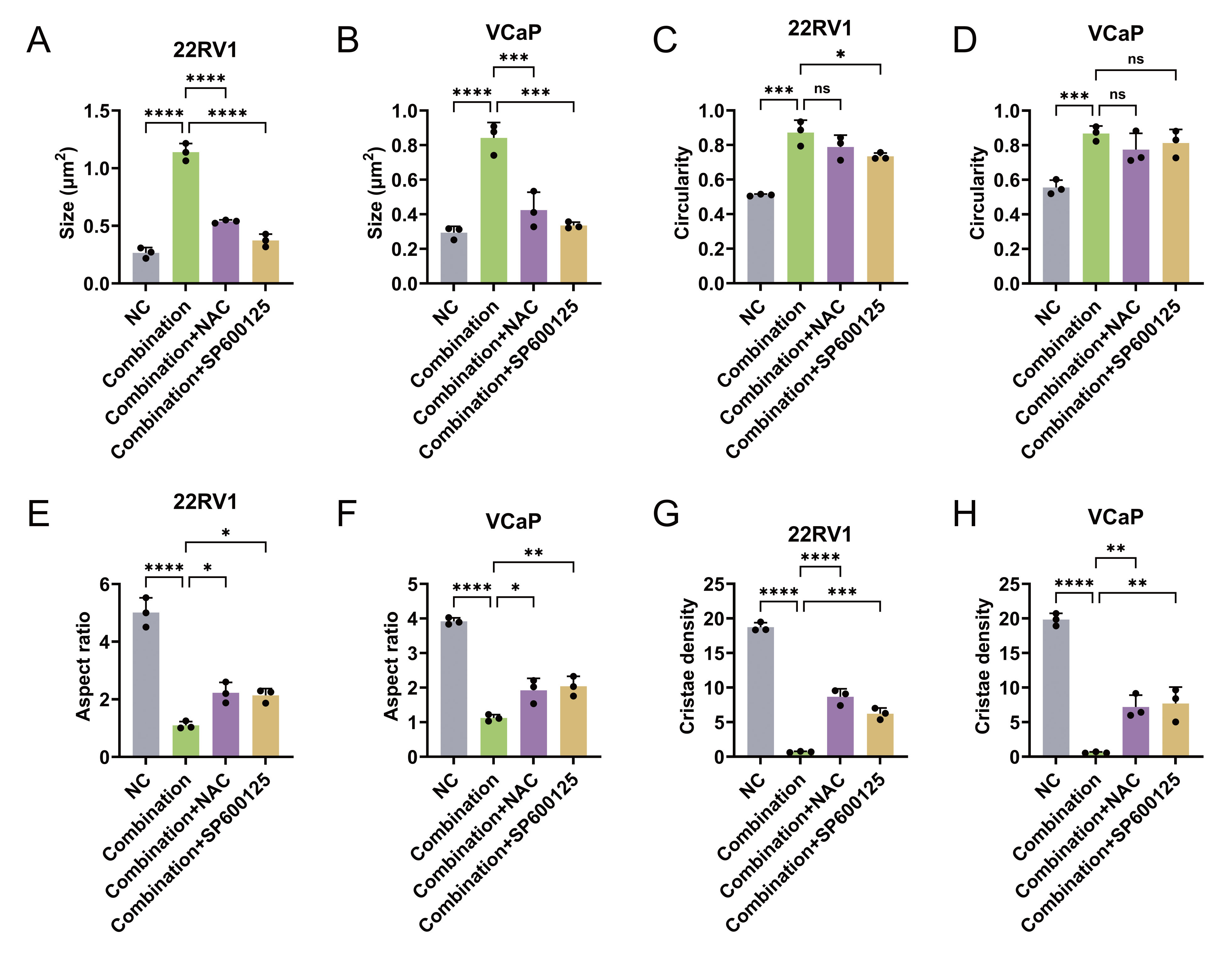
**Figure S19. Statistical analyses of mitochondrial morphological changes induced by the addition of NAC and SP600125.** Quantitative statistical analyses of mitochondrial morphology were performed, including (A-B) size, (C-D) circularity, (E-F) aspect ratio, and (G-H) cristae density. The results revealed that NAC and SP600125 also reversed the changes in mitochondrial morphology, with reduced mitochondrial swelling and partial restoration of cristae density. Data were presented as the mean ± SD from three biological replicates. The Brown–Forsythe test *P* values were all > 0.05, satisfying the homogeneity of variance assumption. One-way ANOVA with Turkey multiple comparison corrections were applied. NAC, N-acetylcysteine; *, *P* < 0.05; **, *P* < 0.01; ***, *P* < 0.001; ****, *P* < 0.0001; ns, not significant.

**Table S1. All antibodies inflammation.**

| Target | Supplier | Dilution ratio |
| --- | --- | --- |
| AKT | Cell Signaling Technology (4691T) | 1:1000 |
| AR | Cell Signaling Technology (5153T) | 1:2000 |
| AR-V7 | Cell Signaling Technology (19672T) | 1:1000 |
| BAX | Proteintech (50599-2-Ig) | 1:5000 |
| Bcl-2 | Proteintech (12789-1-AP) | 1:5000 |
| Caspase-3/P17P19 | Proteintech (19677-1-AP) | 1:1000 |
| CAT | Proteintech (21260-1-AP) | 1:5000 |
| c-Jun | Cell Signaling Technology (9165T) | 1:1000 |
| ERK1/2 | Cell Signaling Technology (9102T) | 1:1000 |
| GAPDH | Proteintech (60004-1-Ig) | 1:50000 |
| JNK | Cell Signaling Technology (9252T) | 1:1000 |
| KEAP1 | Selleck (F0484) | 1:1000 |
| NRF2 | Abcam (ab137550) | 1:2000 |
| PARP | Proteintech (80174-1-RR) | 1:5000 |
| PDGFA | Bioss (bs-0196R) | 1:2000 |
| Phospho-AKT (Ser473) | Cell Signaling Technology (4060T) | 1:2000 |
| Phospho-c-Jun (Ser73) | Cell Signaling Technology (3270T) | 1:1000 |
| Phospho-ERK1/2(Thr202/Tyr204) | Cell Signaling Technology (4370T) | 1:2000 |
| Phospho-JNK (Thr183/Tyr185) | Cell Signaling Technology (4668T) | 1:1000 |
| Phospho-NRF2 (Ser40) | Selleck (F1552) | 1:1000 |
| RRAS | Proteintech (27457-1-AP) | 1:5000 |
| Secondary antibody (Anti-rabbit) | ZSGB-BIO (ZB-2306) | 1:5000 |
| Secondary antibody (Anti-mouse) | ZSGB-BIO (ZB-2305) | 1:5000 |
| Ubiquitin | Proteintech (10201-2-AP) | 1:5000 |

**Table S3. Drug concentration used in xenograft model.**

| Drug | Concentration (mg/kg) |
| --- | --- |
| Enzalutamide | 20.00 |
| ARV-110 | 10.00 |
| JNJ10198409 | 10.00 |
| Combination | 10.00 (ARV-110) |
|  | 10.00 (JNJ10198409) |
| Combination+NAC | 10.00 (ARV-110) |
|  | 10.00 (JNJ10198409) |
|  | 150.00 (NAC) |
| Combination+SP600125 | 10.00 (ARV-110) |
|  | 10.00 (JNJ10198409) |
|  | 30.00 (SP600125) |

NAC, N-acetylcysteine.

**Table S5. PCR primer sequences.**

|  | Forward (5’-3’) | Reverse (5’-3’) |
| --- | --- | --- |
| AR | CCAGGGACCATGTTTTGCC | CGAAGACGACAAGATGGACAA |
| PDGFR-α | TTTTTGTGACGGTCTTGGAAGT | TGTCTGAGTGTGGTTGTAATAGC |
| PDGFR-β | AGACACGGGAGAATACTTTTGC | AGTTCCTCGGCATCATTAGGG |
| GAPDH | GGCTGTTGTCATACTTCTCATGG | GGAGCGAGATCCCTCCAAAAT |
| PDGFA | GCAAGACCAGGACGGTCATTT | GGCACTTGACACTGCTCGT |
| PDGFB | CTCGATCCGCTCCTTTGATGA | CGTTGGTGCGGTCTATGAG |
| PDGFC | ATTCACAGCCCAAGGTTTCCT | GGGTCTTCAAGCCCAAATCTT |
| PDGFD | TTGTACCGAAGAGATGAGACCA | GCTGTATCCGTGTATTCTCCTGA |
| NRF2 | TCAGCGACGGAAAGAGTATGA | CCACTGGTTTCTGACTGGATGT |
| CAT | TGGAGCTGGTAACCCAGTAGG | CCTTTGCCTTGGAGTATTTGGTA |
| ChIP (AR-PDGFA) | AACGTGGAGGAGCCCCTTA | CTGCGAGTCCGGCTCTTTC |
| ChIP (AR-CAT) | TGTAAGATCAAGAACAACATGTGAA | TCCAGTACAACTACATATAATGGGT |

**Table S6. AR variants in 22RV1 and VCaP cells.**

| Cell | AR variants |
| --- | --- |
| 22RV1 | AR-V1, AR-V2, AR-V3, AR-V4, AR-V5, AR-V6, AR-V7, AR-V9, AR-V12 |
| VCaP | AR-V1, AR-V3, AR-V7, AR-V8, AR-V9, AR-V10, AR-V11, ARv567es |

**Table S7. Calculation of CI values in CRPC cells**

| Cell | Combination | CI values | | | |
| --- | --- | --- | --- | --- | --- |
|  |  | ED50 | ED75 | ED90 | ED95 |
| 22RV1 | ARV-110+AKBA | 1.02 | 0.99 | 1.07 | 1.15 |
|  | ARV-110+Bemcentinib | 0.90 | 0.75 | 0.72 | 0.71 |
|  | ARV-110+Bexarotene | 1.29 | 1.12 | 1.06 | 1.05 |
|  | ARV-110+‌Gossypol-acetic acid | 2.14 | 2.31 | 3.21 | 4.27 |
|  | ARV-110+GZD824 Dimesylate | 0.89 | 0.81 | 0.77 | 0.76 |
|  | ARV-110+HM43239 | 1.13 | 0.93 | 0.85 | 0.83 |
|  | ARV-110+Ivermectin | 1.34 | 1.19 | 1.22 | 1.26 |
|  | ARV-110+Lusutrombopag | 1.36 | 1.23 | 1.23 | 1.26 |
|  | ARV-110+Ponatinib | 0.68 | 0.44 | 0.39 | 0.37 |
|  | ARV-110+Saikosaponin D | 1.03 | 0.90 | 0.87 | 0.85 |
|  | ARV-110+Sanguinarine chloride | 0.81 | 0.84 | 0.94 | 1.03 |
|  | ARV-110+Siramesine HCL | 1.80 | 1.37 | 1.04 | 0.87 |
|  | ARV-110+Teaserod Maleate | 1.01 | 0.92 | 0.99 | 1.06 |
| VCaP | ARV-110+Ponatinib | 0.59 | 0.30 | 0.18 | 0.13 |

CI, combination index.

**Table S8. Calculation of CI values of ARV-110 combined with JNJ10198409 in CRPC cells**

| Cell | Combination | CI values | | | |
| --- | --- | --- | --- | --- | --- |
|  |  | ED50 | ED75 | ED90 | ED95 |
| 22RV1 | ARV-110+JNJ10198409 | 0.13 | 0.20 | 0.33 | 0.46 |
| VCaP | ARV-110+JNJ10198409 | 0.59 | 0.33 | 0.20 | 0.15 |

CI, combination index.

**Table S9. Drug concentrations in subsequent analyses (partial concentrations were approximated for ease of preparation.).**

| Cell | NC | ARV-110 | JNJ10198409 | Combination strategy |
| --- | --- | --- | --- | --- |
| 22RV1 | 0.00 μM | 0.20 μM | 0.10 μM | 0.03 μM  (including ARV-110 0.02 μM +JNJ10198409 0.01 μM) |
| VCaP | 0.00 μM | 2.50 μM | 4.00 μM | 2.50 μM  (including ARV-110 1.00 μM +JNJ10198409 1.50 μM) |

**Table S10. Determination of MTC of enzalutamide in zebrafish.**

| Group | Concentration (μM) | Number of deaths | State |
| --- | --- | --- | --- |
| NC | - | 0 | Normal |
| Enzalutamide | 0.39 | 0 | Similar to NC |
|  | 0.78 | 0 | Similar to NC |
|  | 1.56 | 0 | Similar to NC |
|  | 3.12^*^ | 0 | Similar to NC |
|  | 6.25 | 0 | Worse than NC |
|  | 12.50 | 0 | Worse than NC |
|  | 25.00 | 0 | Worse than NC |
|  | 50.00 | 20 | - |

MTC, maximum tolerated concentration.

*, MTC.

**Table S11. Determination of MTC of ARV-110 in zebrafish.**

| Group | Concentration (μM) | Number of deaths | State |
| --- | --- | --- | --- |
| NC | - | 0 | Normal |
| ARV-110 | 0.39 | 0 | Similar to NC |
|  | 0.78 | 0 | Similar to NC |
|  | 1.56 | 0 | Similar to NC |
|  | 3.12^*^ | 0 | Similar to NC |
|  | 6.25 (precipitation) | 0 | Similar to NC |
|  | 12.50 (precipitation) | 0 | Similar to NC |
|  | 25.00 (precipitation) | 0 | Similar to NC |
|  | 50.00 (precipitation) | 0 | Similar to NC |

MTC, maximum tolerated concentration.

*, MTC.

**Table S12. Determination of MTC of JNJ10198409 in zebrafish.**

| Group | Concentration (μM) | Number of deaths | State |
| --- | --- | --- | --- |
| NC | - | 0 | Normal |
| JNJ10198409 | 0.39 | 0 | Similar to NC |
|  | 0.78 | 0 | Similar to NC |
|  | 1.56^*^ | 0 | Similar to NC |
|  | 3.12 | 0 | Worse than NC |
|  | 6.25 | 17 | Worse than NC |
|  | 12.5 | 20 | - |
|  | 25.00 | 20 | - |
|  | 50.00 | 20 | - |

MTC, maximum tolerated concentration.

*, MTC.

**Table S13. Determination of MTC of Combination in zebrafish.**

| Group | Concentration (μM) | Number of deaths | State |
| --- | --- | --- | --- |
| NC | - | 0 | Normal |
| Combination | 3.12+0.39 | 0 | Similar to NC |
|  | 3.12+0.78 | 0 | Similar to NC |
|  | 3.12+1.56^*^ | 0 | Similar to NC |

MTC, maximum tolerated concentration; Combination, ARV-110+JNJ10198409.

*, MTC.

**Table S14. Wild-type and mutant sequences of the best binding site between AR and PDGFA promoter.**

| Promoter site | Start | End | Wild type | Mutated type |
| --- | --- | --- | --- | --- |
| Chromosome 7:  520700-522700 | 745 | 759 | GGGGACACCCACTGC | AAAAGAGTTTGTGAA |

AR, androgen receptor; PDGFA, platelet-derived growth factor A.

**Table S15. Wild-type and mutant sequences of the best binding site between AR and CAT promoter.**

| Promoter site | Start | End | Wild type | Mutated type |
| --- | --- | --- | --- | --- |
| Chromosome 11:  34436934-34438934 | 935 | 949 | AAGAACAACATGTGA | GGACCTGGACG  TCTG |

AR, androgen receptor; CAT, catalase.

**Table S16. Determination of MTC of NAC in zebrafish.**

| Group | Concentration (μM) | Number of deaths | State |
| --- | --- | --- | --- |
| NC | - | 0 | Normal |
| NAC | 0.39 | 0 | Similar to NC |
|  | 0.78 | 0 | Similar to NC |
|  | 1.56 | 0 | Similar to NC |
|  | 3.12 | 0 | Similar to NC |
|  | 6.25 | 0 | Similar to NC |
|  | 12.50 | 0 | Similar to NC |
|  | 25.00 | 0 | Similar to NC |
|  | 50.00^*^ | 0 | Similar to NC |

MTC, maximum tolerated concentration; NAC, N-acetylcysteine.

*, MTC.

**Table S17. Determination of MTC of SP600125 in zebrafish.**

| Group | Concentration (μM) | Number of deaths | State |
| --- | --- | --- | --- |
| NC | - | 0 | Normal |
| SP600125 | 0.39 | 0 | Similar to NC |
|  | 0.78 | 0 | Similar to NC |
|  | 1.56 | 0 | Similar to NC |
|  | 3.12 | 0 | Similar to NC |
|  | 6.25 | 0 | Similar to NC |
|  | 12.50^*^ | 0 | Similar to NC |
|  | 25.00 | 0 | Worse than NC |
|  | 50.00 | 0 | Worse than NC |

MTC, maximum tolerated concentration.

*, MTC.

**Table S18. Determination of MTC of Combination+NAC in zebrafish.**

| Group | Concentration (μM) | Number of deaths | State |
| --- | --- | --- | --- |
| NC | - | 0 | Normal |
| Combination+NAC | 3.12+1.56+0.39 | 0 | Similar to NC |
|  | 3.12+1.56+0.78 | 0 | Similar to NC |
|  | 3.12+1.56+1.56 | 0 | Similar to NC |
|  | 3.12+1.56+3.12 | 0 | Similar to NC |
|  | 3.12+1.56+6.25 | 0 | Similar to NC |
|  | 3.12+1.56+12.50 | 0 | Similar to NC |
|  | 3.12+1.56+25.00 | 0 | Similar to NC |
|  | 3.12+1.56+50.00^*^ | 0 | Similar to NC |

MTC, maximum tolerated concentration; Combination, ARV-110+JNJ10198409.

*, MTC.

**Table S19. Determination of MTC of Combination+SP600125 in zebrafish.**

| Group | Concentration (μM) | Number of deaths | State |
| --- | --- | --- | --- |
| NC | - | 0 | Normal |
| Combination  +SP600125 | 3.12+1.56+0.39 | 0 | Similar to NC |
|  | 3.12+1.56+0.78 | 0 | Similar to NC |
|  | 3.12+1.56+1.56 | 0 | Similar to NC |
|  | 3.12+1.56+3.12 | 0 | Similar to NC |
|  | 3.12+1.56+6.25 | 0 | Similar to NC |
|  | 3.12+1.56+12.50^*^ | 0 | Similar to NC |

MTC, maximum tolerated concentration; Combination, ARV-110+JNJ10198409.

*, MTC.
