# Supplementary material for: Mechanism and therapeutic significance of ARV-110 combined with a PDGFR inhibitor for the induction of apoptosis in castration-resistant prostate cancer cells through the ROS/JNK pathway
Source: Cell Death Dis. 2026 Apr 10;17(1):463. doi: 10.1038/s41419-026-08718-w (PMC13181112; doi:10.1038/s41419-026-08718-w)
Supplement: Supplementary file 4 — Original Western figures [file 41419_2026_8718_MOESM4_ESM.docx]

**Figure 1A**

**AR and AR-variants AR-V7 GAPDH**


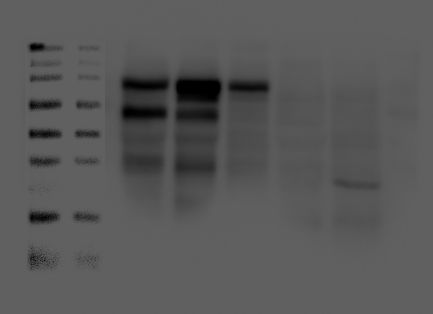

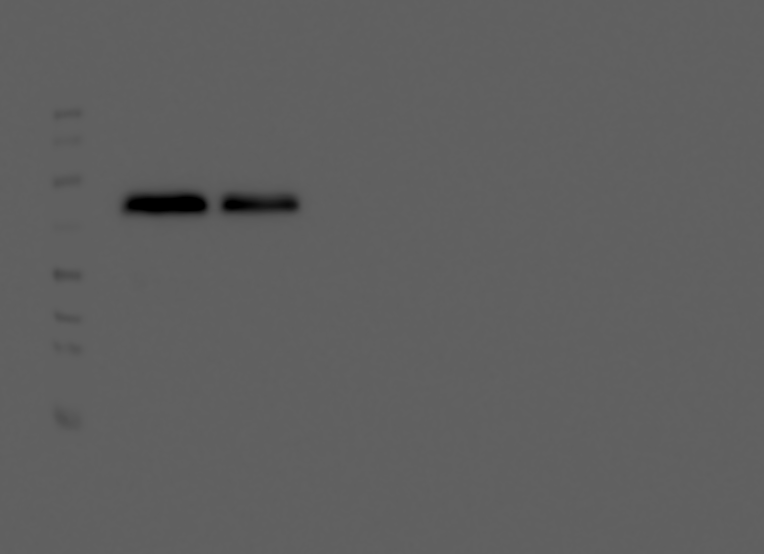

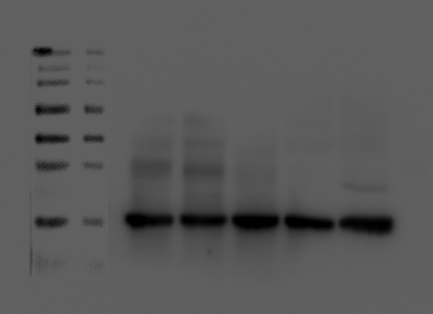


**Figure 1B**

**AR and AR-variants AR-V7 GAPDH**


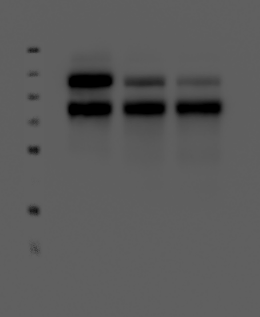

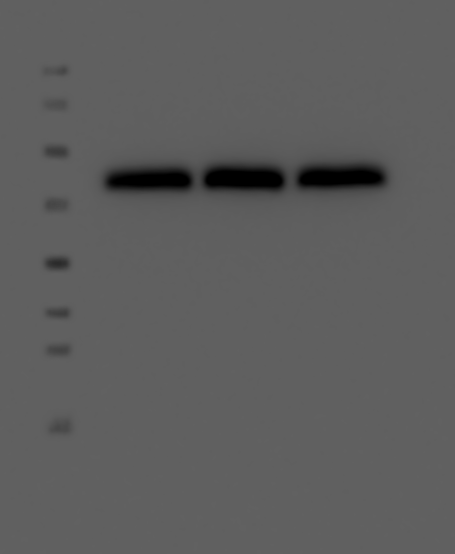

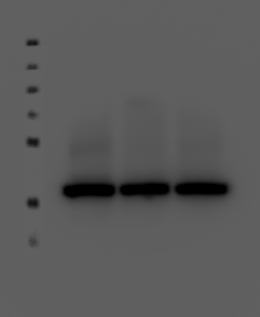


**AR and AR-variants AR-V7 GAPDH**


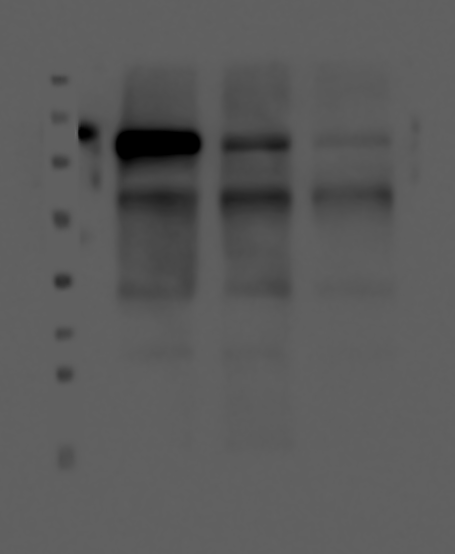

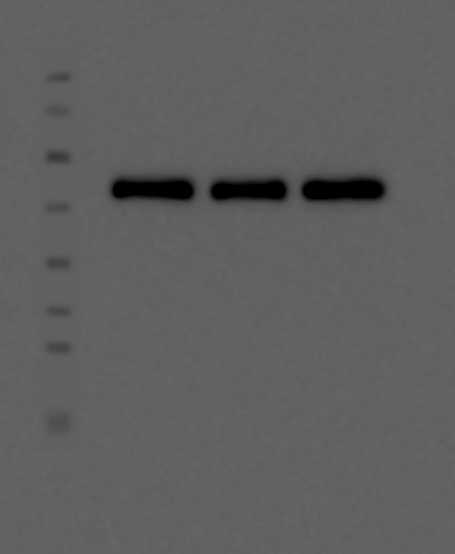

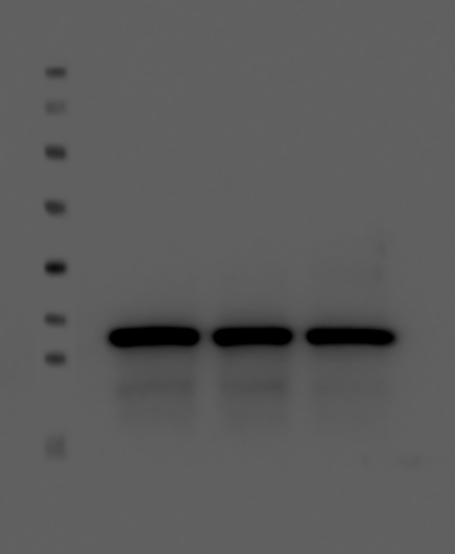


**Figure 1C**

**AR and AR-variants AR-V7 GAPDH**


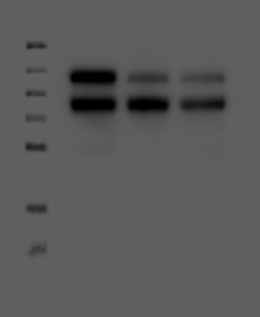

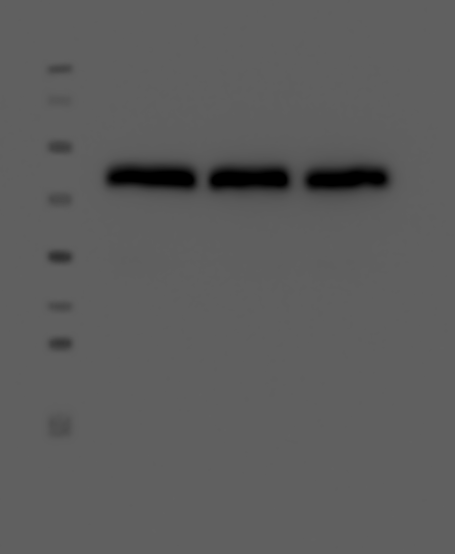

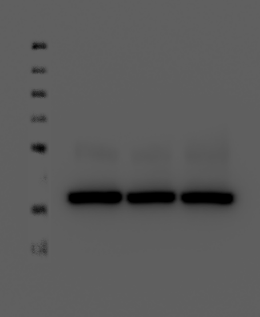


**AR and AR-variants AR-V7 GAPDH**


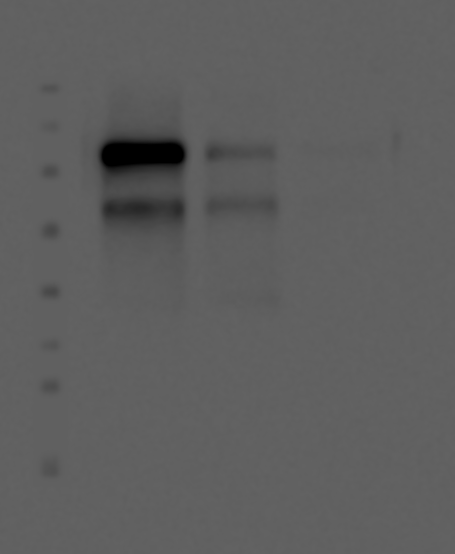

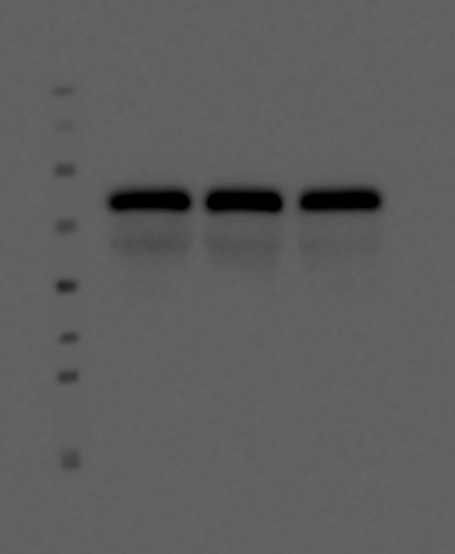

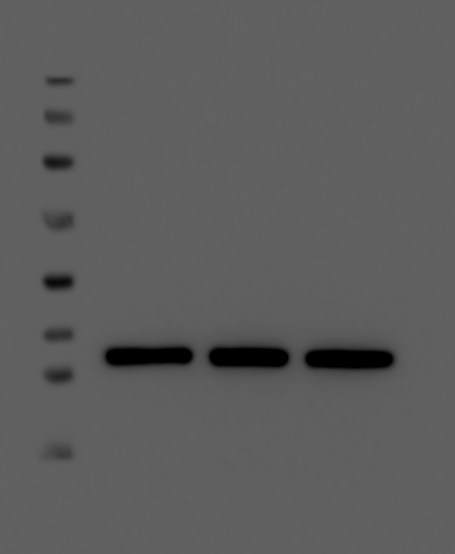


**Figure 2K**

**AR GAPDH**


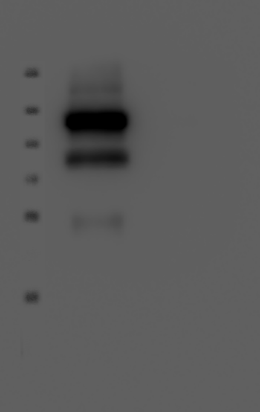

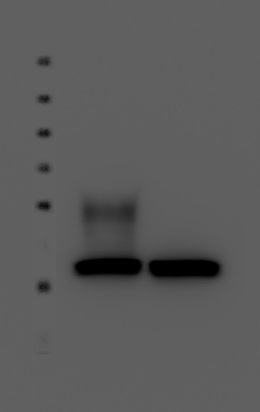


**Figure 2M**

**ERK1/2**  **Phospho-ERK1/2 ERK1/2 Phospho-ERK1/2**


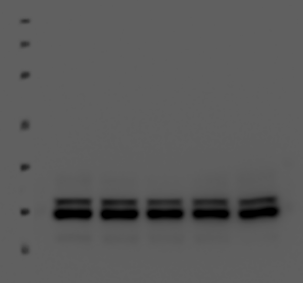

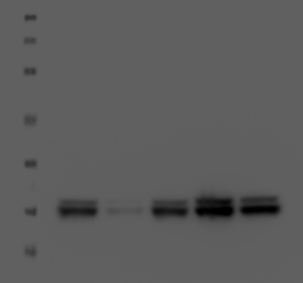

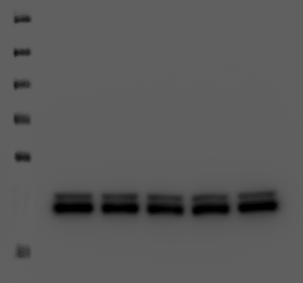

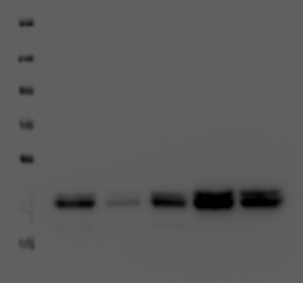


**AKT Phospho-AKT AKT Phospho-AKT**


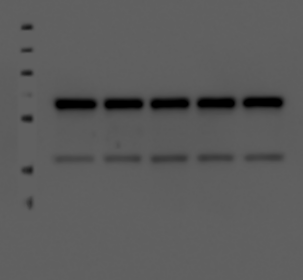

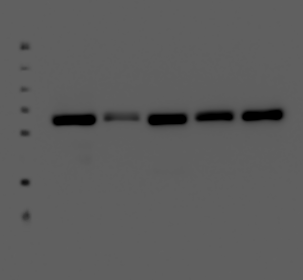

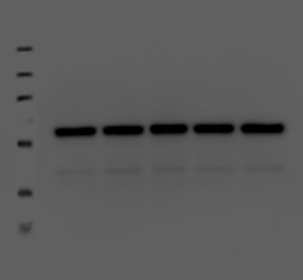

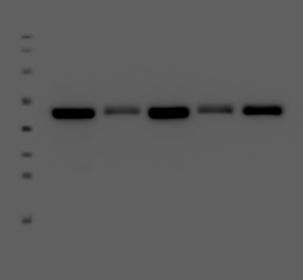


**GAPDH GAPDH**


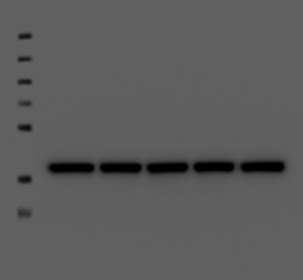

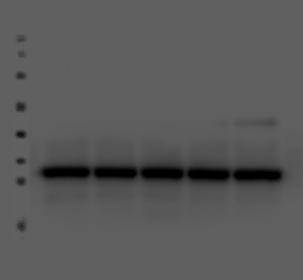


**Figure 3D**

**PARP PARP**


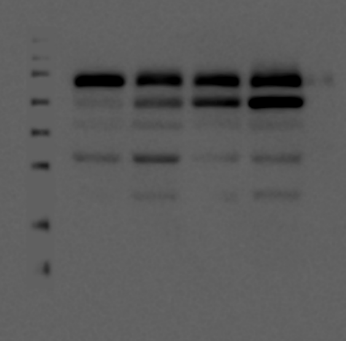

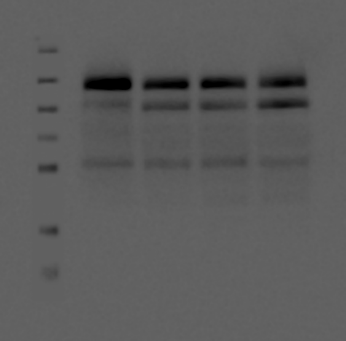


**BAX BAX**


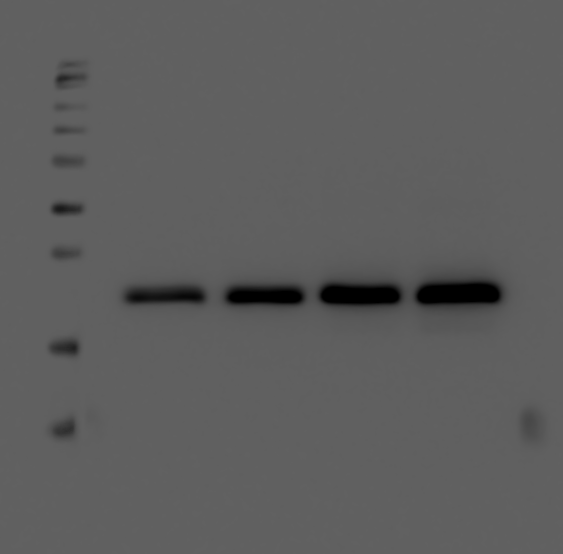

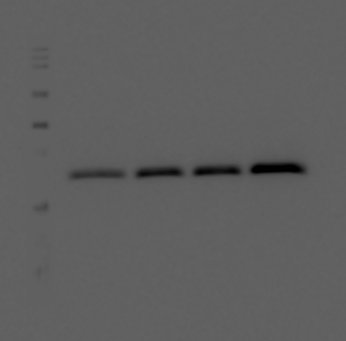


**Bcl-2 Bcl-2**


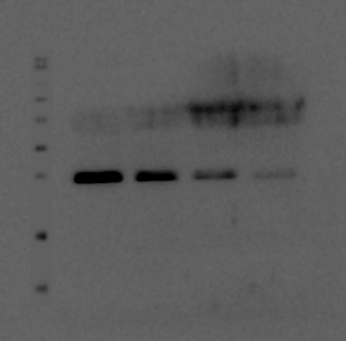

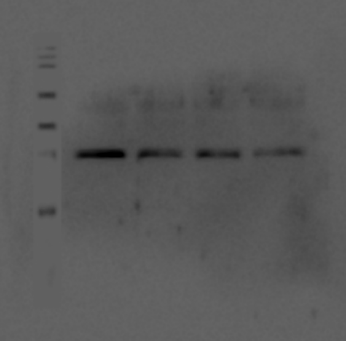


**Caspase-3 Cleaved Caspase-3 Caspase-3 Cleaved Caspase-3**


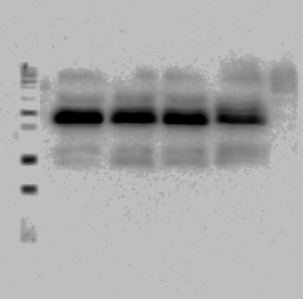

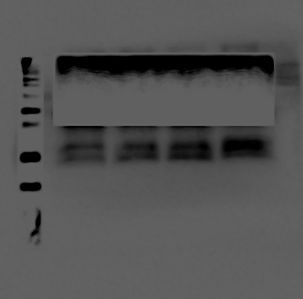

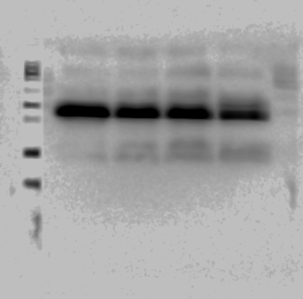

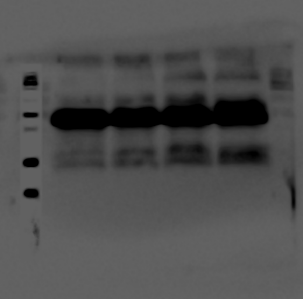


**GAPDH GAPDH**


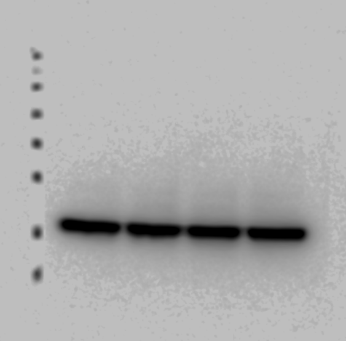

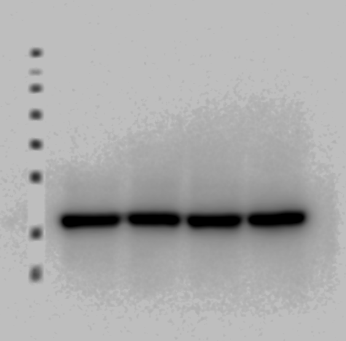


**Figure 6D**

**AKT Phospho-AKT AKT Phospho-AKT**


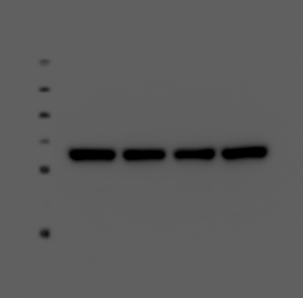

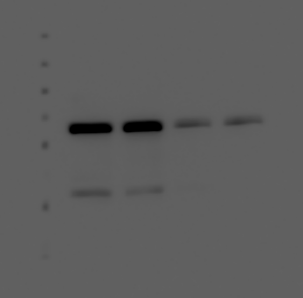

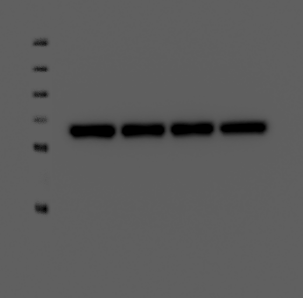

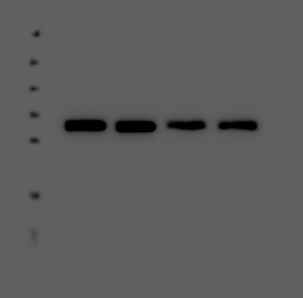


**NRF2 Phospho-NRF2 NRF2 Phospho-NRF2**


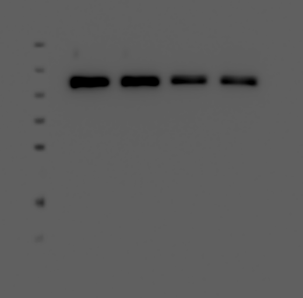

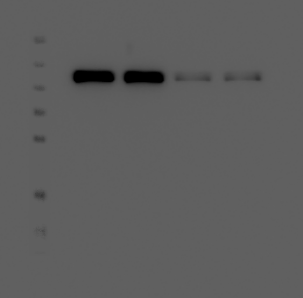

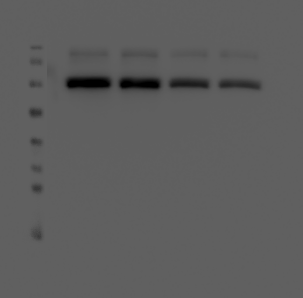

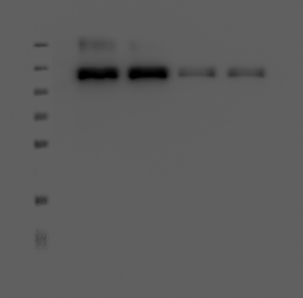


**CAT CAT**


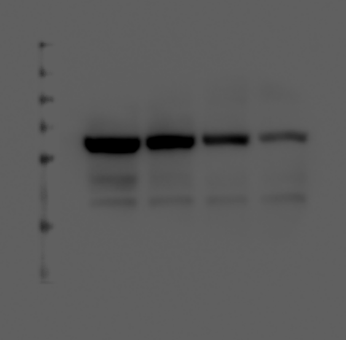

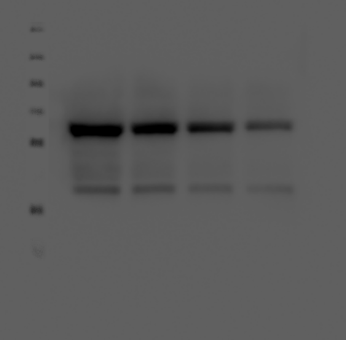


**GAPDH GAPDH**


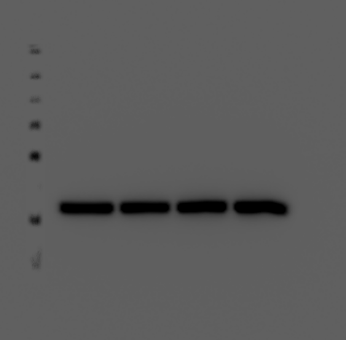

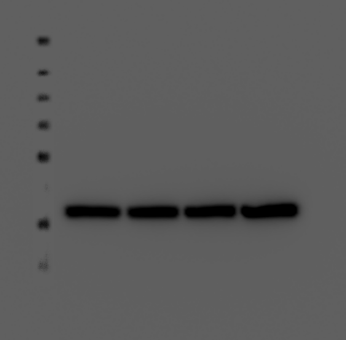


**Figure 6E**

**AKT Phospho-AKT AKT Phospho-AKT**


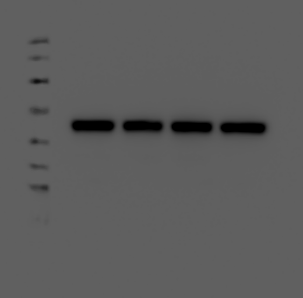

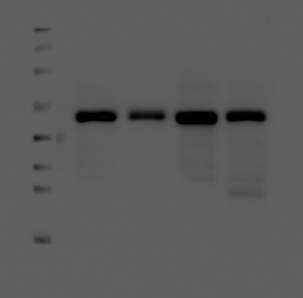

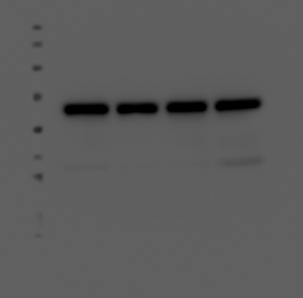

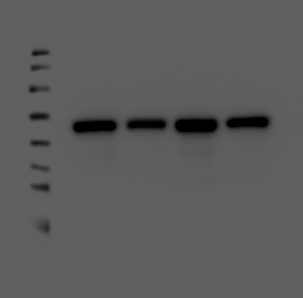


**NRF2 Phospho-NRF2 NRF2 Phospho-NRF2**


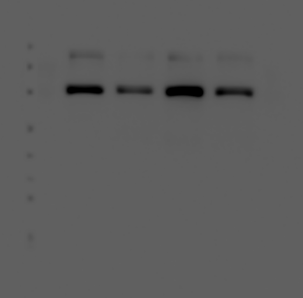

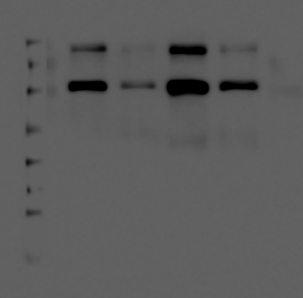

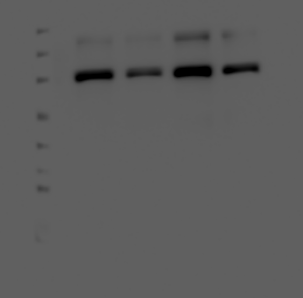

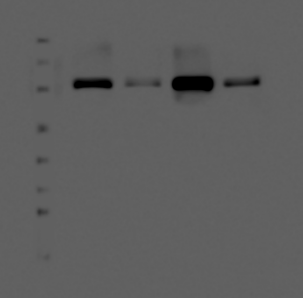


**CAT CAT**


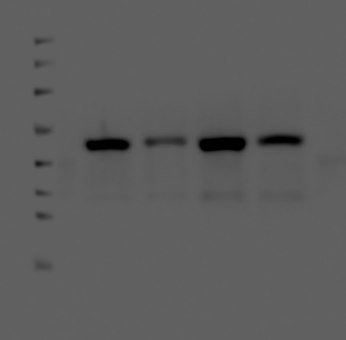

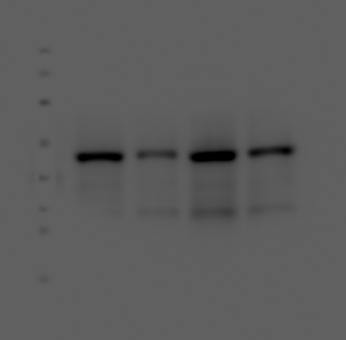


**GAPDH GAPDH**


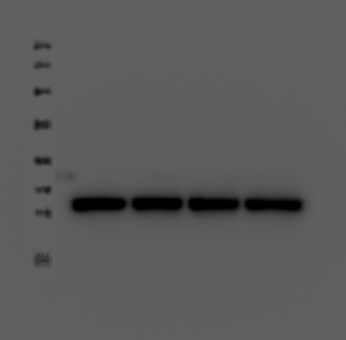

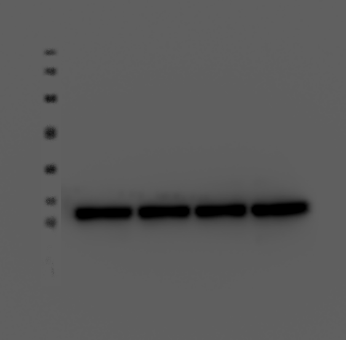


**Figure 6H**

**NRF2 NRF2**


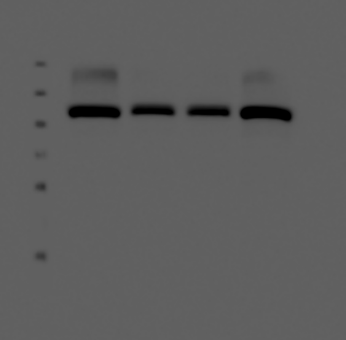

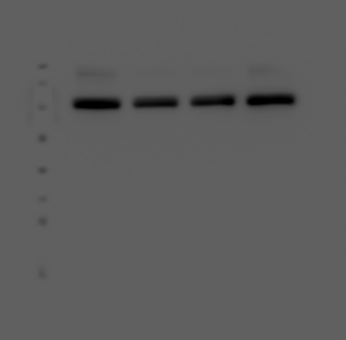


**GAPDH GAPDH**


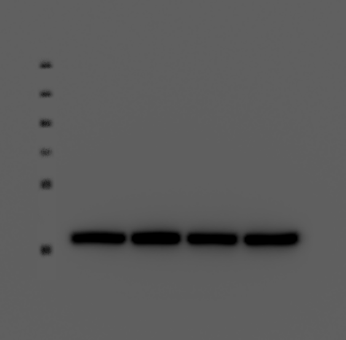

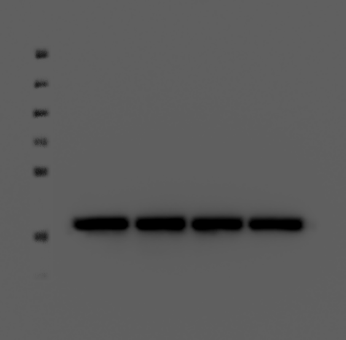


**Figure 6I**

**NRF2 NRF2**


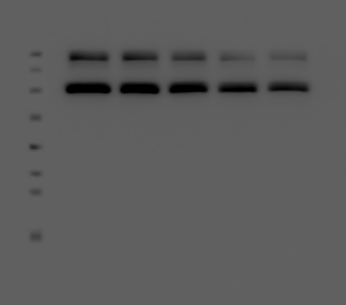

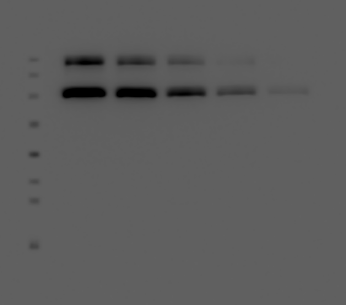


**GAPDH GAPDH**


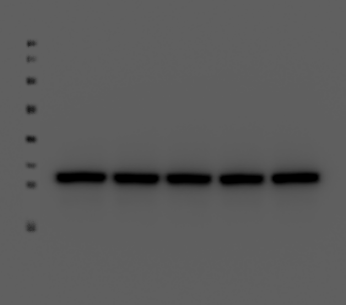

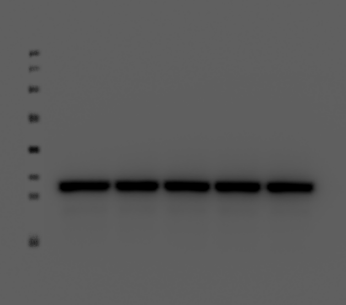


**Figure 6K**

**NRF2 NRF2**


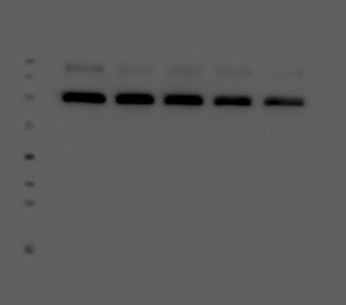

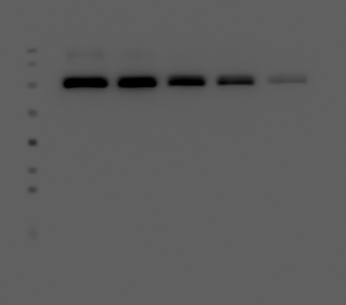


**GAPDH GAPDH**


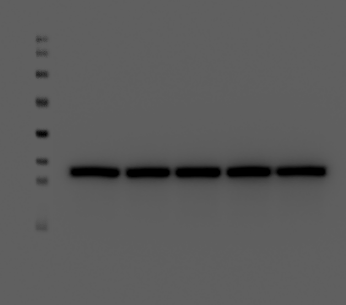

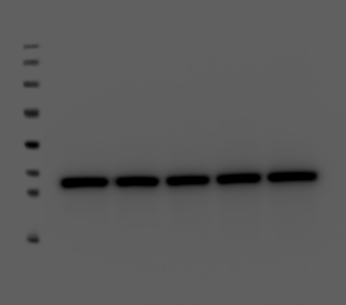


**Figure 6M**

**Ub Ub**


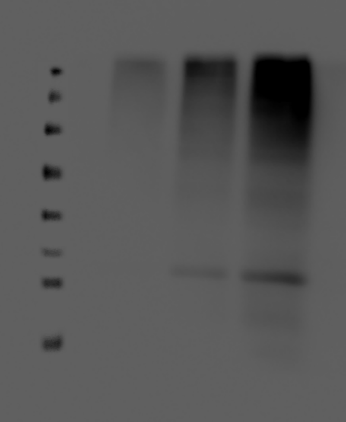

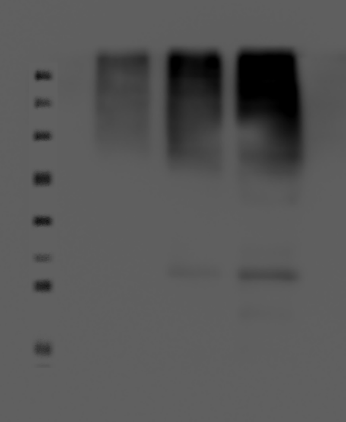


**KEAP1 KEAP1**

**
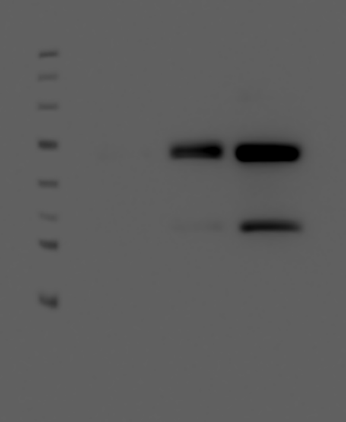
**
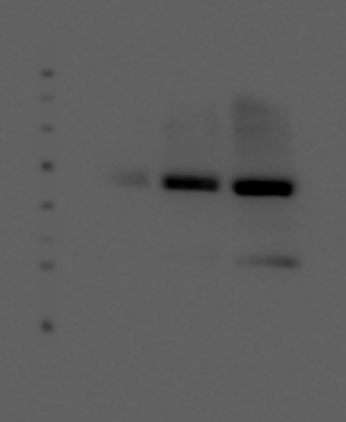


**NRF2 NRF2**

**
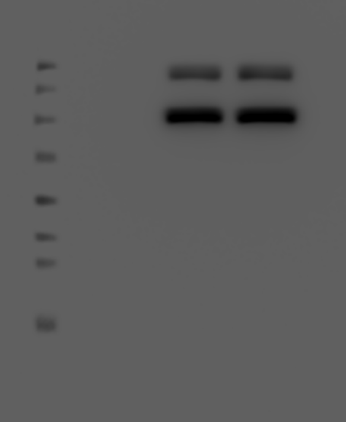
**
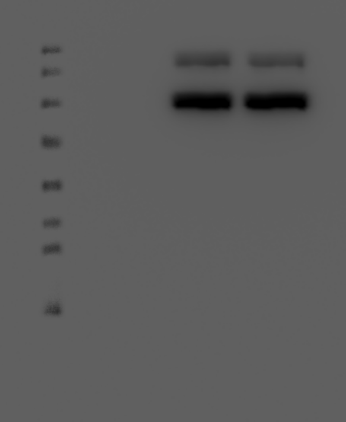


**NRF2 NRF2**

**
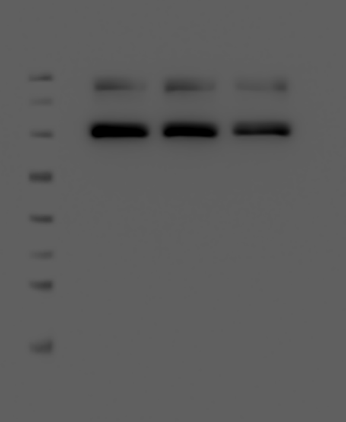
**
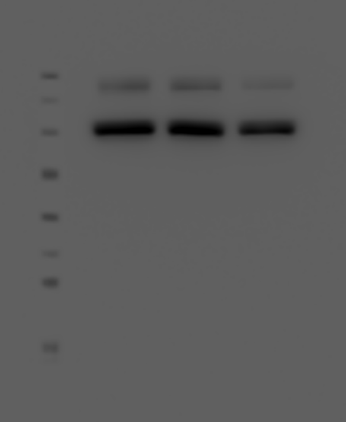


**GAPDH GAPDH**


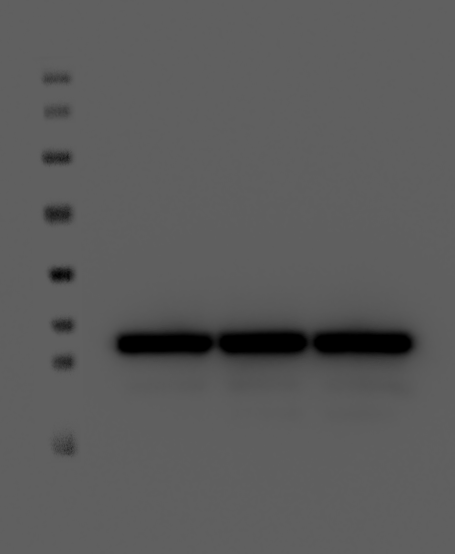

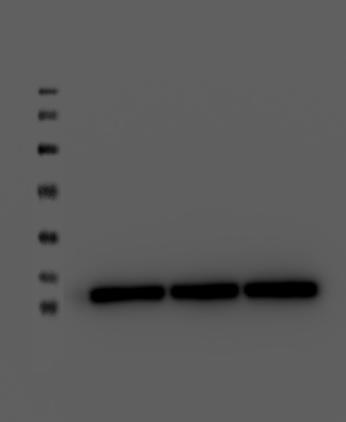


**Figure 7J**

**PARP PARP**


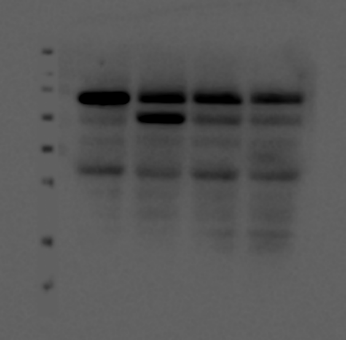

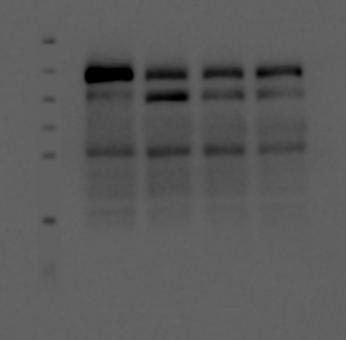


**BAX BAX**


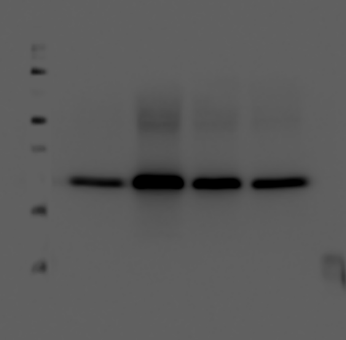

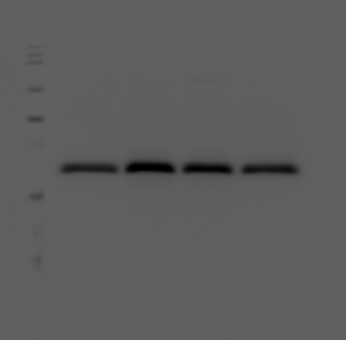


**Bcl-2 Bcl-2**


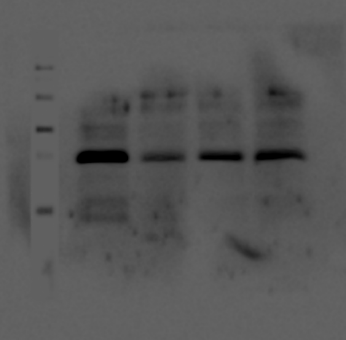

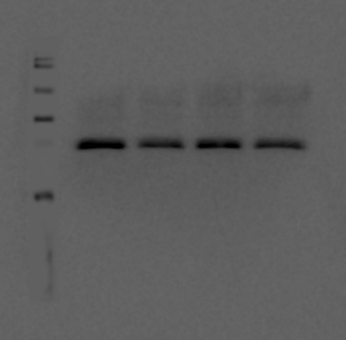


**Caspase-3 Cleaved Caspase-3 Caspase-3 Cleaved Caspase-3**


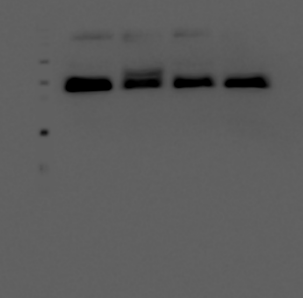

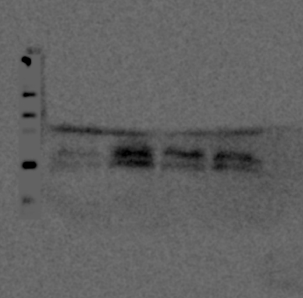

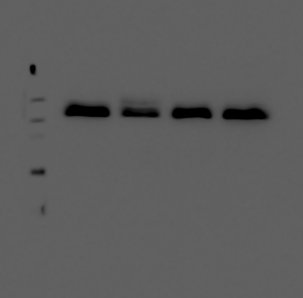

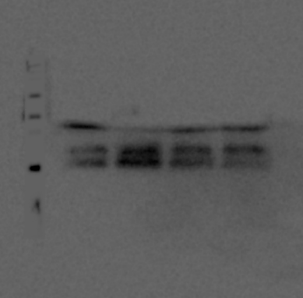


**GAPDH GAPDH**


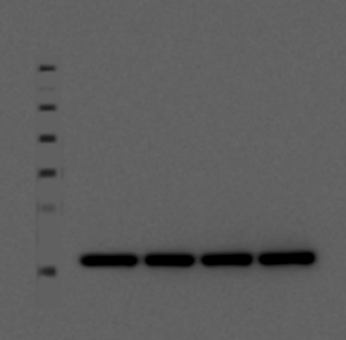

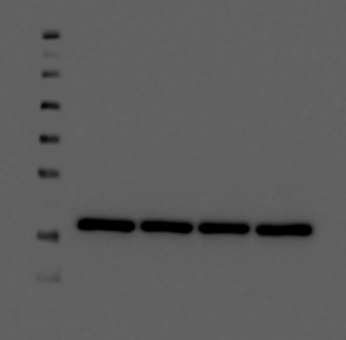


**Figure 7K**

**RRAS RRAS**


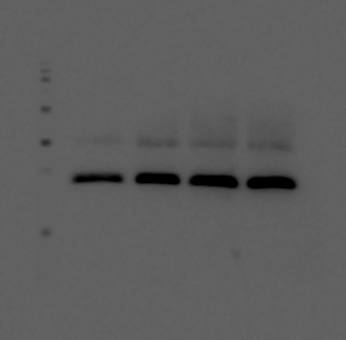

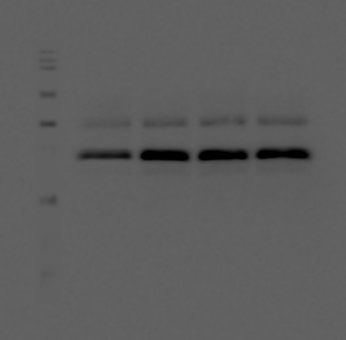


**JNK Phospho-JNK JNK Phospho-JNK**


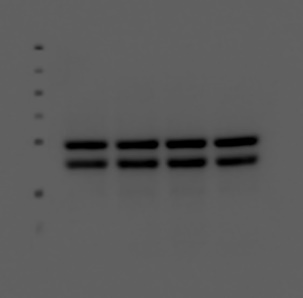


**c-Jun Phospho-** **c-Jun c-Jun Phospho-** **c-Jun**

**GAPDH GAPDH**

**Figure S8G**

**AR AR**

**PDGFA PDGFA**

**GAPDH GAPDH**

**Figure S10I**

**RRAS RRAS**

**JNK Phospho-JNK JNK Phospho-JNK**

**c-Jun Phospho-** **c-Jun c-Jun Phospho-** **c-Jun**

**GAPDH GAPDH**

**Figure S10J**

**RRAS GAPDH RRAS GAPDH**

**Figure S10K**

**RRAS GAPDH RRAS GAPDH**

**Figure S10L**

**RRAS RRAS**

**JNK JNK**

**Phospho-JNK Phospho-JNK**

**c-Jun c-Jun**

**Phospho-c-Jun Phospho-c-Jun**

**GAPDH GAPDH**

**Figure S10M**

**RRAS RRAS**

**JNK JNK**

**Phospho-JNK Phospho-JNK**

**c-Jun c-Jun**

**Phospho-c-Jun Phospho-c-Jun**

**GAPDH**
